# Asymmetric Synthesis of 1,3-Oxazolidine Derivatives with Multi-Component Reaction and Research of Kinetic Resolution

**DOI:** 10.3390/molecules200917208

**Published:** 2015-09-18

**Authors:** Xiao-Wei Hong, Yu-Qiang Zhou, Cui-Bing Bai, Nai-Xing Wang, Yalan Xing, Wei Zhang, Yan-Jing Wang, Xing-Wang Lan, Yu Xie, Yi-He Li

**Affiliations:** 1 Technical Institute of Physics and Chemistry, Chinese Academy of Sciences, Beijing 100080, China; E-Mails: hongxiao86@126.com (X.-W.H.); z1121@126.com (Y.-Q.Z.); baicuibing@126.com (C.-B.B.) zhjp271@163.com (W.Z.); wangyanjing33@hotmail.com (Y.-J.W.); hxlxw@sina.cn (X.-W.L.); yihenim@163.com (Y.-H.L.); 2 College of Environment and Chemical Engineering, Nanchang Hangkong University, Nanchang 330063, China; 3 Department of Chemistry, William Paterson University of New Jersey, 300 Pompton Road, Wayne, NJ 07470, USA

**Keywords:** 1,3-oxazolidine, asymmetric synthesis, multi-component reaction, kinetic resolution, synthetic methods

## Abstract

An efficient multi-component reaction to synthesize multi-substituted 1,3-oxazolidine compounds of high optical purity was described. All the products were well-characterized and the absolute configuration of one chiral center was determined. The plausible mechanism was proposed and a kinetic resolution of epoxides process was confirmed.

## 1. Introduction

The value of 1,3-oxazolidine derivatives, which are found in many natural products and synthetic complex compounds, lies in their utility in drugs, agrochemicals, dyes, and organic synthesis of a wide range of biologically-important compounds [1,2,3,4,5,6,7,8,9,10]. Thus, highly efficient and creative synthetic methods of 1,3-oxazolidine skeleton is an attractive topic in organic synthesis [11,12,13]. As a result, more and more efficient syntheses of 1,3-oxazolidine derivatives were reported utilizing intramolecular cyclization and intermolecular cycloaddition [14,15]. Cascade reaction, an ecologically- and economically-favorable method, was considered to fall under the banner of “green chemistry” because of its atom and resource economy [16,17,18,19], as well as time efficiency [20,21,22,23,24,25,26,27,28,29]. However, cascade reaction has not been used to construct 1,3-oxazolidine skeleton up to date. Application of more efficient multicomponent cascade reactions to replace traditional single-step-procedure reactions in constructing 1,3-oxazolidine skeleton is still an interesting topic.

On the other hand, kinetic resolution, a powerful strategy in asymmetric synthesis, makes racemic substrates into optically-pure compounds [30,31,32,33,34,35,36,37,38,39,40,41]. Although many methods towards 1,3-oxazolidine skeletons have been developed, asymmetric synthesis of 1,3-oxazolidine with three components reaction involving kinetic resolution has not been well studied [42,43,44,45,46,47,48,49,50,51,52,53].

The reaction of imine with aldehyde is a well-established one, for example, Ishii group described a method to synthesize 1,3-oxazolidines from a multi-step synthesis of imines and epoxides [13]. However, the reaction yields were relatively low and the stereochemistry was not explained. Herein, we describe an efficient multi-component reaction to access 1,3-oxazolidine compounds of high optical purity. The plausible mechanism of the reaction was suggested and a kinetic resolution of the epoxides process was confirmed. All of products were well-characterized and the absolute configuration of the chiral center from the epoxides part was determined. To the best of our knowledge [54,55], this is the first report of synthesis of 1,3-oxazolidine with a multi-component reaction via the kinetic resolution from epoxides, anilines, and ethyl glyoxalate.

## 2. Results and Discussion

**Scheme 1:**
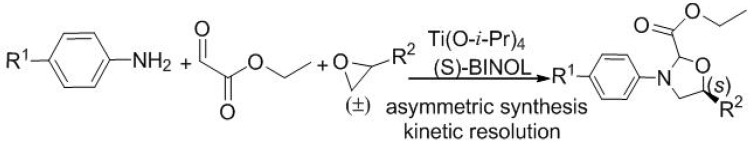
Asymmetric multi-component reaction of anilines, ethyl glyoxalate, and epoxides.

The formal references about 1,3-oxazolidine all required multi-step reactions [56,57,58,59,60,61]. When we investigated the reactions of anilines, ethyl glyoxalate, and epoxides by one-pot asymmetric multi-component reaction, products with 1,3-oxazolidine structure were obtained (Scheme 1).

**Figure 1:**
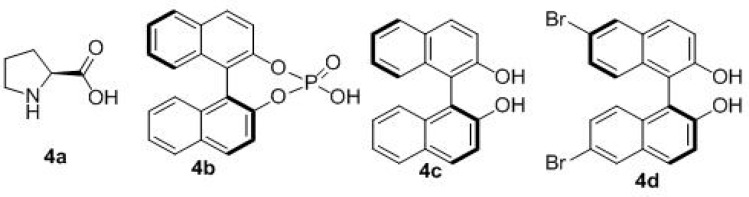
Chiral ligands used in this work.

The reaction conditions optimization of three-component reactions was carried out, a variety of different chiral Lewis acid (**4a**–**4d**) catalysts were screened (Figure 1).

First we examined the reaction of aniline **1a**, ethylglyoxalate, and epoxide **2a** in dichloromethane at 18 °C in the presence of catalyst **4a** (10 mol %), while the reaction gave the product in only 5% yield with poor diastereoselectivity and enantioselectivity after two days (Table 1, entry 1). Little higher yield and *ee* value were achieved when catalyst **4b** was applied (Table 1, entry 2). With this encouragement, the product with a higher *ee* was obtained when the reaction was carried out at −10 °C (Table 1, entry 3). Catalyst **4c**/Ti(IV), which was prepared by mixing **4c** and Ti(O-*i*-Pr)_4_ in 1:1 molar ratio in dichloromethane for 2 h, afforded a moderate *ee* of 41% (Table 1, entry 4). On the contrary, when the reaction was carried out with catalyst **4c**/Ti(IV), which was prepared in a 1:1 molar ratio in toluene, good diastereoselectivity and enantioselectivity trifluoroacetic acid (TFA) (0.5 mol %) was added, the product with a good yield was obtained (Table 1 were provided (Table 1, entry 5). To our delight, after a small amount of, entry 6). Therefore, it is indicated that TFA was very important for the cyclization. TFA might be beneficial for the ring-opening of epoxide. However, when TFA was used as the only catalyst, the reaction rate was not accelerated, and completion of the reaction also needed a long time at room temperature. Catalyst **4c**/Ti(IV) was prepared in a 2:1 molar ratio in toluene, affording better *ee* value (Table 1, entry 7). Slightly lower yield was observed for the reaction carried out with the catalyst **4d**/Ti(IV), which was prepared in a 2:1 molar ratio in toluene (Table 1, entry 8). The influence of temperature to the reaction was also investigated (Table 1, entries 9 and 10), the decrease of temperature were negative to the yields. It could be concluded that the molar ratio of **4c** and Ti(IV) influenced the enantioselectivity and TFA strongly benefited the yields.

**Figure 2:**
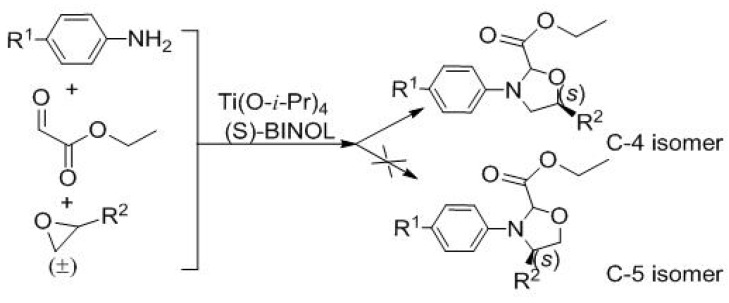
Isomer characterization of the product.

Theoretically, the products had C-5 isomer and C-4 isomer, but the C-4 isomer was only separated and actually detected when different reaction scales from 1 mmol to 10 mmol were conducted (Figure 2). The C-5 isomer was not found. Furthermore, NMR spectra, such as ^1^H-^1^H NOESY, and HMBC could prove that the structure of the desired product belongs to the C-4 isomer. The H_11_ had the chemical shift value of 4.56 ppm because it was adjacent to an oxygen atom. It had heteronuclear coupling with C_10_ and C_12_. Accordingly, C_6_ had heteronuclear coupling with H_10_ and H_11_ (see Supporting Information).

**Table 1:** Catalyst screening and optimization of the three-component reactions ^a^. 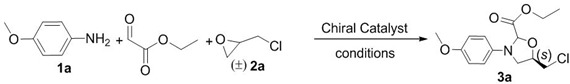

Entry	Solvent	Catal.	T (°C)	Yield (%) ^b^	d.r. ^c^	*ee* (%) ^d^
1	CH_2_Cl_2_	**4a**	18	5	2:1	10
2	CH_2_Cl_2_	**4b**	18	13	3:1	15
3	CH_2_Cl_2_	**4b**	−10	13	2:1	30
4 ^e^	CH_2_Cl_2_	**4c**/Ti(IV)	−40	13	3:1	41
5 ^e^	PhCH_3_	**4c**/Ti(IV)	−40	15	4:1	60
6 ^e^	PhCH_3_	**4c**/Ti(IV)	−40	50	4:1	61
7 ^f^	PhCH_3_	**4c**/Ti(IV)	−40	53	11:1	72
8 ^f^	PhCH_3_	**4d**/Ti(IV)	−40	36	11:1	72
9 ^f^	PhCH_3_	**4c**/Ti(IV)	−55	30	12:1	73
10 ^f^	PhCH_3_	**4c**/Ti(IV)	−70	15	12:1	73

^a^ **1a** (1.1 mmol) and ethyl glyoxalate (1.0 mmol) were stirred for 1 h in 1.5 mL solvent, then **2a** (0.2 mmol) and catalyst (0.1 mmol) were added, and the system was stirred for 4 days; ^b^ Yields of isolated products; ^c,d^ were determined by HPLC on a chiral column; ^e^ Catalysts were prepared by Ti(IV) and **4c** ligand in a 1:1 molar ratio and TFA was added into the reactions as a catalyst; ^f^ Catalysts were prepared by Ti(IV) and **4c**, **4d** ligands in a 1:2 molar ratio and TFA was added into the reactions as a catalyst.

The three-component reactions of anilines, ethyl glyoxalate, and epoxides were expanded under the optimized conditions (Table 2). A series of chiral 1,3-oxazolidine derivatives in moderate yields with good diastereoselectivities and enantioselectivities was provided, such as 4-chloroaniline, affording good *ee* and diastereoselectivity (Table 2, entries 2, 8, and 9). Product **3h** was obtained with a good enantioselectivity (Table 2, entry 8). Product **3i** was obtained with the highest *ee* (up to 90%) (Table 2, entry 9). From entries 1, 2, 3, and 11 in Table 2, we found that the *ee* changed with group R^1^. The electron-withdrawing group was negative for the reaction, such as entry 11 in Table 2. Therefore, substrates bearing electron-donating substituents were advantageous to the reaction. We speculated the reaction is a kinetic resolution as the yields are around 50%. Thus, the reaction of entry 10 (Table 2) also had been carried out as controlled experiment. Racemic (±)-epoxystyrene was used as substrate to react with ethyl glyoxalate and anisidine under the condition listed in Table 2. When the reaction was finished, unreacted epoxystyrene was subjected into a chiral column on the HPLC. To our delight, the epoxystyrene recollected from the reaction is no longer racemic, according to the HPLC analysis. Instead of the substrate racemic (±)-epoxystyrene, unreacted epoxystyrene with 68% *ee* was obtained. This means one enantiomer of the racemic epoxystyrene was consumed and the other was kept and the reaction is indeed a kinetic resolution (see Supporting Information).

To understand more about the reaction, effort also has been made to determine the absolute configuration of the products. Firstly, we figured out the retention time of (*R*)-epoxystyrene and (*S*)-epoxystyrene by detecting the racemic (±)-epoxystyrene and a standard (*R*)-epoxystyrene. Then, we reclaimed unreacted epoxystyrene from the reaction of entry 10 in Table 2, by checking the retention time. We found (*S*)-epoxystyrene was consumed and (*R*)-epoxystyrene not. This means that the absolute configuration of the chiral center from epoxides in the product is *S*-configuration [10]. (see Supporting Information).

**Figure 3:**
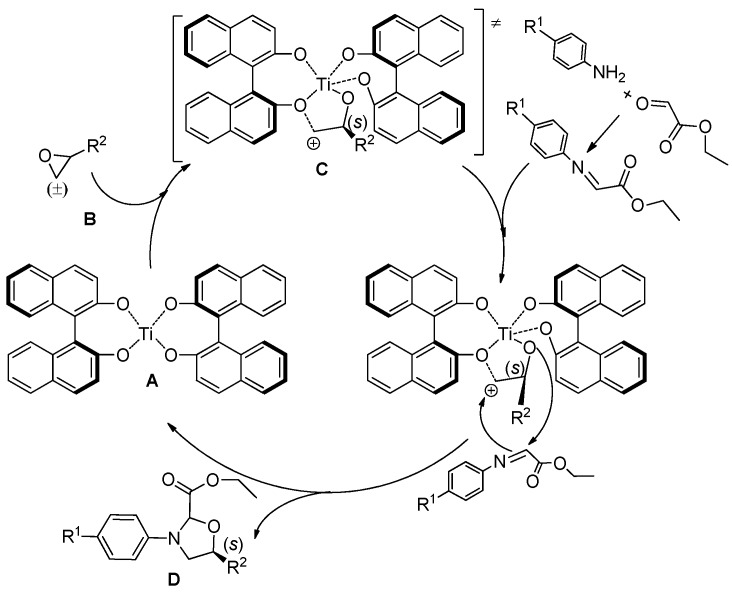
Proposed reaction mechanism.

On the basis of previous studies and the results of our experiments [13,42,43,44,45,46,47,48,49,50,51,52,53], a possible reaction mechanism of the present reaction is proposed. Firstly, (*S*)-BINOL reacted with Ti(O-*i*-Pr)_4_ for two hours to give a complex **A**. When racemic epoxide **B** was added, a transition state **C** was formed by the complexation of **A** and (*S*)-epoxide with the influence of TFA. Immediately, **C** reacted with the imine from aniline and ethyl glyoxalate to give the target product **D**, releasing out complexes **A** to participate the next cycle at the same time (Figure 3).

**Table 2:** Three-component reaction of anilines, ethyl glyoxalate, and epoxides ^a^. 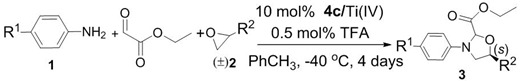

Entry	3	R^1^	R^2^	Yield (%) ^b^	d.r. ^c^	*ee* (%) ^d^
1	**3a**	CH_3_O	CH_2_Cl	52	12:1	43
2	**3b**	Cl	CH_2_Cl	50	10:1	69
3	**3c**	CH_3_CH_2_O	CH_2_Cl	47	10:1	39
4	**3d**	CH_3_O	CH_2_OCH(CH_3_)_2_	56	4:1	43
5	**3e**	CH_3_O	CH_2_O(CH_2_)_3_CH_3_	46	1.5:1	71
6	**3f**	CH_3_CH_2_O	CH_2_O(CH_2_)_3_CH_3_	53	3:1	34
7	**3g**	CH_3_CH_2_O	CH_2_OCH(CH_3_)_2_	53	3:1	72
8	**3h**	Cl	CH_2_O(CH_2_)_3_CH_3_	48	3:1	69
9	**3i**	Cl	CH_2_OCH(CH_3_)_2_	54	4:1	90
10	**3j**	CH_3_O	Ph	42	1.7:1	84.6
11	**3k**	NO_2_	CH_2_Cl	trace	―	―

^a^ Reactions were carried out under optimum conditions; ^b^ Yields of isolated products; ^c^determined by ^1^H-NMR; ^d^ determined by HPLC on a chiral column.

## 3. Experimental Section

### 3.1. General Procedure for the Synthesis of All 1,3-Oxazolidines

Ti(O-*i*-Pr)_4_ (0.05 mmol) and chiral binaphthalene ligand (**4c**, 0.10 mmol) were dissolved in 2.0 mL toluene, and the mixture was stirred for 2 h at room temperature, then aniline (1.1 mmol) and ethyl glyoxalate (1.0 mmol) were added into the mixture, and the result system was stirred for 30 min. Finally epoxide (1.2 mmol) and TFA (0.5 mol %) were added into the system and were stirred at −40 °C for four days. Then, the solvent was evaporated under vacuum. The residue was purified by silica gel column chromatography using 1:5 ethyl acetate/petroleum ether as eluent, giving a light yellow liquid. Enantiomeric excess (*ee*) were determined by HPLC analysis on a L-7420 (UV-VIS Detector with an L-7110 pump and a Chiralcel OD-H column). We determined the retention time of the product is based on significant changes in HPLC on a chiral column.

### 3.2. Characterization Data for All of the Compounds

*Ethyl 5-(chloromethyl)-3-(4-methoxyphenyl)oxazolidine-2-carboxylate* (**3a_1_**) Light yellow liquid; R*_f_* = 0.46 (1:5 ethyl acetate:petroleum ether); 48% yield (pure **3a_1_**). The enantiomeric excess (*ee*) was determined by HPLC on a Chiralcel OD-H column (*n*-hexane/isopropanol = 95/5, flow rate 0.5 mL/min, λ = 254 nm), t_R_ = 8.92 min (major), t_R_ = 10.397 min (minor), 43% *ee*; ^1^H-NMR (400 MHz, CDCl_3_) δ 6.82 (d, *J* = 6.8 Hz, 2H), 6.69 (d, *J* = 6.8 Hz, 2H), 5.42 (s, 1H), 4.58–4.55 (m, 1H), 4.18–4.16 (m, 2H), 3.84–3.80 (m, 1H), 3.78–3.72 (m, 5H), 3.56–3.52 (m, 1H), 1.23 (t, *J* = 7.2 Hz, 3H). ^13^C-NMR (100 MHz, CDCl_3_) δ 170.2, 153.9, 138.7, 116.0, 115.0, 89.7, 78.6, 61.6, 55.8, 51.5, 44.834, 14.2. HRMS (EI^+^) exact mass calculated for C_14_H_18_ClNO_4_ [M]^+^ requires *m*/*z* 299.0924, found *m*/*z* 299.0937.

*Ethyl 5-(chloromethyl)-3-(4-methoxyphenyl)oxazolidine-2-carboxylate* (**3a_2_**) Light yellow liquid; R*_f_* = 0.36 (1:5 ethyl acetate:petroleum ether); 4% yield (pure **3a_2_**). ^1^H-NMR (400 MHz, CDCl_3_) δ 6.84 (d, *J* = 6.8 Hz, 2H), 6.67 (d, *J* = 6.8 Hz, 2H), 5.46 (s, 1H), 4.92–4.88 (m, 1H), 4.22–4.16 (m, 2H), 3.78–3.69 (m, 5H), 3.65–3.60 (m, 1H), 3.43–3.39 (m, 1H), 1.25 (t, *J* = 7.2 Hz, 3H). ^13^C-NMR (100 MHz, CDCl_3_) δ 169.3, 152.6, 138.3, 114.5, 114.0, 88.3, 77.6, 60.9, 55.2, 49.6, 44.3, 13.7. HRMS (EI^+^) exact mass calculated for C_14_H_18_ClNO_4_ [M]^+^ requires *m*/*z* 299.0924, found *m*/*z* 299.0937.

*Ethyl 5-(chloromethyl)-3-(4-chlorophenyl)oxazolidine-2-carboxylate* (**3b_1_**) Light yellow liquid; R*_f_* = 0.57 (1:5 ethyl acetate:petroleum ether); 46% yield (pure **3b_1_**). The enantiomeric excess (*ee*) was determined by HPLC on a Chiralcel OD-H column (*n*-hexane/isopropanol = 95/5, flow rate 1 mL/min, λ = 254 nm), t_R_ = 12.13 min (major), t_R_ = 8.09 min (minor), 61.1% *ee* (minor); ^1^H-NMR (400 MHz, CDCl_3_) δ 7.19 (d, *J* = 6.8 Hz, 2H), 6.59 (d, *J* = 6.8 Hz, 2H), 5.45 (s, 1H), 4.63–4.59 (m, 1H), 4.19–4.17 (m, 2H), 3.83–3.79 (m, 2H), 3.76–3.71 (m, 1H), 3.56–3.52 (m, 1H), 1.24 (t, *J* = 7.2 Hz, 3H). ^13^C-NMR (100 MHz, CDCl_3_) δ 169.8, 142.695, 129.4, 124.5, 115.0, 88.5, 78.8, 61.9, 50.5, 44.7, 14.2. HRMS (EI^+^) exact mass calculated for C_13_H_15_Cl_2_NO_3_ [M]^+^ requires *m*/*z* 303.0429, found *m*/*z* 303.0424.

*Ethyl 5-(chloromethyl)-3-(4-chlorophenyl)oxazolidine-2-carboxylate* (**3b_2_**) Light yellow liquid; R*_f_* = 0.46 (1:5 ethyl acetate:petroleum ether); 4% yield (pure **3b_2_**). ^1^H-NMR (400 MHz, CDCl_3_) δ 7.21 (d, *J* = 6.8 Hz, 2H), 6.62 (d, *J* = 6.8 Hz, 2H), 5.48 (s, 1H), 4.97–4.94 (m, 1H), 4.23–4.21 (m, 2H), 3.79–3.72 (m, 2H), 3.68–3.64 (m, 1H), 3.45–3.42 (m, 1H), 1.28 (t, *J* = 7.2 Hz, 3H). ^13^C-NMR (100 MHz, CDCl_3_) δ 170.8, 144.2, 130.7, 125.3, 115.6, 89.4, 79.3, 63.1, 50.9, 45.8, 15.5. HRMS (EI^+^) exact mass calculated for C_13_H_15_Cl_2_NO_3_ [M]^+^ requires *m*/*z* 303.0429, found *m*/*z* 303.0424.

*Ethyl 5-(chloromethyl)-3-(4-ethoxyphenyl)oxazolidine-2-carboxylate* (**3c_1_**) Light yellow liquid; R*_f_* = 0.48 (1:5 ethyl acetate:petroleum ether); 47% yield (**3c_1_**). The enantiomeric excess (*ee*) was determined by HPLC on a Chiralcel OD-H column (*n*-hexane/isopropanol = 95/5, flow rate 0.5 mL/min, λ = 254 nm), t_R_ = 7.79 min (major), t_R_ = 8.57 min (minor), 39% *ee*; ^1^H-NMR (400 MHz, CDCl_3_) δ 6.83 (d, *J* = 6.8 Hz, 2H), 6.65 (d, *J* = 6.8 Hz, 2H), 5.45 (s, 1H), 4.93–4.87 (m, 1H), 4.20–4.16 (m, 2H), 4.00–3.94 (m, 2H), 3.79–3.69 (m, 2H), 3.64–3.61 (m, 1H), 3.43–3.39 (m, 1H), 1.38 (t, *J* = 7.2 Hz, 3H), 1.24 (t, *J* = 7.2 Hz, 3H). ^13^C-NMR (100 MHz, CDCl_3_) δ 169.9, 152.5, 138.8, 115.9, 114.6, 88.9, 78.1, 64.2, 61.5, 50.2, 44.7, 15.1, 14.3. HRMS (EI^+^) exact mass calculated for C_15_H_20_ClNO_4_ [M + H]^+^ requires *m*/*z* 314.1154, found *m*/*z* 314.1155.

*Ethyl 5-(isopropoxymethyl)-3-(4-methoxyphenyl)oxazolidine-2-carbo-xylate* (**3d**) (**diastereoisomers**): Light yellow liquid; R*_f_* = 0.33 (1:5 ethyl acetate:petroleum ether); 56% yield. The enantiomeric excess (*ee*) was determined by HPLC on a Chiralcel OD-H column (*n*-hexane/isopropanol = 95/5, flow rate 0.5 mL/min, λ = 254 nm), t_R_ = 18.40 min (major), t_R_ = 19.12 min (minor), 41.5% *ee* (minor); ^1^H-NMR (400 MHz, CDCl_3_) δ 6.85–6.81 (m, 2H[[2Hʹ]), 6.66–6.63 (m, 2H[[2Hʹ]), 5.39 (s, 1H) and 5.42, (s, 1Hʹ)], 4.84–4.80 (m, 1H[[1Hʹ]), 4.21–4.13 (m, 2H[[2Hʹ]), 3.75 (s, 3H), 3.68–3.60 (m, 2H), 3.59–3.55 (m, 2H), 3.43–3.25 (m, 1H[[1Hʹ]), 1.25 (t, *J* = 7.2 Hz, 3H), 1.21–1.15 (m, 6H). ^13^C-NMR (100 MHz, CDCl_3_) δ 170.2 153.0, 139.3 115.0 and [[115.4, (1Cʹ)], 114.3 88.7 and [[88.8, (1Cʹ)], 78.0, 72.5, 69. and [[69.8 (1Cʹ)], 61.3, 55.8, 49.6 and [[49.8, (1Cʹ)], 22.1, 14.2 HRMS (EI^+^) exact mass calculated for C_17_H_25_NO_5_ [M]^+^ requires *m*/*z* 323.1733, found *m*/*z* 323.1743.

*Ethyl 5-(butoxymethyl)-3-(4-methoxyphenyl)oxazolidine-2-carboxylate* (**3e**) (**diastereoisomers**): Light yellow liquid; R*_f_* = 0.44 (1:5 ethyl acetate:petroleum ether); 46% yield. The enantiomeric excess (*ee*) was determined by HPLC on a Chiralcel OD-H column (*n*-hexane/isopropanol = 95/5, flow rate 0.5 mL/min, λ = 254 nm), t_R_ = 19.49 min (major), t_R_ = 22.00 min (minor), 71% *ee*; ^1^H-NMR (400 MHz, CDCl_3_) δ 6.876.83 (m, 2H[[2Hʹ]), 6.68–6.66 (m, 2H[[2Hʹ]), 5.41 (s, 1H) and 5.44 (s, 1Hʹ), 4.874.50 (m, 1H[[1Hʹ]), 4.224.15 (m, 2H[[2Hʹ]), 3.77 (s, 3H), 3.68–3.62 (m, 2H[[2Hʹ]), 3.543.50 m, 2H[[2Hʹ]), 3.43–3.27 (m, 2H[[2Hʹ]), 1.68–1.57 (m, 4H), 1.42–1.35 (m, 3H), 1.29–1.21 (m, 3H[[3Hʹ]). ^13^C-NMR (100 MHz, CDCl_3_) δ 169.7, 152.3, 138.9, 114.4 and [[115.0, (1Cʹ)], 113.8, 88.1 (1C) and [[88.6, (1Cʹ)], 77.2, 70.9, 60.7, 55.2, 50.4 and [[49.8, (1Cʹ)], 48.9, 43.780, 31.3, 18.7, 13.4. HRMS (EI^+^) exact mass calculated for C_18_H_27_NO_5_ [M]^+^ requires *m*/*z* 337.1889, found *m*/*z* 337.1886.

*Ethyl 5-(butoxymethyl)-3-(4-ethoxyphenyl)oxazolidine-2-carboxylate* (**3f**) (**diastereoisomers**): Light yellow liquid; R*_f_* = 0.43 (1:5 ethyl acetate:petroleum ether); 53% yield. The enantiomeric excess (*ee*) was determined by HPLC on a Chiralcel OD-H column (*n*-hexane/isopropanol = 95/5, flow rate 1 mL/min, λ = 254 nm), t_R_ = 7.94 min (major), t_R_ = 8.47 min (minor), 34.5% *ee* (minor); ^1^H-NMR (400 MHz, CDCl_3_) δ 6.84–6.80 (m, 2H[[2Hʹ]), 6.64–6.61 (m, 2H[[2Hʹ]), 5.38 (s, 1H) and [[5.41, (s, 1Hʹ)], 4.84–4.45 (m, 1H[[1Hʹ]), 4.21–4.16 (m, 2H[[2Hʹ]), 3.99–3.93 (m, 2H), 3.68–3.60 (m, 2H[[2Hʹ]), 3.51–3.47 (m, 2H), 3.40–3.25 (m, 2H[[2Hʹ]), 1.59–1.54 (m, 4H), 1.40–1.33 (m, 6H), 1.27–1.1837 (m, 3H[[3Hʹ]). ^13^C-NMR (100 MHz, CDCl_3_) δ 170.37, 152.2, 139.4, 115.8, 114.2, 88.7 and [[88.7, (1Cʹ)], 77.8, 71.5, 64.13, 61.3, 51.0 and [[50.4, (1Cʹ)], 49.4, 44.4, 31.7, 19.3, 15.0, 14.0. HRMS (ESI^+^) exact mass calculated for [M + Na]^+^ requires *m*/*z* 374.1944, found *m*/*z* 374.1935.

*Ethyl 3-(4-ethoxyphenyl)-5-(isopropoxymethyl)oxazolidine-2-carboxylate* (**3g**) (**diastereoisomers**): Light yellow liquid; R*_f_* = 0.43 (1:5 ethyl acetate:petroleum ether); 53% yield. The enantiomeric excess (*ee*) was determined by HPLC on a Chiralcel OD-H column (*n*-hexane/isopropanol = 95/5, flow rate 1 mL/min, λ = 254 nm), t_R_ = 6.59 min (major), t_R_ = 7.90 min (minor), 72% *dr*; ^1^H-NMR (400 MHz, CDCl_3_) δ 6.85–6.81 (m, 2H[[2Hʹ]), 6.66–6.62 (m, 2H[[2Hʹ]), 5.40 (s, 1H) and [[5.42, (s, 1Hʹ)], 4.82–4.81 (m, 1H[[1Hʹ]), 4.22–4.14 (m, 2H[[2Hʹ]), 4.00–3.94 (m, 2H[[2Hʹ]), 3.69–3.65 (m, 2H[[2Hʹ]), 3.64–3.55 (m, 2H[[2Hʹ]), 3.29–3.26 (m, 1H[[1Hʹ]), 1.38 (t, *J* = 7.2 Hz, 3H), 1.27–1.23 (m, 3H), 1.20–1.16 (m, 6H). ^13^C-NMR (100 MHz, CDCl_3_) δ 169.8, 151.6, 138.9, 115.3, 113.7, 88.1 and [[88.2, (1Cʹ)], 77.4, 71.9, 68.4 and [[69.2, (1Cʹ)], 63.6, 60.7, 49.0 and [[50.0, (1Cʹ)], 21.5, 14.5, 13.6. HRMS (EI^+^) exact mass calculated for C_18_H_27_NO_5_ [M]^+^ requires *m*/*z* 337.1889, found *m*/*z* 337.1881.

*Ethyl 5-(butoxymethyl)-3-(4-chlorophenyl)oxazolidine-2-carboxylate* (**3h**) (**diastereoisomers**): Light yellow liquid; R*_f_* = 0.57 (1:5 ethyl acetate:petroleum ether); 48% yield. The enantiomeric excess (*ee*) was determined by HPLC on a Chiralcel OD-H column (*n*-hexane/isopropanol = 95/5, flow rate 1 mL/min, λ = 254 nm), t_R_ = 4.74 min (major), t_R_ = 6.18 min (minor), 69*.*4% *ee* (major); ^1^H-NMR (400 MHz, CDCl_3_) δ 7.20–7.17 (m, 2H[[2Hʹ]), 6.59–6.54 (m, 2H[[2Hʹ]), 5.40 (s, 1H) and [[5.44, (s, 1Hʹ)], 4.87–4.53 (m, 1H[[1Hʹ]), 4.23–4.15 (m, 2H[[2Hʹ]), 3.67–3.61 (m, 2H[[2Hʹ]), 3.52–3.47 (m,2H), 3.41–3.28 (m, 2H[[2Hʹ]), 1.60–1.53 (m, 4H), 1.40–1.31 (m, 3H), 1.28–1.20 (m, 3H[[3Hʹ]). ^13^C-NMR (100 MHz, CDCl_3_) δ 169.8, 143.3, 129.2, 123.5 and [[123.9, (1Cʹ)], 114.1 and [[114.7, (1Cʹ)], 87.1 and [[88.0, (1Cʹ)], 77.8, 71.8, 71.2, 61.6, 48.8 and [[49.5, (1Cʹ)], 31.7, 19.3, 14.2, 14.0. HRMS (EI^+^) exact mass calculated for C_17_H_24_ClNO_4_ [M]^+^ requires *m*/*z* 341.1394, found *m*/*z* 341.1397.

*Ethyl 3-(4-chlorophenyl)-5-(isopropoxymethyl)oxazolidine-2-carboxylate* (**3i**) (**diastereoisomers**): Light yellow liquid; R*_f_* = 0.52 (1:5 ethyl acetate:petroleum ether); 54% yield. The enantiomeric excess (*ee*) was determined by HPLC on a Chiralcel OD-H column (*n*-hexane/isopropanol = 95/5, flow rate 1 mL/min, λ = 254 nm), t_R_ = 6.73 min (major), t_R_ = 7.82 min (minor), 90% *ee*; ^1^H-NMR (400 MHz, CDCl_3_) δ 7.20–7.17 (m, 2H[[2Hʹ]), 6.59–6.54 (m, 2H[[2Hʹ]), 5.40 (s, 1H) and [[5.43, (s, 1Hʹ)], 4.86–4.82 (m, 1H[[1Hʹ]), 4.22–4.15 (m, 2H[[2Hʹ]), 3.75–3.64 (m, 2H[[2Hʹ]), 3.63–3.57 (m, 2H[[2Hʹ]), 3.31–3.27 (m, 1H[[1Hʹ]), 1.31–1.24 (m, 3H[[3Hʹ]), 1.22–1.16 (m, 6H[[6Hʹ]), ^13^C-NMR (100 MHz, CDCl_3_) δ 169.8, 143.4, 129.2, 123.5, 114.1 and [[114.7, (1Cʹ)], 87.9 and [[88.0, (1Cʹ)], 78.0, 72.6, 68.7 and [[69.6, (1Cʹ)], 61.6, 49.0 and [[49.7, (1Cʹ)], 22.1, 14.2. HRMS (ESI^+^) exact mass calculated for [M + Na]^+^ requires *m*/*z* 350.1130, found *m*/*z* 350.1131.

*Ethyl 3-(4-methoxyphenyl)-5-phenyloxazolidine-2-carboxy-late* (**3j**) (**diastereoisomers**): Light yellow solid; M.P.: 53–56 °C; R*_f_* = 0.50 (1:5 ethyl acetate:petroleum ether); 42% yield. The enantiomeric excess (*ee*) was determined by HPLC on a Chiralcel OD-H column (*n*-hexane/isopropanol = 99/1, flow rate 0.30 mL/min, λ = 254 nm), t_R_ = 28.61 min (major), t_R_ = 30.667 min (minor), 84.6% *ee*; ^1^H-NMR (400 MHz, CDCl_3_) δ 7.50–7.48 (d, 2H), 7.33–7.32 (m, 3H), 6.79–6.73 (m, 2H), 6.71–6.66 (m, 2H), 5.50 (s, 1H) 4.36–4.27 (m, 2H), 4.24–4.16 (m, 2H), 4.05–4.02 (m, 1H), 3.72 (s, 3H) 1.40–1.37 (m, 3H), ^13^C-NMR (100 MHz, CDCl_3_) δ 170.70, 153.06,139.84, 139.19, 128.91, 127.83, 126.52, 114.88, 114.65, 90.85, 75.37, 63.75, 61.53, 55.59, 14.21. HRMS (EI^+^) exact mass calculated for C_19_H_21_NO_4_ [M]^+^ requires *m*/*z* 327.1471, found *m*/*z* 327.1471.

## 4. Conclusions

In conclusion, we disclosed an efficient asymmetric three-component reaction of anilines, ethyl glyoxalates, and epoxides, yielding 1,3-oxazolidine derivatives with high diastereo and enantioselectivities (up to 20:1 d.r., 90% *ee*) by cascade process. This reaction provides a convenient method to synthesize multi-substituted 1,3-oxazolidine compounds of high optical purity. The plausible mechanism was suggested and a kinetic resolution process was confirmed. All of the products in this paper were well-characterized and the absolute configuration of the chiral center from epoxides was determined. We believe this study will enrich the methodologies for the synthesis of five-membered oxacycles and natural products. Further studies are underway and other synthetic applications will be reported in a due time.

## Supplementary Materials

Supplementary materials can be accessed at: http://www.mdpi.com/1420-3049/20/09/17208/s1.
