# Supplementary material for: Asymmetric Synthesis of 1,3-Oxazolidine Derivatives with Multi-Component Reaction and Research of Kinetic Resolution
Source: Molecules. 2015 Sep 18;20(9):17208–20. doi: 10.3390/molecules200917208 (PMC6332461; doi:10.3390/molecules200917208)
Supplement: Supplementary file 1 [file molecules-20-17208-s001.pdf]

Supporting Information

Table of Contents:

1. General……………………………………………………….....................................….........S1
2. Typical procedure for the asymmetric reaction of anilines, ethyl glyoxalate and racemic epoxides…….. ....S1
3. Characterization data of 1,3-oxazolidine derivatives………...S2–S6
4. ^{1}H- and ^{13}C-NMR spectra and HPLC analysis of products…...S7–S50
5. HPLC analysis for epoxystyrene………………………………..S50–S52
6. HMBC spectrum of product **3a**……………………...S53
7. NOESY spectrum of product **3a**..S54

1. General

Unless otherwise noted, materials were used as commercial suppliers. All solvents were purified by standard method. Flash column chromatography was performed using 200–300 mesh silica gel.

Reaction progress was followed by TLC analysis at 254 nm. NMR spectroscopy was performed on 400 MHz spectrometer operating at 400 MHz (^{1}H-NMR) and 100 MHz (^{13}C-NMR). TMS was used as an internal standard and $CDCl_3$ was used as the solvent. ^{1}H-NMR data were reported as follows: chemical shifts in ppm downfield from tetramethylsilane, multiplicity (s = singlet, d = doublet, t = triplet, q = quartet, m = multiplet and br = broad), coupling constant = *J*. IR spectra were recorded by using KBr optics. All the reagents are used directly from commercial and without further purification.

2. Typical Procedure for the Asymmetric Reaction of Anilines, Ethyl Glyoxalate and Racemic Epoxides

Ti(O-*i*-Pr)$_4$ (0.05 mmol) and chiral binaphthalene ligand (**4c**, 0.10 mmol) were dissolved in 2.0 mL toluene, and the mixture was stirred for 2 h at room temperature, then aniline (1.1 mmol) and ethyl glyoxalate (1 mmol) were added into the mixture, and the result system was stirred for 30 mins. Finally epoxide (0.12 mmol) and TFA (0.5 mol %) were added into the system and were stirred at −40 °C for 4 days. Then the solvent was evaporated under vacuum. The residue was purified by silica gel column chromatography using 1:5 ethyl acetate/petroleum ether as eluent, giving a light yellow liquid. Enantiomeric excess (*ee*) were determined by HPLC analysis on a L-7420 (UV-VIS Detector with an L-7110 pump and a Chiralcel OD-H column.

3. Characterization Data of 1,3-oxazolidine Derivatives

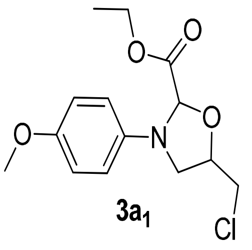

3a₁

Ethyl 5-(chloromethyl)-3-(4-methoxyphenyl)oxazolidine-2-carboxylate (**3a₁**). Light yellow liquid; $R_f = 0.46$ (1:5 ethyl acetate:petroleum ether); 48% yield (pure **3a₁**). The enantiomeric excess (*ee*) was determined by HPLC on a Chiralcel OD-H column (*n*-hexane/isopropanol = 95/5, flow rate 0.5 mL/min, λ = 254 nm), t_R = 8.92 min (major), t_R = 10.397 min (minor), 43% *ee*; ^{1}H-NMR (400 MHz, $CDCl_3$) δ 6.82 (d, *J* = 6.8 Hz, 2H), 6.69 (d, *J* = 6.8 Hz, 2H), 5.42 (s, 1H), 4.58–4.55 (m, 1H), 4.18–4.16 (m, 2H), 3.84–3.80 (m, 1H), 3.78–3.72 (m, 5H), 3.56–3.52 (m, 1H), 1.23 (t, *J* = 7.2 Hz, 3H). ^{13}C-NMR (100 MHz, $CDCl_3$) δ 170.2, 153.9, 138.7, 116.0, 115.0 89.7, 78.6, 61.6, 55.8, 51.5, 44.834, 14.2. HRMS (EI^+) exact mass calculated for $C_{14}H_{18}ClNO_4$ $[M]^+$ requires *m*/*z* 299.0924, found *m*/*z* 299.0937.

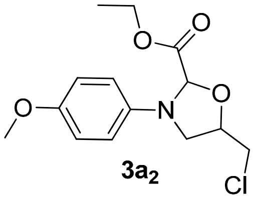

3a₂

Ethyl 5-(chloromethyl)-3-(4-methoxyphenyl)oxazolidine-2-carboxylate (**3a₂**). Light yellow liquid; $R_f = 0.36$ (1:5 ethyl acetate:petroleum ether); 4% yield (pure **3a₂**). ^{1}H-NMR (400 MHz, $CDCl_3$) δ 6.84 (d, *J* = 6.8 Hz, 2H), 6.67 (d, *J* = 6.8 Hz, 2H), 5.46 (s, 1H), 4.92–4.88 (m, 1H), 4.22–4.16 (m, 2H), 3.78–3.69 (m, 5H), 3.65–3.60 (m, 1H), 3.43–3.39 (m, 1H), 1.25 (t, *J* = 7.2 Hz, 3H). ^{13}C-NMR (100 MHz, $CDCl_3$) δ 169.3, 152.6, 138.3, 114.5, 114.0, 88.3, 77.6, 60.9, 55.2, 49.6, 44.3, 13.7. HRMS (EI^+) exact mass calculated for $C_{14}H_{18}ClNO_4$ $[M]^+$ requires *m*/*z* 299.0924, found *m*/*z* 299.0937.

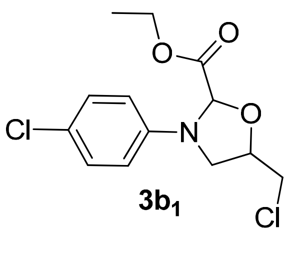

3b₁

Ethyl 5-(chloromethyl)-3-(4-chlorophenyl)oxazolidine-2-carboxylate (**3b₁**). Light yellow liquid; $R_f = 0.57$ (1:5 ethyl acetate:petroleum ether); 46% yield (pure **3b₁**). The enantiomeric excess (*ee*) was determined by HPLC on a Chiralcel OD-H column (*n*-hexane/isopropanol = 95/5, flow rate 1 mL/min, λ = 254 nm), t_R = 12.13 min (major), t_R = 8.09 min (minor), 61.1% *ee* (minor); ^{1}H-NMR (400 MHz, $CDCl_3$) δ 7.19 (d, *J* = 6.8 Hz, 2H), 6.59 (d, *J* = 6.8 Hz, 2H), 5.45 (s, 1H), 4.63–4.59 (m, 1H), 4.19–4.17(m, 2H), 3.83–3.79 (m, 2H), 3.76–3.71 (m, 1H), 3.56–3.52 (m, 1H), 1.24 (t, *J* = 7.2 Hz, 3H). ^{13}C-NMR (100 MHz, $CDCl_3$) δ 169.8, 142.695, 129.4, 124.5, 115.0, 88.5, 78.8, 61.9, 50.5, 44.7, 14.2. HRMS (EI^+) exact mass calculated for $C_{13}H_{15}Cl_2NO_3$ $[M]^+$ requires *m*/*z* 303.0429, found *m*/*z* 303.0424.

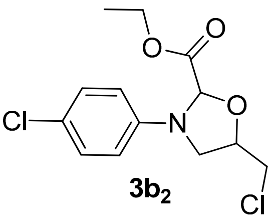

3b$_2$

Ethyl 5-(chloromethyl)-3-(4-chlorophenyl)oxazolidine-2-carboxylate (**3b$_2$**). Light yellow liquid; R_f = 0.46 (1:5 ethyl acetate:petroleum ether); 4% yield (pure **3b$_2$**). ^{1}H-NMR (400 MHz, $CDCl_3$) δ 7.21 (d, J = 6.8 Hz, 2H), 6.62 (d, J = 6.8 Hz, 2H), 5.48 (s, 1H), 4.97–4.94 (m, 1H), 4.23–4.21 (m, 2H), 3.79–3.72 (m, 2H), 3.68–3.64 (m, 1H), 3.45–3.42 (m, 1H), 1.28 (t, J = 7.2 Hz, 3H). ^{13}C-NMR (100 MHz, $CDCl_3$) δ 170.8, 144.2, 130.7, 125.3, 115.6, 89.4, 79.3, 63.1, 50.9, 45.8, 15.5. HRMS (EI^+) exact mass calculated for $C_{13}H_{15}Cl_2NO_3$ $[M]^+$ requires *m*/*z* 303.0429, found *m*/*z* 303.0424.

Ethyl 5-(chloromethyl)-3-(4-ethoxyphenyl)oxazolidine-2-carboxylate (**3c$_1$**)

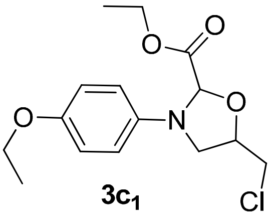

3c$_1$

Light yellow liquid; R_f = 0.48 (1:5 ethyl acetate:petroleum ether); 47% yield (**3c$_1$**). The enantiomeric excess (*ee*) was determined by HPLC on a Chiralcel OD-H column (*n*-hexane/isopropanol = 95/5, flow rate 0.5 mL / min, λ = 254 nm), t_R = 7.79 min (major), t_R = 8.57 min (minor), 39% *ee*; ^{1}H-NMR (400 MHz, $CDCl_3$) δ 6.83 (d, J = 6.8 Hz, 2H), 6.65 (d, J = 6.8 Hz, 2H), 5.45 (s, 1H), 4.93–4.87 (m, 1H), 4.20–4.16 (m, 2H), 4.00–3.94 (m, 2H), 3.79–3.69 (m, 2H), 3.64–3.61 (m, 1H), 3.43–3.39 (m, 1H), 1.38 (t, J = 7.2 Hz, 3H), 1.24 (t, J = 7.2 Hz, 3H). ^{13}C-NMR (100 MHz, $CDCl_3$) δ 169.9, 152.5, 138.8, 115.9, 114.6, 88.9, 78.1, 64.2, 61.5, 50.2, 44.7, 15.1, 14.3. HRMS (EI^+) exact mass calculated for $C_{15}H_{20}ClNO_4$ $[M + H]^+$ requires *m*/*z* 314.1154, found *m*/*z* 314.1155.

Above products of **3a**, **3b** and **3c** as pure chiral compounds could be successfully separated as single isomers, but below products **3d**, **3e**, **3f**, **3g**, **3h**, **3i**, **3j** couldn't be separated as single isomers from their stereoisomers.

Ethyl 5-(isopropoxymethyl)-3-(4-methoxyphenyl)oxazolidine-2-carbo- xylate (**3d**) (diasteroisomers)

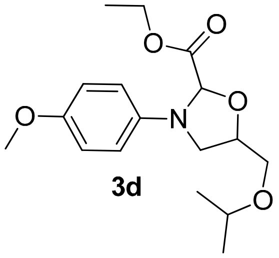

3d

Light yellow liquid; R_f = 0.33 (1:5 ethyl acetate:petroleum ether); 56% yield. The enantiomeric excess (*ee*) was determined by HPLC on a Chiralcel OD-H column (*n*-hexane/isopropanol = 95/5, flow

rate 0.5 mL/min, λ = 254 nm), t_R = 18.40 min (major), t_R = 19.12 min (minor), 41.5% *ee* (minor); ^{1}H-NMR (400 MHz, $CDCl_3$) δ 6.85–6.81 (m, 2H[2H′]), 6.66–6.63 (m, 2H[2H′]), 5.39 (s, 1H) and 5.42, (s, 1H′)], 4.84–4.80 (m, 1H[1H′]), 4.21–4.13 (m, 2H[2H′]), 3.75 (s, 3H), 3.68–3.60 (m, 2H), 3.59–3.55 (m, 2H), 3.43–3.25 (m, 1H[1H′]), 1.25 (t, *J* = 7.2 Hz, 3H), 1.21–1.15 (m, 6H). ^{13}C-NMR (100 MHz, $CDCl_3$) δ 170.2 153.0, 139.3 115.0 and [115.4, (1C′)], 114.3 88.7and [88.8, (1C′)], 78.0, 72.5, 69.and [69.8 (1C′)], 61.3, 55.8, 49.6 and [49.8, (1C′)], 22.1, 14.2 HRMS (EI$^+$) exact mass calculated for $C_{17}H_{25}NO_5$ [M]$^+$ requires *m*/*z* 323.1733, found *m*/*z* 323.1743.

Ethyl 5-(butoxymethyl)-3-(4-methoxyphenyl)oxazolidine-2-carboxylate (**3e**) (diastereoisomers)

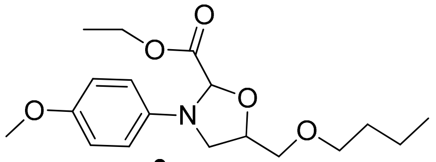

3e

Light yellow liquid; R_f = 0.44(1:5 ethyl acetate:petroleum ether); 46% yield. The enantiomeric excess (*ee*) was determined by HPLC on a Chiralcel OD-H column (*n*-hexane/isopropanol = 95/5, flow rate 0.5 mL/min, λ = 254 nm), t_R = 19.49min (major), t_R = 22.00 min (minor), 71% *ee*; ^{1}H-NMR (400 MHz, $CDCl_3$) δ 6.876.83(m, 2H[2H′]), 6.68–6.66 (m, 2H[2H′]), 5.41 (s, 1H) and 5.44 (s, 1H′), 4.874.50 (m, 1H[1H′]), 4.224.15 (m, 2H[2H′]), 3.77 (s, 3H), 3.68–3.62 (m, 2H[2H′]), 3.543.50 (m, 2H[2H′]), 3.43–3.27 (m, 2H[2H′]), 1.68–1.57 (m, 4H), 1.42–1.35 (m, 3H), 1.29–1.21 (m, 3H[3H′]). ^{13}C-NMR (100 MHz, $CDCl_3$) δ 169.7, 152.3, 138.9, 114.4 and [115.0, (1C′)], 113.8, 88.1 (1C) and [88.6, (1C′)], 77.2, 70.9, 60.7, 55.2, 50.4 and [49.8, (1C′)], 48.9, 43.780, 31.3, 18.7, 13.4. HRMS (EI$^+$) exact mass calculated for $C_{18}H_{27}NO_5$ [M]$^+$ requires *m*/*z* 337.1889, found *m*/*z* 337.1886.

Ethyl 5-(butoxymethyl)-3-(4-ethoxyphenyl)oxazolidine-2-carboxylate (**3f)** (diastereoisomers)

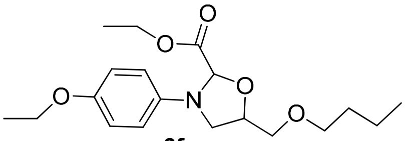

3f

Light yellow liquid; R_f = 0.43 (1:5 ethyl acetate:petroleum ether); 53% yield. The enantiomeric excess (*ee*) was determined by HPLC on a Chiralcel OD-H column (*n*-hexane/isopropanol = 95/5, flow rate 1 mL/min, λ = 254 nm), t_R = 7.94 min (major), t_R = 8.47 min (minor), 34.5% *ee* (minor); ^{1}H-NMR (400 MHz, $CDCl_3$) δ 6.84–6.80 (m, 2H[2H′]), 6.64–6.61 (m, 2H[2H′]), 5.38 (s, 1H) and [5.41, (s, 1H′)], 4.84–4.45 (m, 1H[1H′]), 4.21–4.16 (m, 2H[2H′]), 3.99–3.93 (m, 2H), 3.68–3.60 (m, 2H[2H′]), 3.51–3.47 (m, 2H), 3.40–3.25 (m, 2H[2H′]), 1.59–1.54 (m, 4H), 1.40–1.33 (m, 6H), 1.27–1.1837 (m, 3H[3H′]). ^{13}C-NMR (100 MHz, $CDCl_3$) δ 170.37, 152.2, 139.4, 115.8, 114.2, 88.7 and [88.7, (1C′)], 77.8, 71.5, 64.13, 61.3, 51.0 and [50.4, (1C′)], 49.4, 44.4, 31.7, 19.3, 15.0, 14.0. HRMS (ESI$^+$) exact mass calculated for [M + Na]$^+$ requires *m*/*z* 374.1944, found *m*/*z* 374.1935.

Ethyl 3-(4-ethoxyphenyl)-5-(isopropoxymethyl)oxazolidine-2-carboxylate (**3g**) (diastereoisomers)

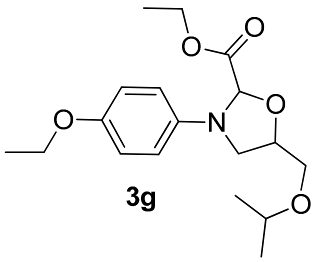

3g

Light yellow liquid; R_f = 0.43 (1:5 ethyl acetate:petroleum ether); 53% yield. The enantiomeric excess (*ee*) was determined by HPLC on a Chiralcel OD-H column (*n*-hexane/isopropanol = 95/5, flow rate 1 mL/min, λ = 254 nm), t_R = 6.59 min (major), t_R = 7.90 min (minor), 72% *dr*; ^{1}H-NMR (400 MHz, $CDCl_3$) δ 6.85–6.81 (m, 2H[2H′]), 6.66–6.62 (m, 2H[2H′]), 5.40 (s, 1H) and [5.42, (s, 1H′)], 4.82–4.81 (m, 1H[1H′]), 4.22–4.14 (m, 2H[2H′]), 4.00–3.94 (m, 2H[2H′]), 3.69–3.65 (m, 2H[2H′]), 3.64–3.55 (m, 2H[2H′]), 3.29–3.26 (m, 1H[1H′]), 1.38 (t, *J* = 7.2 Hz, 3H), 1.27–1.23 (m, 3H), 1.20–1.16 (m, 6H). ^{13}C-NMR (100 MHz, $CDCl_3$) δ 169.8, 151.6, 138.9, 115.3, 113.7, 88.1 and [88.2, (1C′)], 77.4, 71.9, 68.4 and [69.2, (1C′)], 63.6, 60.7, 49.0 and [50.0, (1C′)], 21.5, 14.5, 13.6. HRMS (EI$^+$) exact mass calculated for $C_{18}H_{27}NO_5$ $[M]^+$ requires *m*/*z* 337.1889, found *m*/*z* 337.1881.

Ethyl 5-(butoxymethyl)-3-(4-chlorophenyl)oxazolidine-2-carboxylate (**3h**) (diastereoisomers)

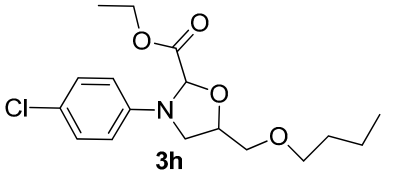

3h

Light yellow liquid; R_f = 0.57 (1:5 ethyl acetate:petroleum ether); 48% yield. The enantiomeric excess (*ee*) was determined by HPLC on a Chiralcel OD-H column (*n*-hexane / isopropanol = 95/5, flow rate 1 mL/min, λ = 254 nm), t_R = 4.74 min (major), t_R = 6.18 min (minor), 69.4% *ee* (major); ^{1}H-NMR (400 MHz, $CDCl_3$) δ 7.20–7.17 (m, 2H[2H′]), 6.59–6.54 (m, 2H[2H′]), 5.40 (s, 1H) and [5.44, (s, 1H′)], 4.87–4.53 (m, 1H[1H′]), 4.23–4.15 (m, 2H[2H′]), 3.67–3.61 (m, 2H[2H′]), 3.52–3.47 (m,2H), 3.41–3.28 (m, 2H[2H′]), 1.60–1.53 (m, 4H), 1.40–1.31 (m, 3H), 1.28–1.20 (m, 3H[3H′]). ^{13}C-NMR (100 MHz, $CDCl_3$) δ 169.8, 143.3, 129.2, 123.5 and [123.9, (1C′)], 114.1 and [114.7, (1C′)], 87.1 and [88.0, (1C′)], 77.8, 71.8, 71.2, 61.6, 48.8 and [49.5, (1C′)], 31.7, 19.3, 14.2, 14.0. HRMS (EI$^+$) exact mass calculated for $C_{17}H_{24}ClNO_4$ $[M]^+$ requires *m*/*z* 341.1394, found *m*/*z* 341.1397.

Ethyl 3-(4-chlorophenyl)-5-(isopropoxymethyl)oxazolidine-2-carboxylate (**3i**) (diastereoisomers)

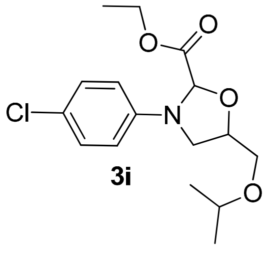

3i

Light yellow liquid; R_f = 0.52 (1:5 ethyl acetate:petroleum ether); 54% yield. The enantiomeric excess (*ee*) was determined by HPLC on a Chiralcel OD-H column (*n*-hexane/isopropanol = 95/5, flow rate 1 mL/min, λ = 254 nm), t_R = 6.73 min (major), t_R = 7.82 min (minor), 90% *ee*; ^{1}H-NMR (400 MHz, $CDCl_3$) δ 7.20–7.17 (m, 2H[2H′]), 6.59–6.54 (m, 2H[2H′]), 5.40 (s, 1H) and [5.43, (s, 1H′)], 4.86–4.82 (m, 1H[1H′]), 4.22–4.15 (m, 2H[2H′]), 3.75–3.64 (m, 2H[2H′]), 3.63–3.57 (m, 2H[2H′]), 3.31–3.27 (m, 1H[1H′]), 1.31–1.24 (m, 3H[3H′]), 1.22–1.16 (m, 6H[6H′]), ^{13}C-NMR (100 MHz, $CDCl_3$) δ 169.8, 143.4, 129.2, 123.5, 114.1 and [114.7, (1C′)], 87.9 and [88.0, (1C′)], 78.0, 72.6, 68.7 and [69.6, (1C′)], 61.6, 49.0 and [49.7, (1C′)], 22.1, 14.2. HRMS (ESI$^+$) exact mass calculated for $[M+Na]^+$ requires *m*/*z* 350.1130, found *m*/*z* 350.1131.

Ethyl 3-(4-methoxyphenyl)-5-phenyloxazolidine-2-carboxylate (**3j**) (diastereoisomers):

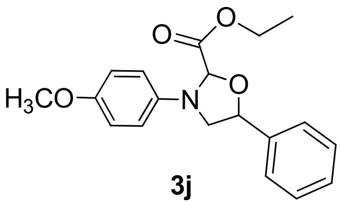

3j

Light yellow solid; M.P: 53–56 °C; R_f = 0.50 (1:5 ethyl acetate:petroleum ether); 42% yield. The enantiomeric excess (*ee*) was determined by HPLC on a Chiralcel OD-H column (*n*-hexane/isopropanol = 99/1, flow rate 0.30 mL/min, λ = 254 nm), t_R = 28.61 min (major), t_R = 30.667 min (minor), 84.6% *ee*; ^{1}H-NMR (400 MHz, $CDCl_3$) δ 7.50–7.48 (d, 2H), 7.33–7.32 (m, 3H), 6.79–6.73 (m, 2H), 6.71–6.66 (m, 2H), 5.50 (s, 1H) 4.36–4.27 (m, 2H), 4.24–4.16 (m, 2H), 4.05–4.02 (m, 1H), 3.72 (s, 3H) 1.40–1.37 (m, 3H), ^{13}C-NMR (100 MHz, $CDCl_3$) δ 170.70, 153.06,139.84, 139.19, 128.91, 127.83, 126.52, 114.88, 114.65, 90.85, 75.37, 63.75, 61.53, 55.59, 14.21. HRMS (EI$^+$) exact mass calculated for $C_{19}H_{21}NO_4$ $[M]^+$ requires *m*/*z* 327.1471, found *m*/*z* 327.1471.

NOESY experiment and HMBC experiment also identified the structure of products, see the spectra in Supporting Information.

4. ^{1}H-NMR and ^{13}C-NMR Spectra and HPLC of Products

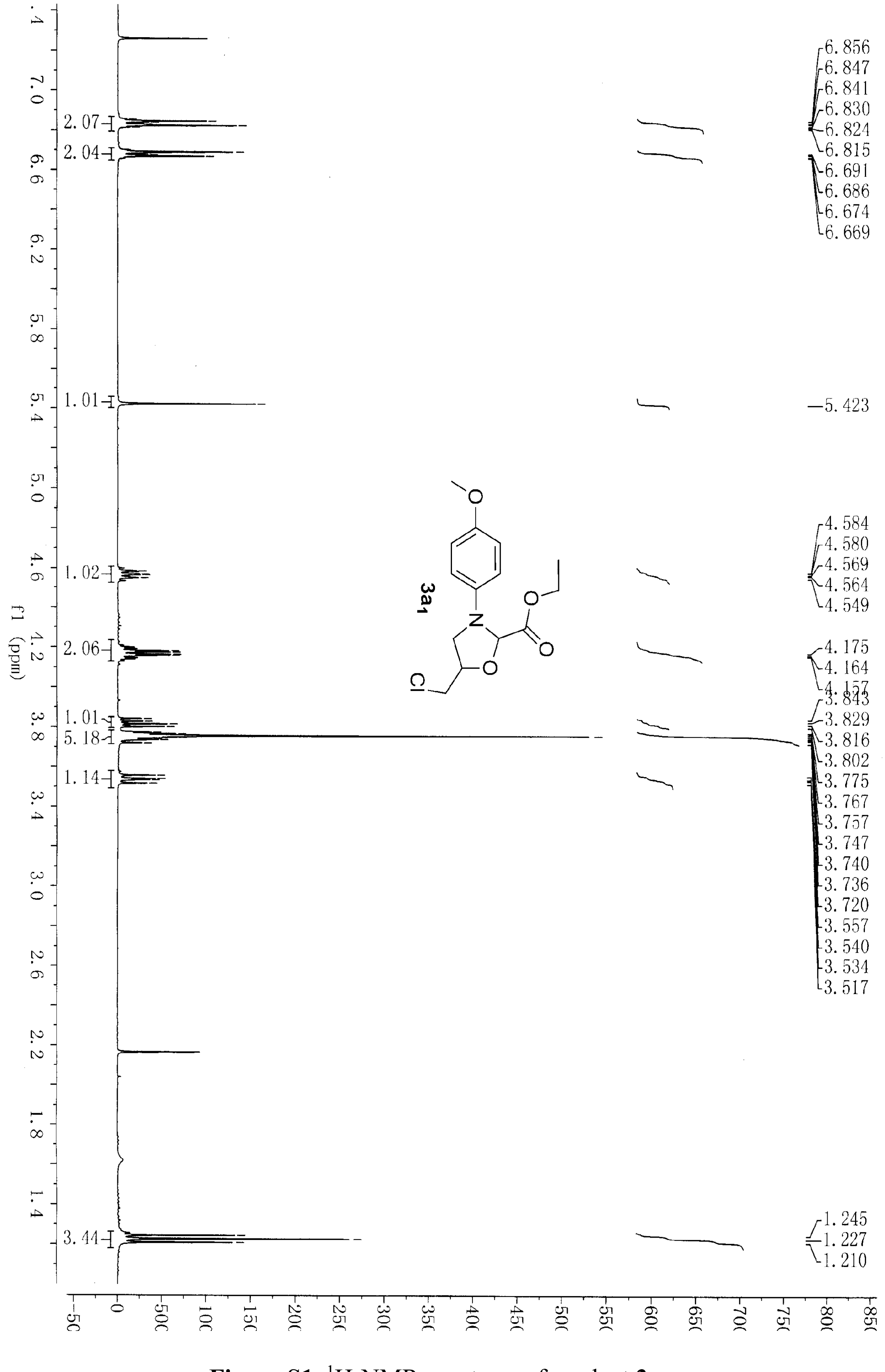

Figure S1. ^{1}H-NMR spectrum of product **3a$_1$**.

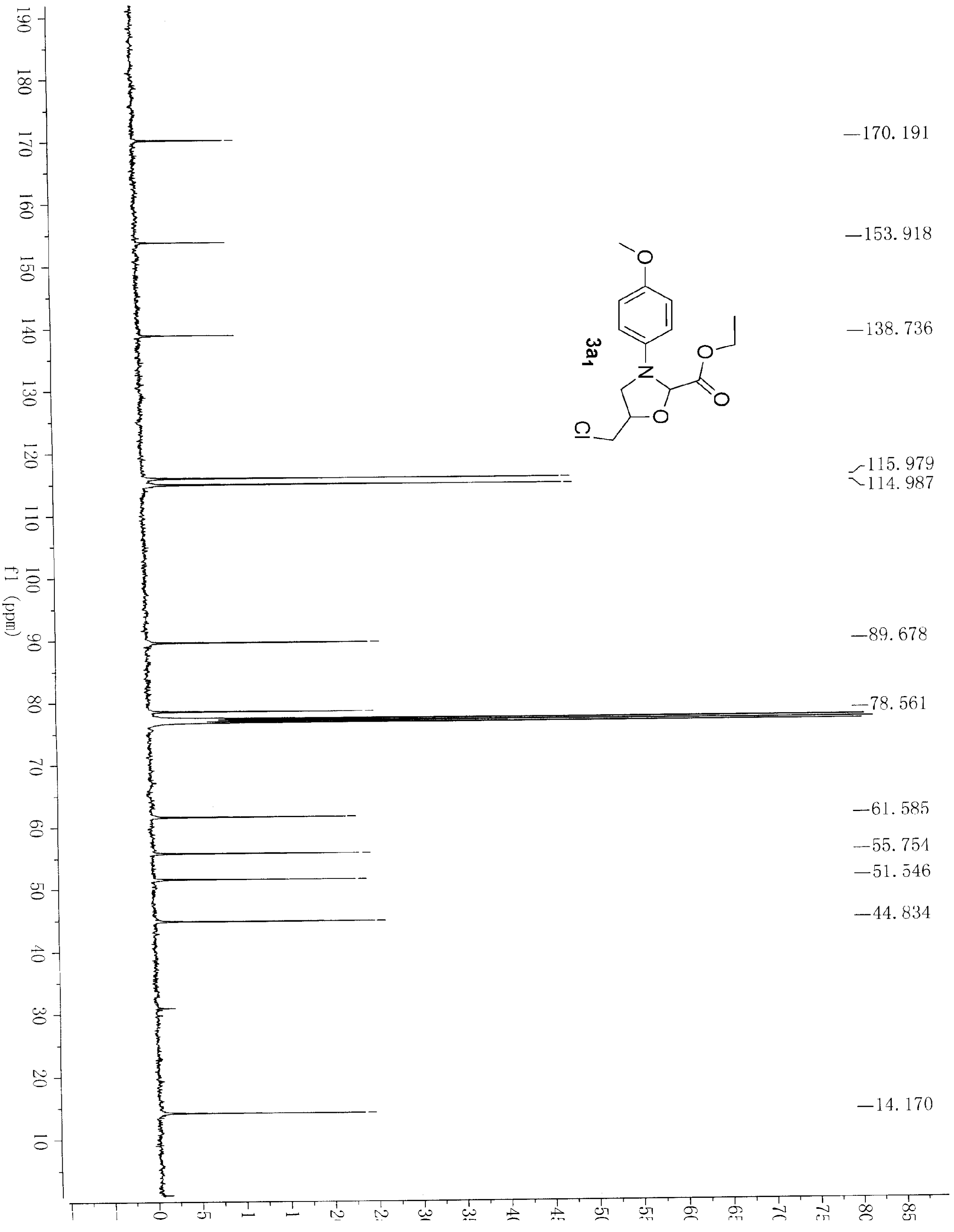

Figure S2. ^{13}C-NMR spectrum of product **3a$_1$**.

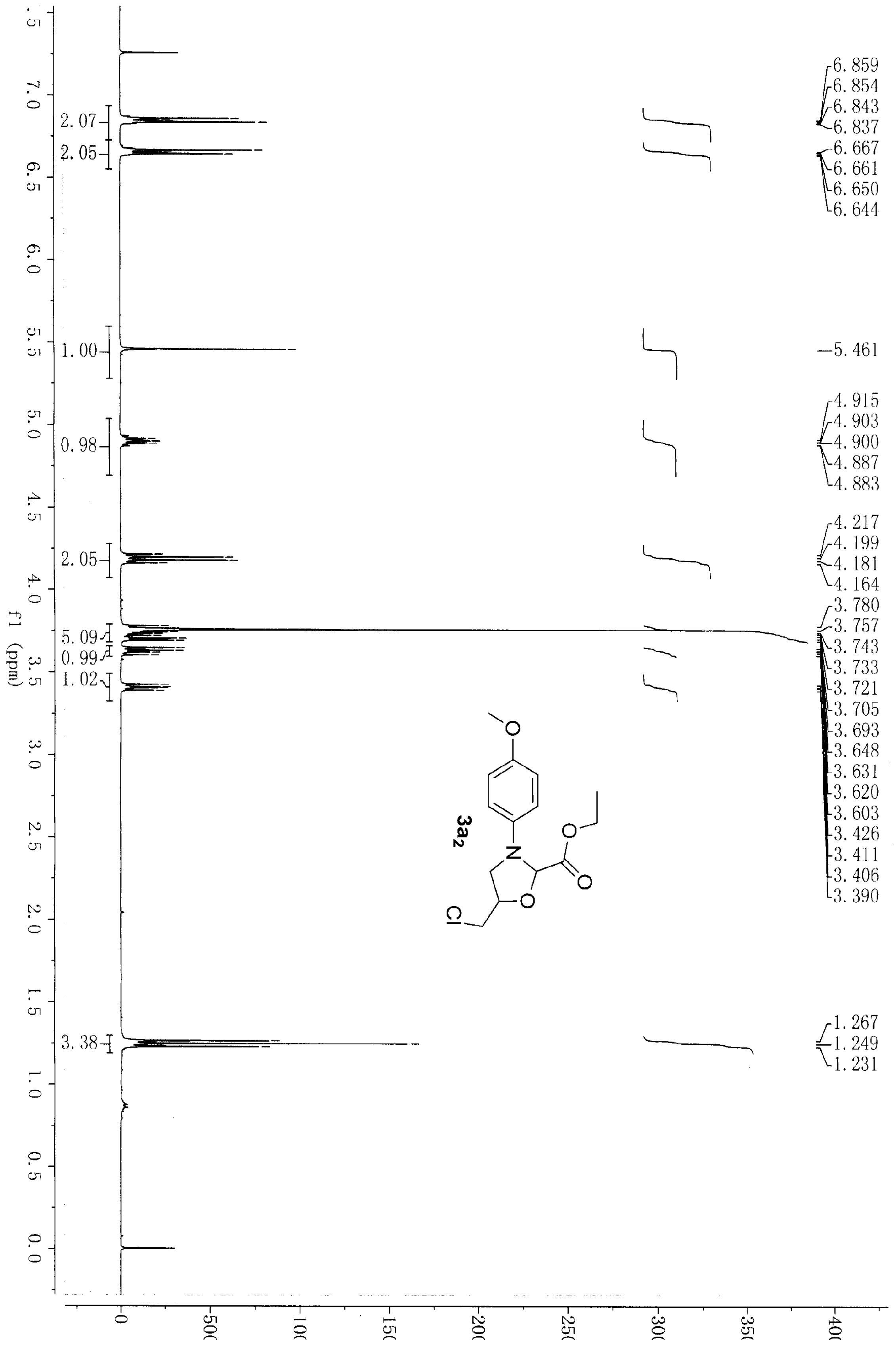

Figure S3. ^{1}H-NMR spectrum of product **3a$_2$**.

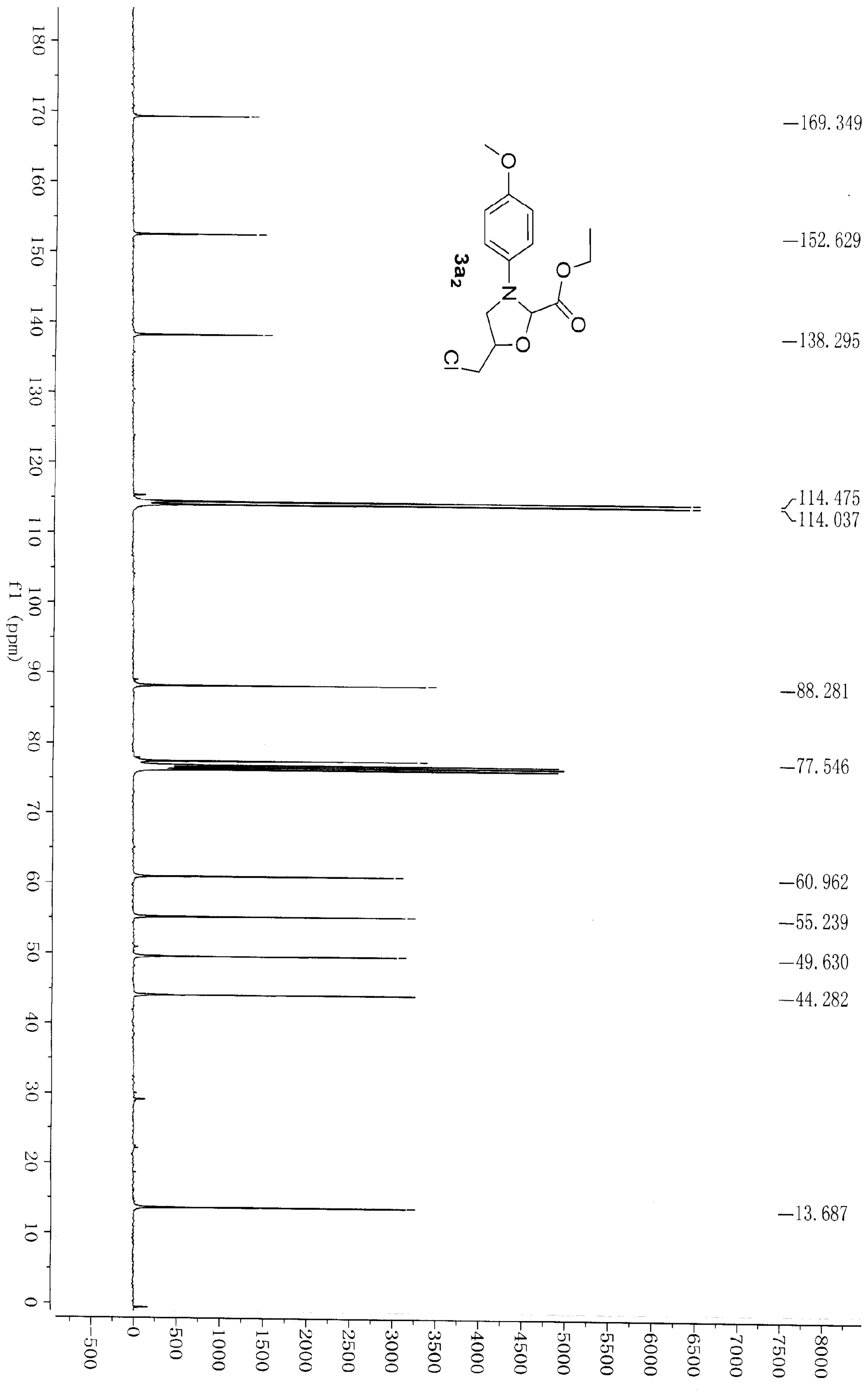

Figure S4. ^{13}**C-NMR** spectrum of product **3a**$_2$.

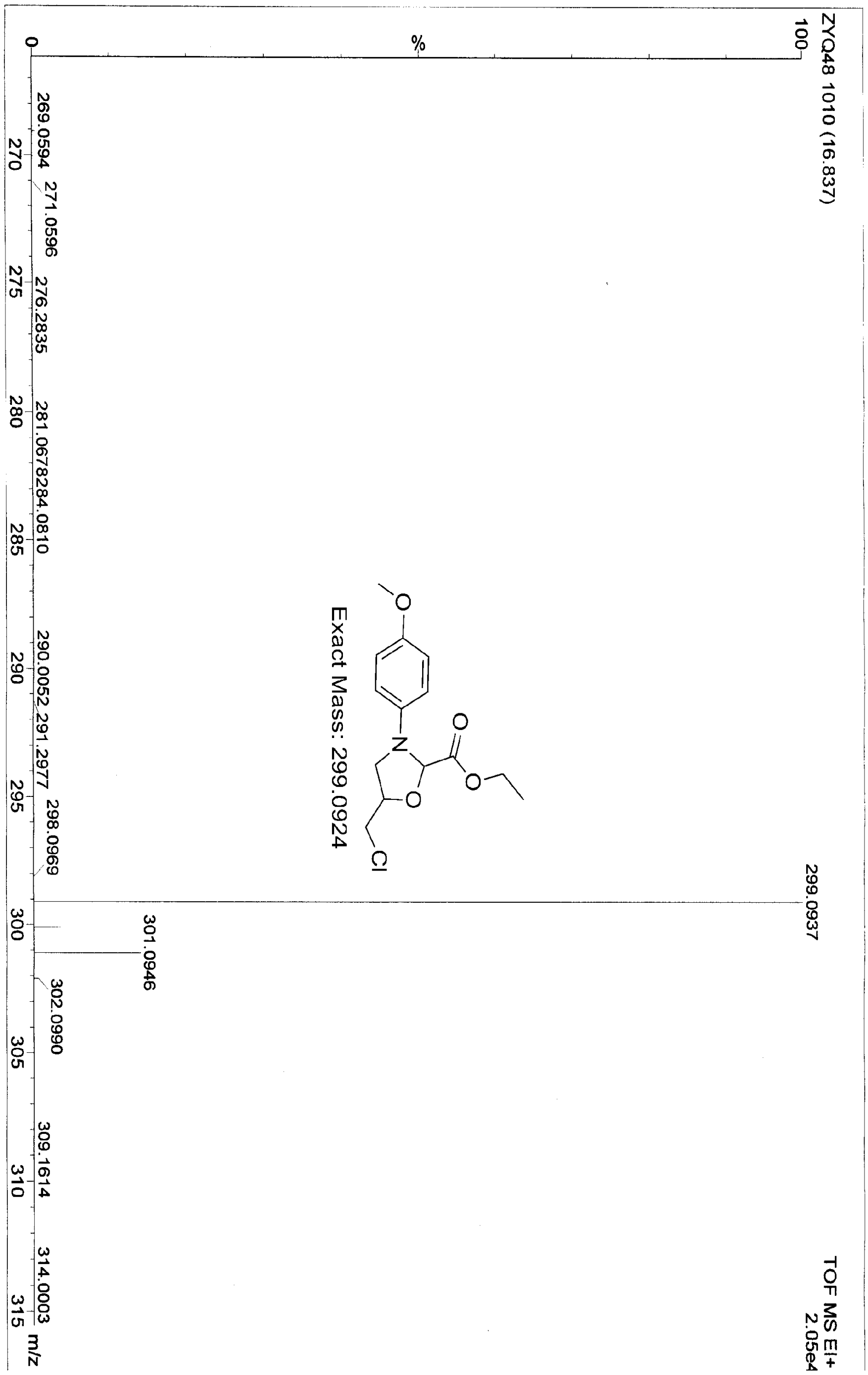

Figure S5. HRMS spectrum of product **3a**.

Table S1. HPLC analysis of product **3a**.

Column	CHIRALCEL OD-H(ODH0CE-LA084)
Column size	0.46 cm I.D.*25 cm L
Injection	20 μL
Mobile phase	*n*-Hexane/2-propanol = 95/5 (v/v)
Flow rate	0.5 mL/min
Wave length	UV 254 nm
Temperature	25 °C
Solvents	Hexane, 2-propanol: HPLC grade

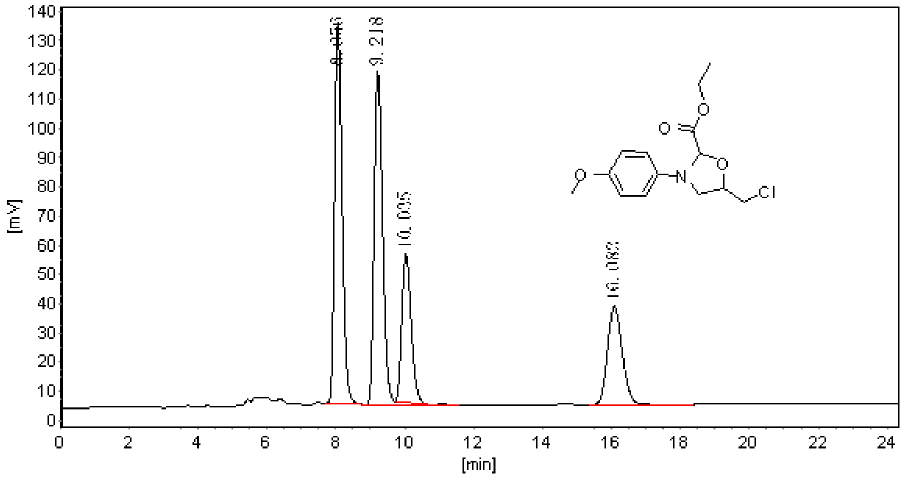

Peak NO.	Time	Height	Area	Area %
1	8.056	129400	1961968	33.18
2	9.218	113407	1976474	33.42
3	10.035	50131	956629	16.17
4	16.082	33578	1017946	17.23

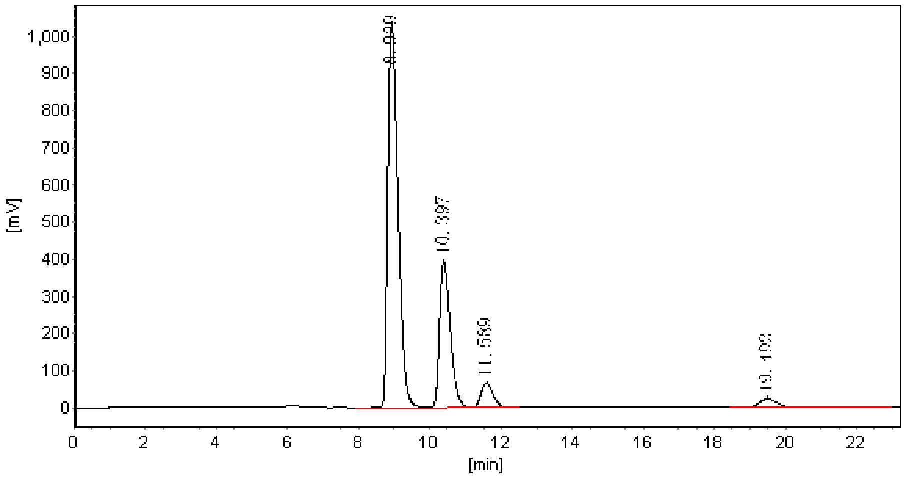

Peak NO.	Time	Height	Area	Area %
1	8.929	1033708	20719776	66.03
2	10.397	394183	8337735	26.57
3	11.589	64884	1516792	4.85
4	19.492	22418	802364	2.55

Figure S6. *Cont.*

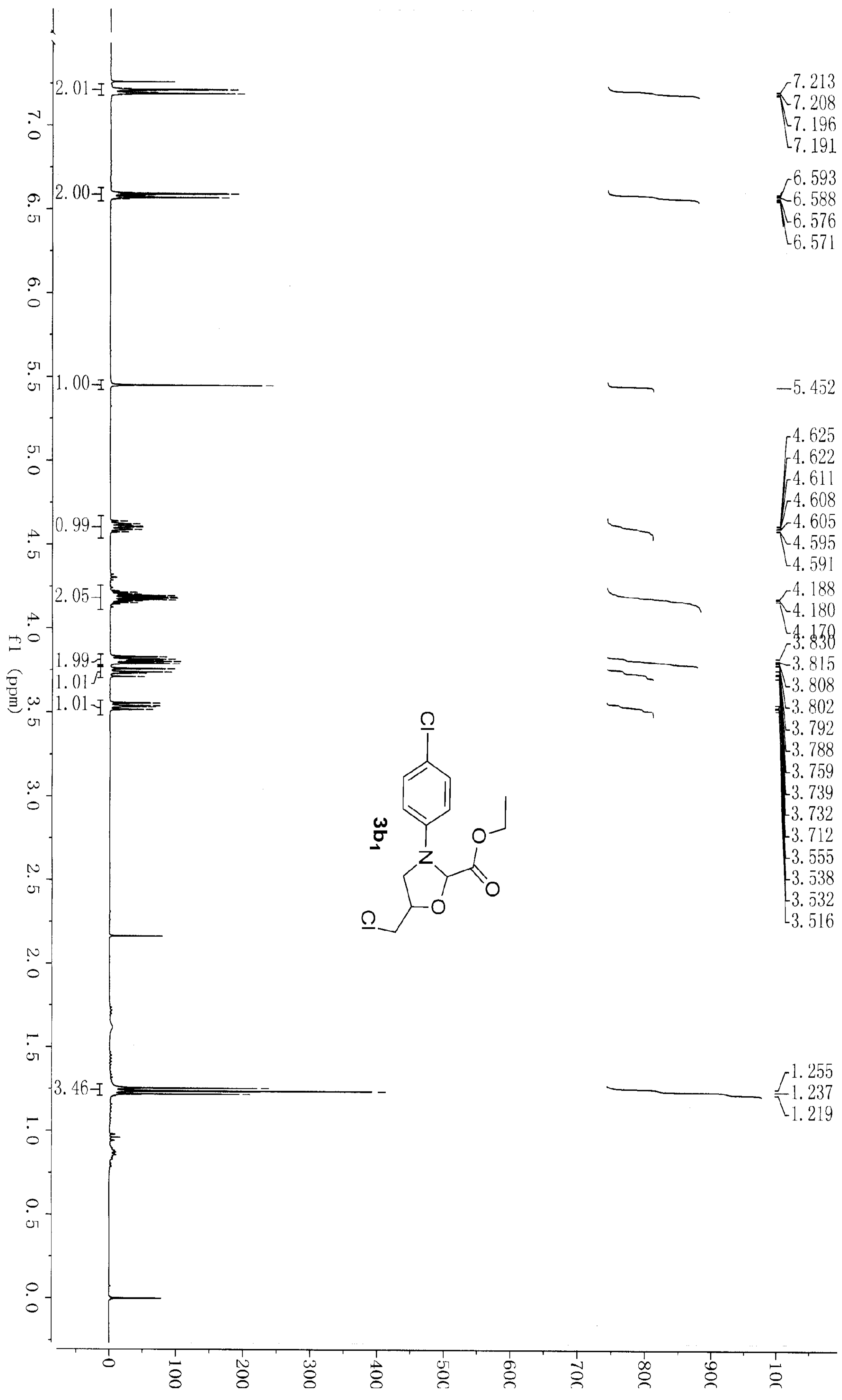

Figure S6. ^{1}H-NMR spectrum of product **3b$_1$**.

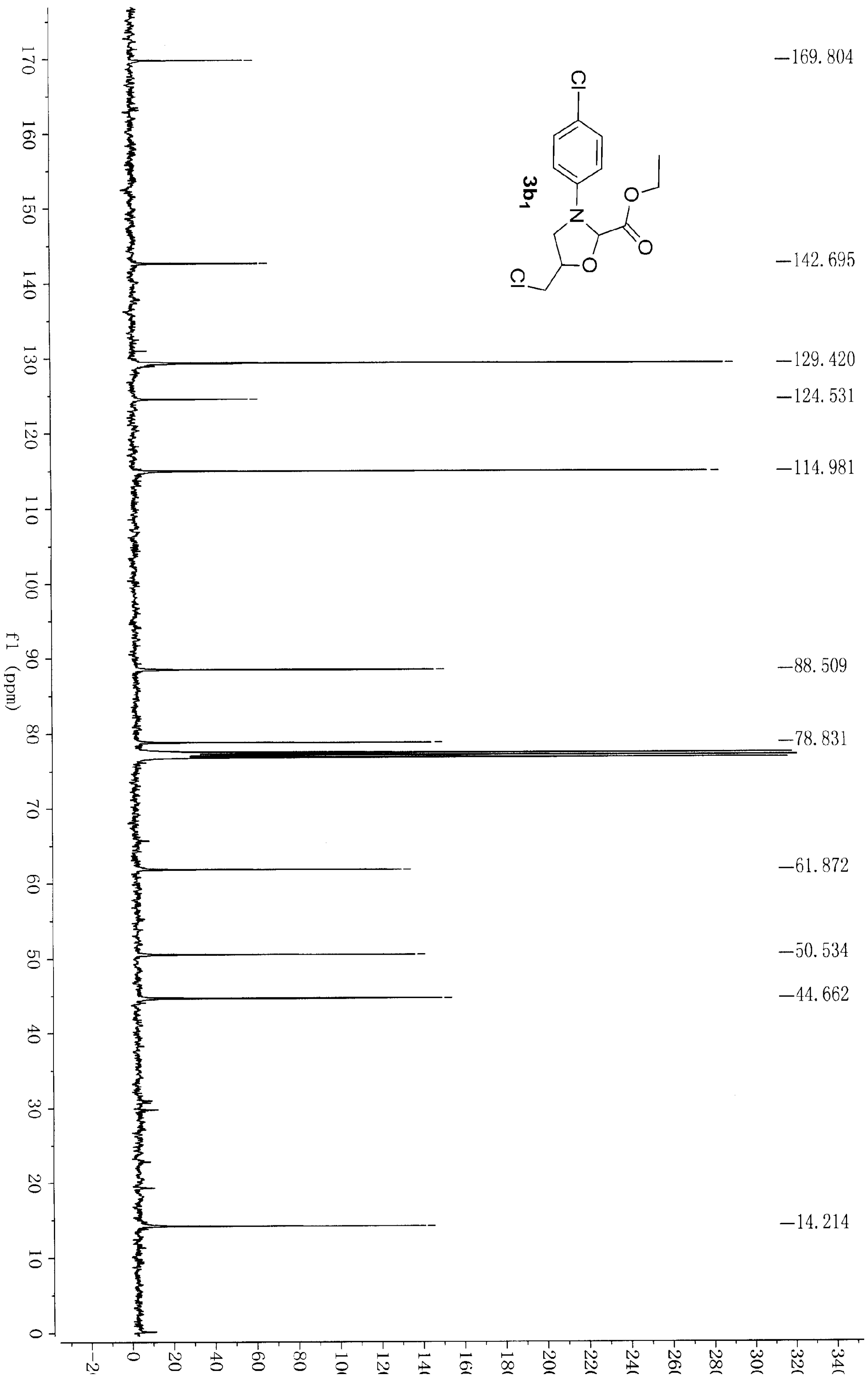

Figure S7. ^{13}C-NMR spectrum of product **3b$_1$**.

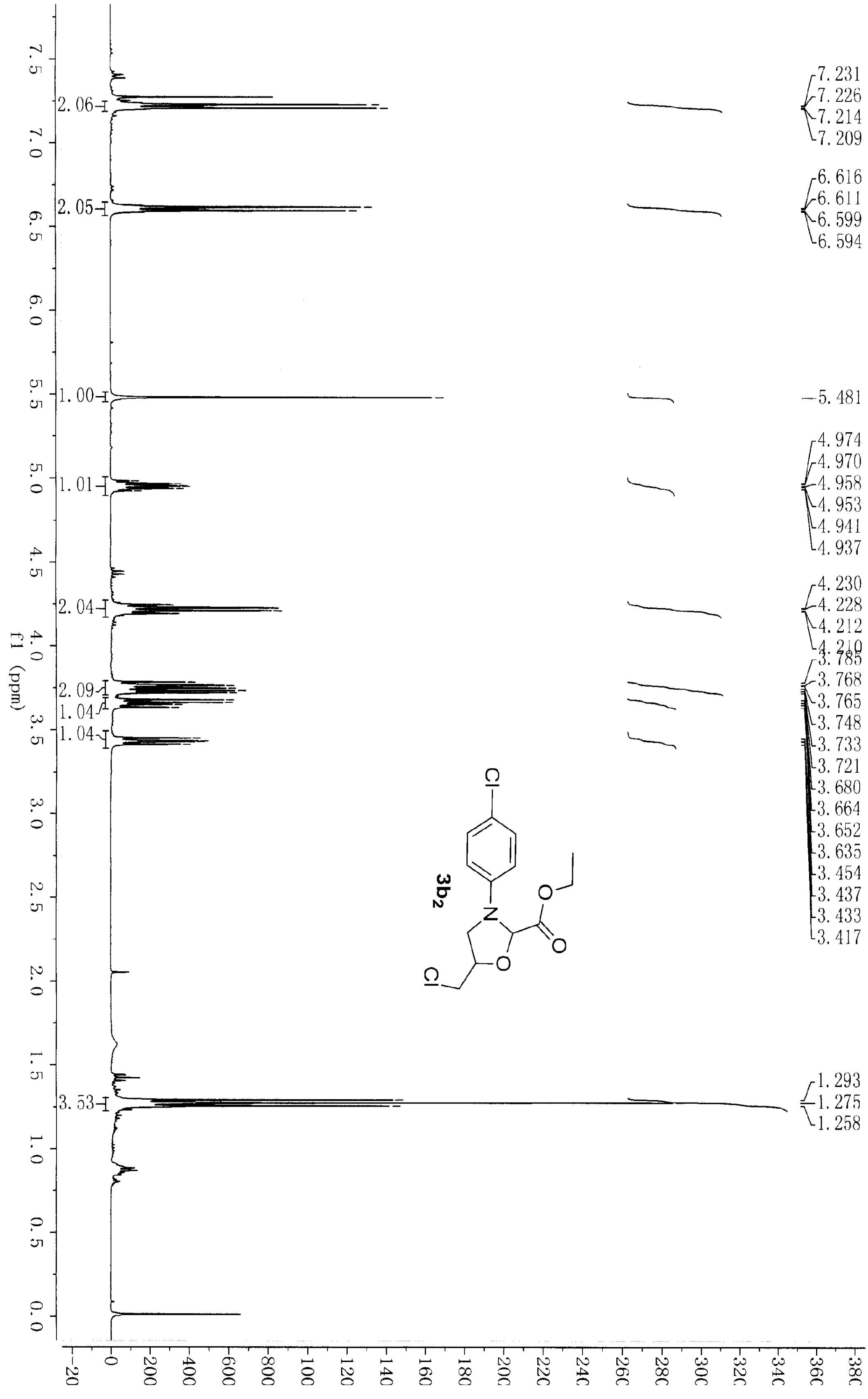

Figure S8. ^{1}H-NMR spectrum of product **3b$_2$**.

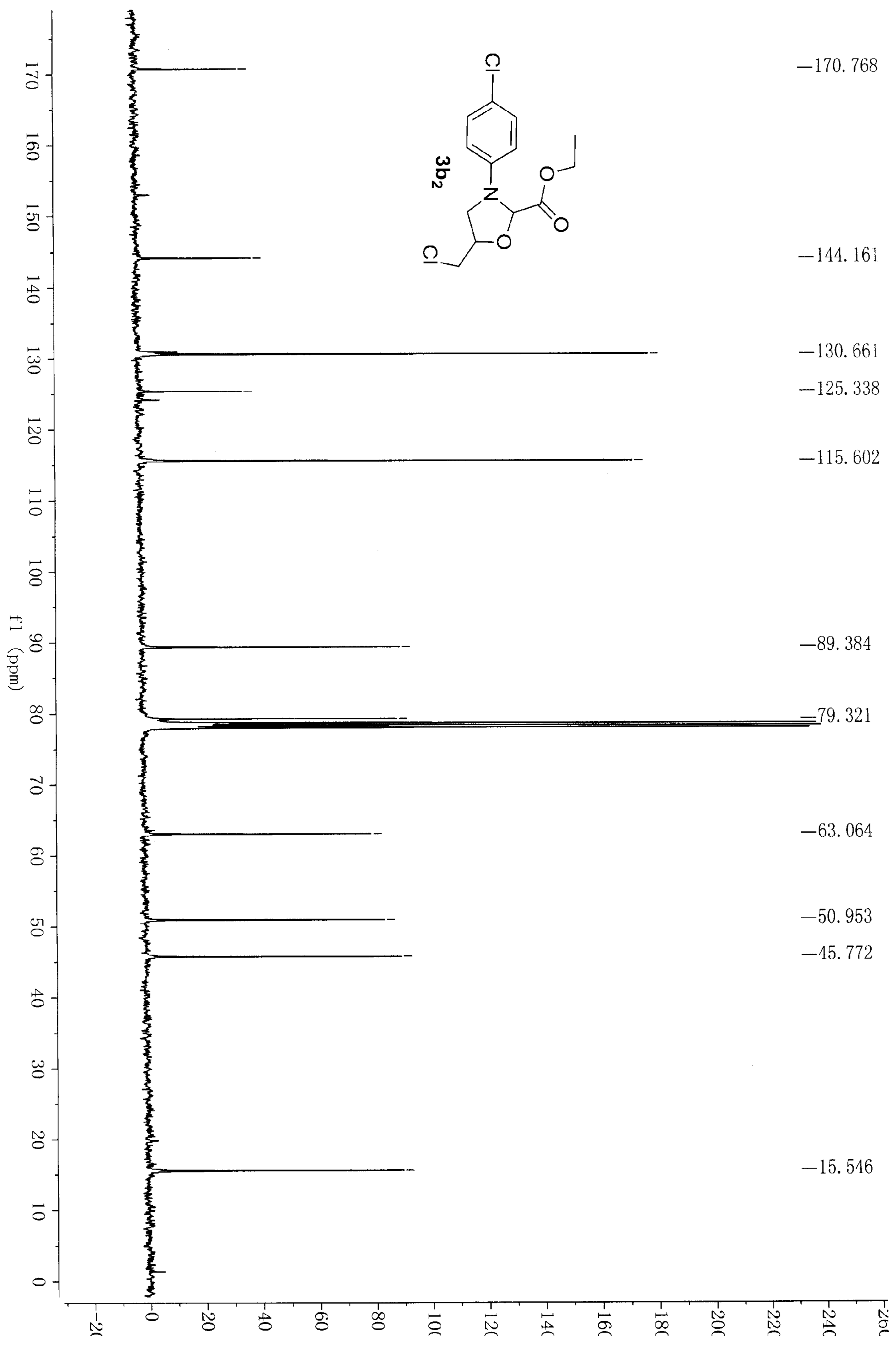

Figure S9. ^{13}C-NMR spectrum of product **3b$_2$**.

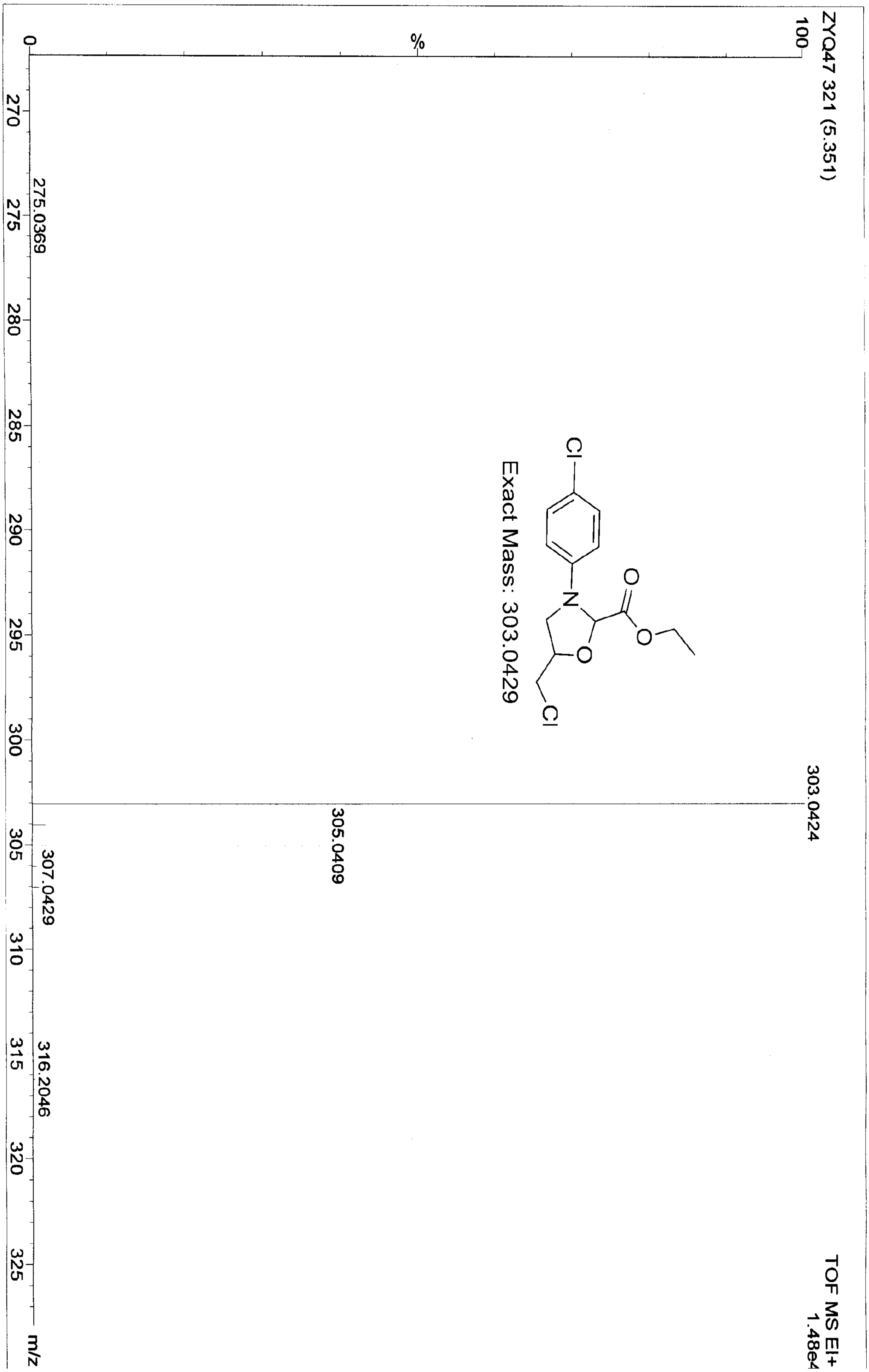

Figure S10. HRMS spectrum of product **3b**.

Table S2.HPLC analysis of product **3b**.

Column	CHIRALCEL OD-H(ODH0CE-LA084)
Column size	0.46 cm I.D.*25 cm L
Injection	20 μL
Mobile phase	*n*-Hexane/2-propanol = 95/5 (v/v)
Flow rate	1.0 mL/min
Wave length	UV 254 nm
Temperature	25 °C
Solvents	Hexane, 2-propanol: HPLC grade

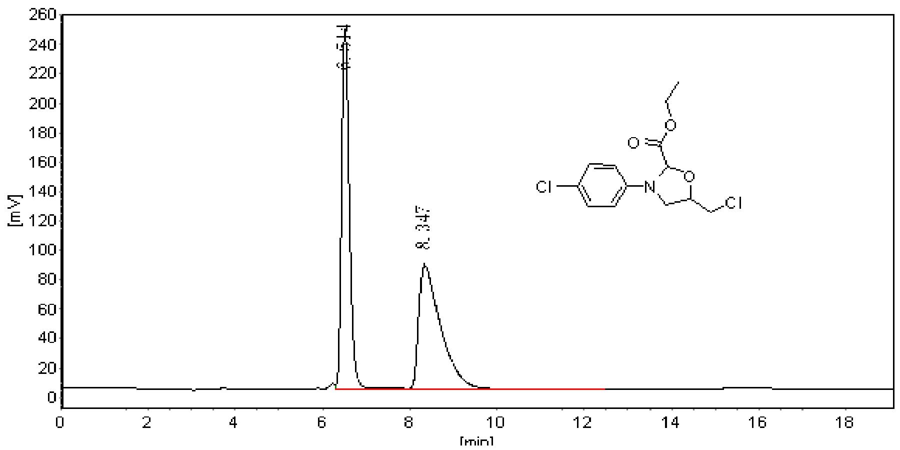

Peak NO.	Time	Height	Area	Area %
1	6.514	242473	3021599	51.06722
2	8.347	83604	2895306	48.93278

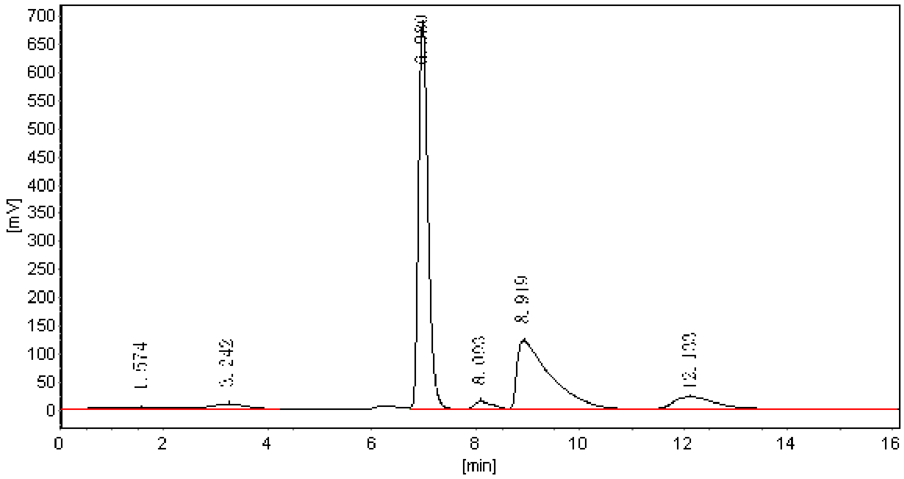

Peak NO.	Time	Height	Area	Area %
1	6.980	684506	8797170	53.75
2	8.093	13468	301372	1.84
3	8.919	121180	5434782	33.20
4	12.133	21182	1245096	7.61

Figure S11. *Cont.*

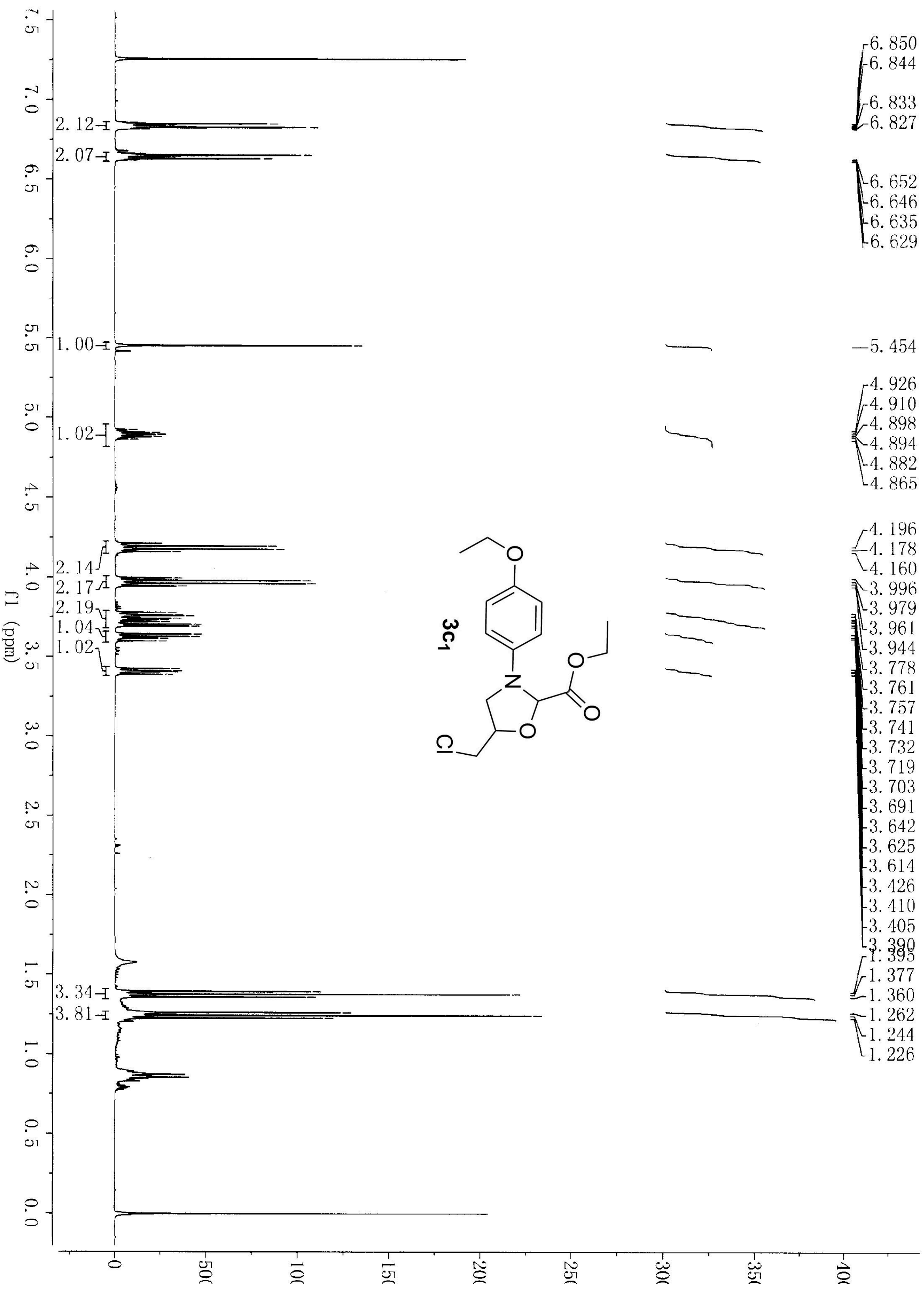

Figure S11. ^{1}H-NMR spectrum of product **3c$_1$**.

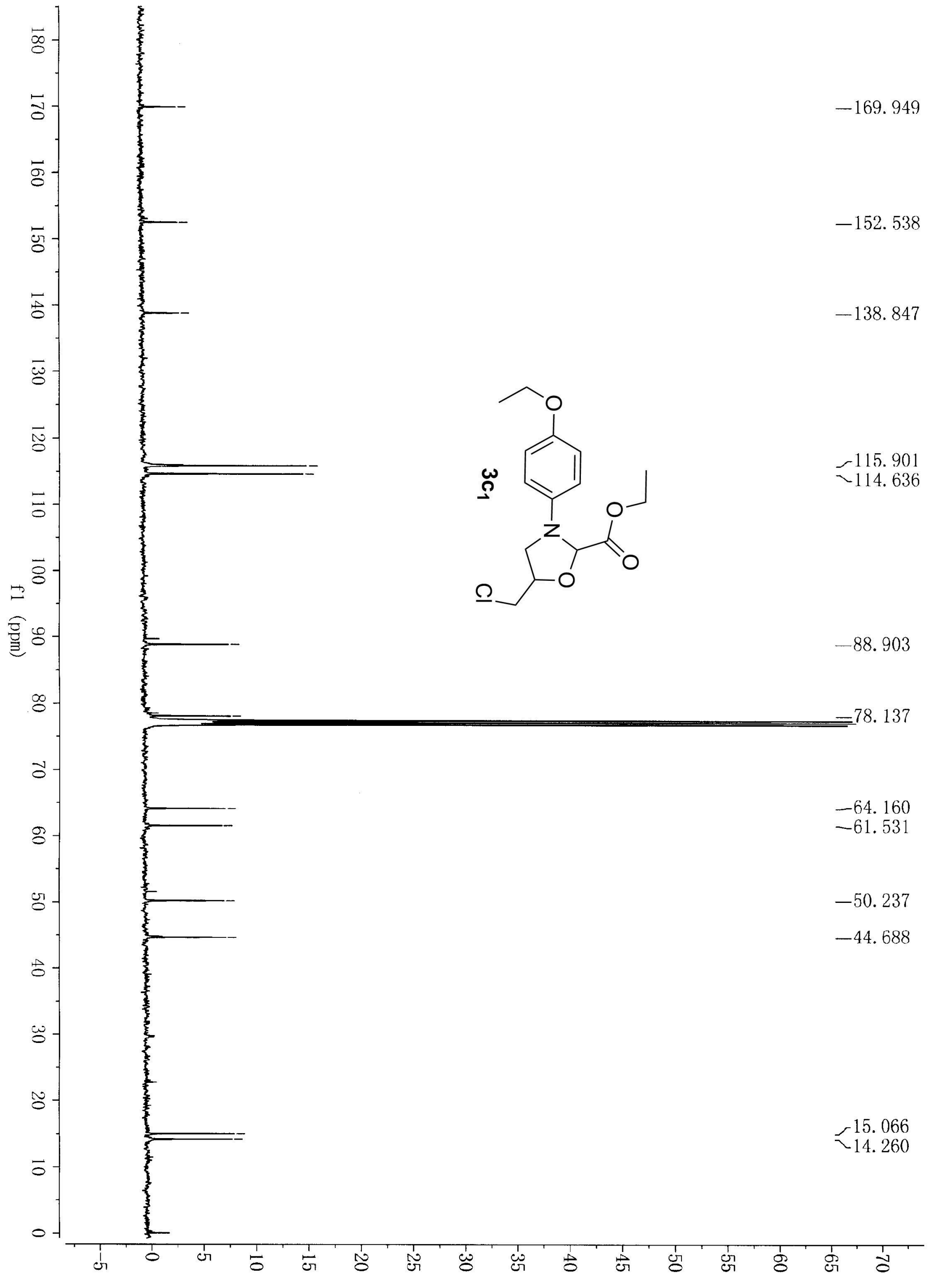

Figure S12. ^{13}C-NMR spectrum of product **3c$_1$**.

$C_{15}H_{20}ClNO_4$

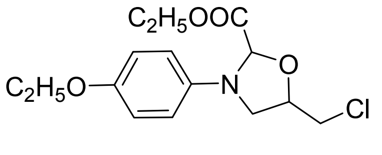

3c

3c. $C_{15}H_{20}ClNO_4$ **HRMS** (ESI$^+$) exact mass calculated for $[M + H]^+$ requires *m*/*z* 314.1154, found *m*/*z* 314.1155.

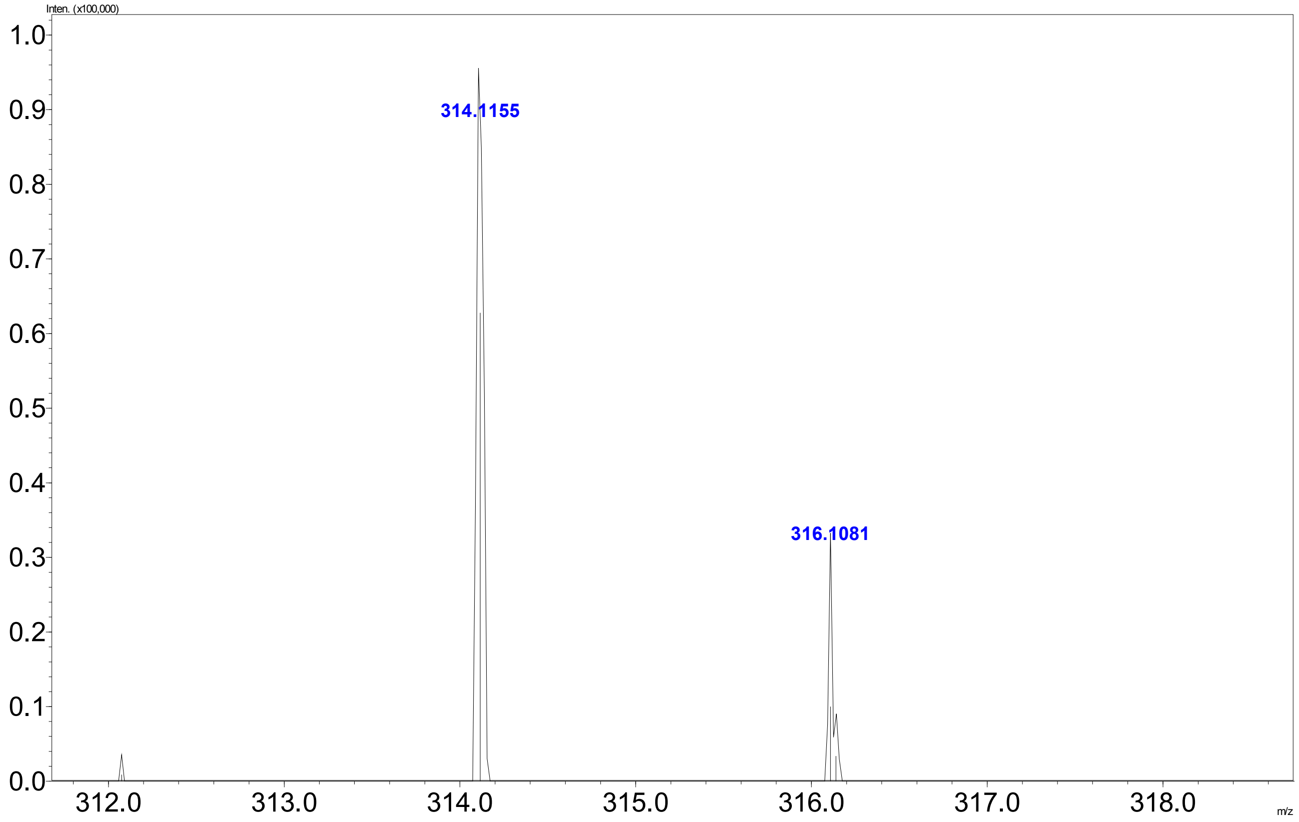

Figure S13. HRMS spectrum of product **3c.**

Table S3. HPLC analysis of product **3c**.

**Column**	**CHIRALCEL OD-H(ODH0CE-LA084)**
Column size	0.46 cm I.D.*25 cm L
Injection	20 μL
Mobile phase	*n*-Hexane/2-propanol = 95/5 (v/v)
Flow rate	0.5 mL/min
Wave length	UV 254 nm
Temperature	25 °C
Solvents	Hexane, 2-propanol: HPLC grade

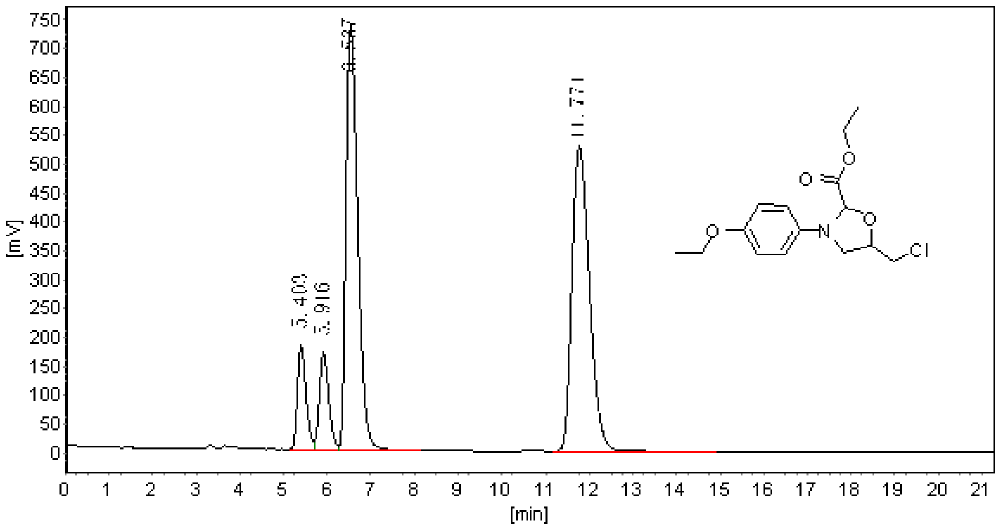

Peak NO.	Time	Height	Area	Area %
1	5.403	178396	2475583	7.38999
2	5.916	166075	2499357	7.46096
3	6.537	731553	13785348	40.95784
4	11.771	525642	14738841	43.79078

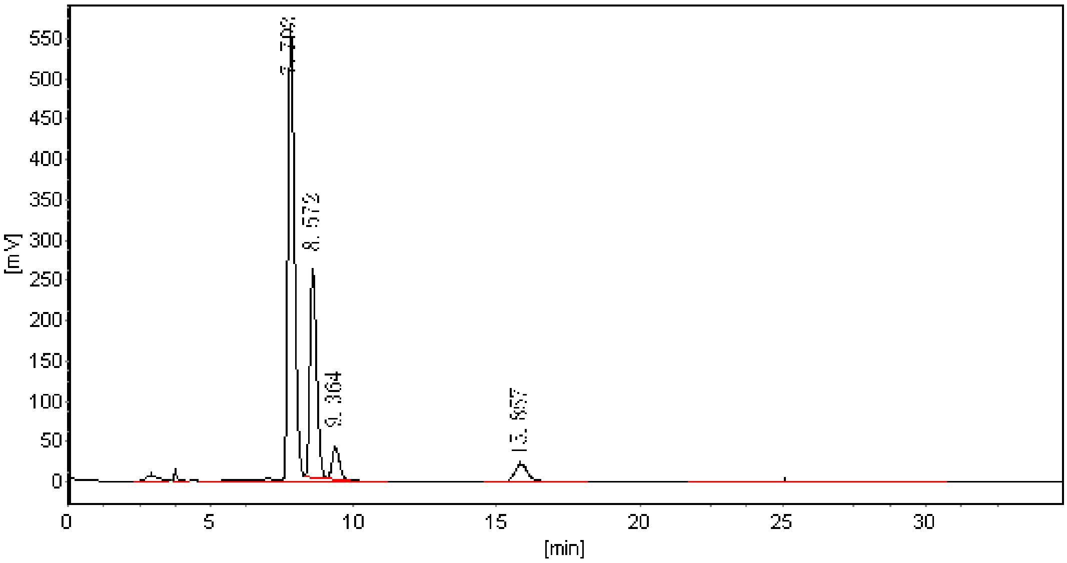

Peak NO.	Time	Height	Area	Area %
1	7.792	563129	9516917	62.92
2	8.572	256372	4221726	27.91
3	9.364	38902	711214	4.70
4	15.857	21379	676902	4.47

Figure S14. *Cont.*

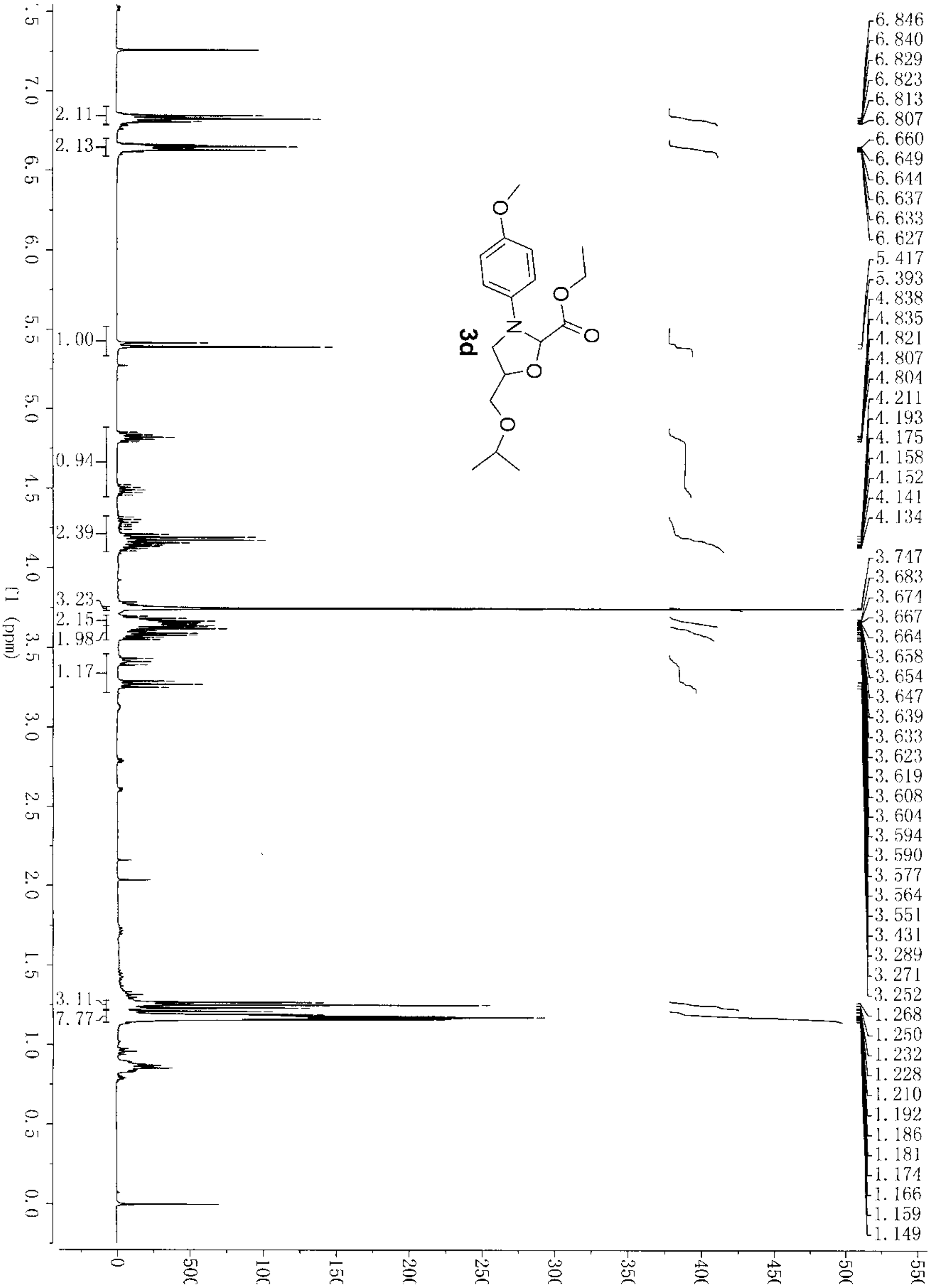

Figure S14. ^{1}H-NMR spectrum of product **3d**.

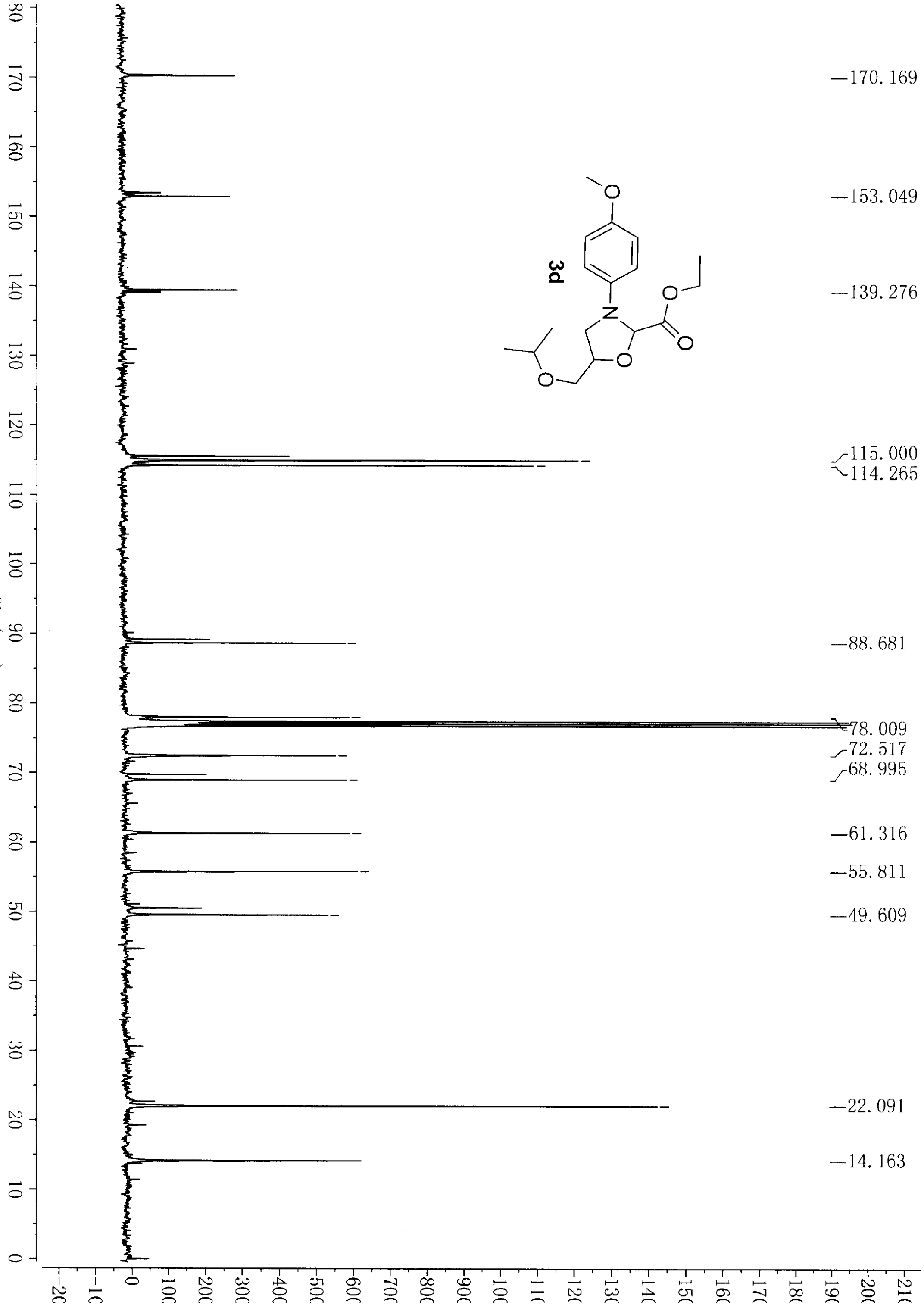

Figure S15. ^{13}C-NMR spectrum of product **3d**.

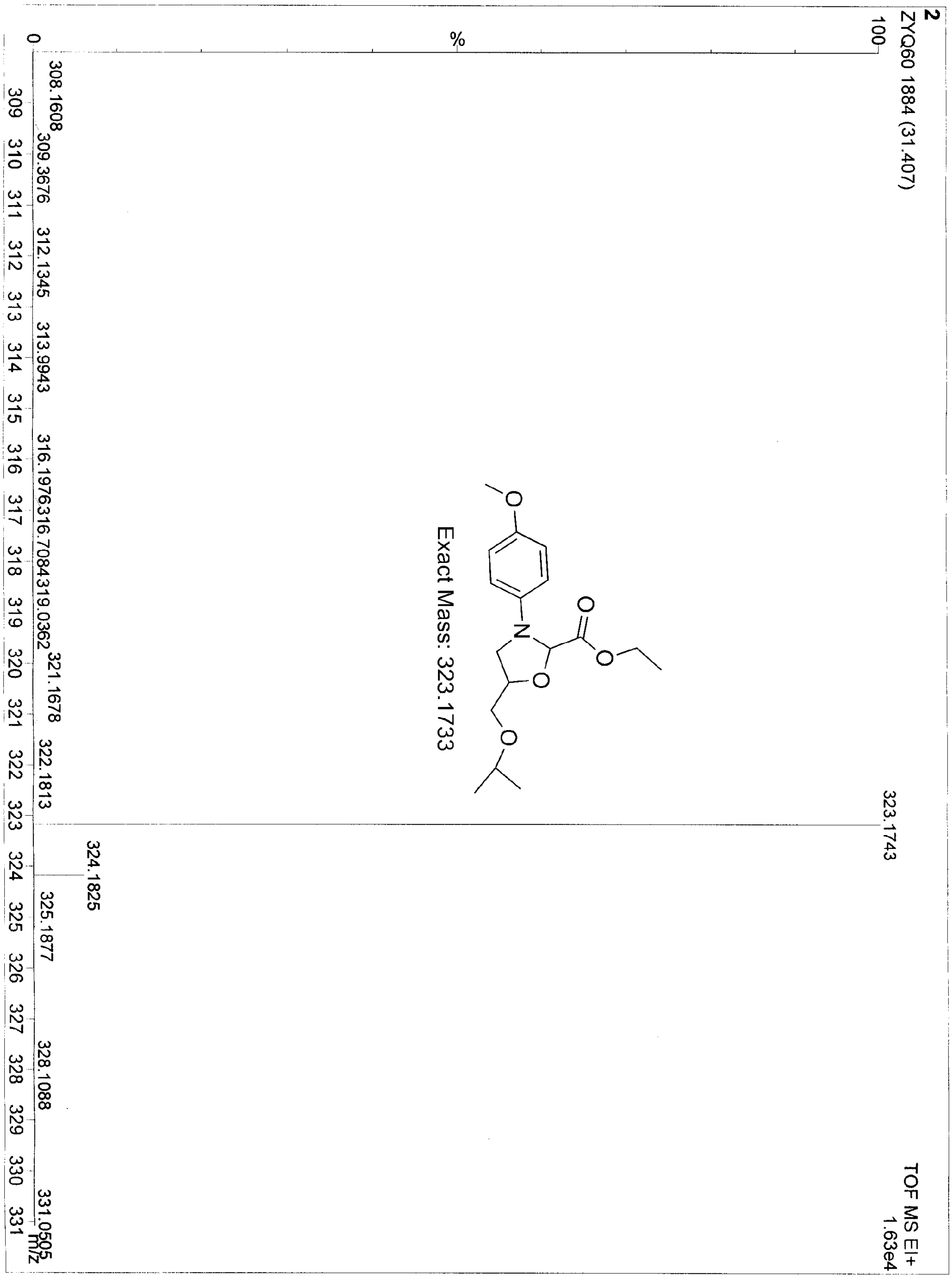

Figure S16. HRMS spectrum of product **3d**.

Table S4. HPLC analysis of product **3d**.

**Column**	**CHIRALCEL OD-H(ODH0CE-LA084)**
Column size	0.46 cm I.D.*25 cm L
Injection	20 μL
Mobile phase	*n*-Hexane/2-propanol = 95/5 (v/v)
Flow rate	0.5 mL/min
Wave length	UV 254 nm
Temperature	25 °C
Solvents	Hexane, 2-propanol: HPLC grade

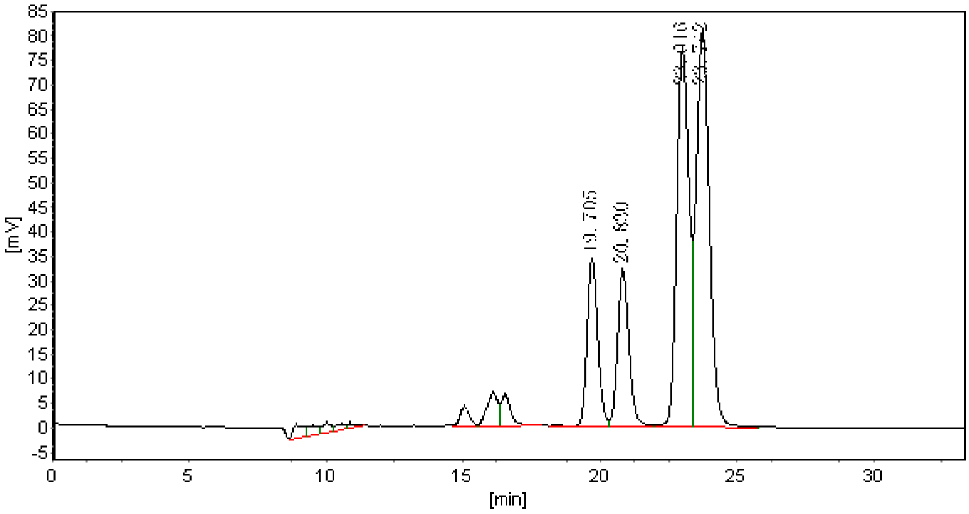

Peak NO.	Time	Height	Area	Area %
1	19.705	33897	913178	13.01
2	20.830	31735	927902	13.23
3	23.016	77134	2406588	34.31
4	23.742	80733	2766602	39.44

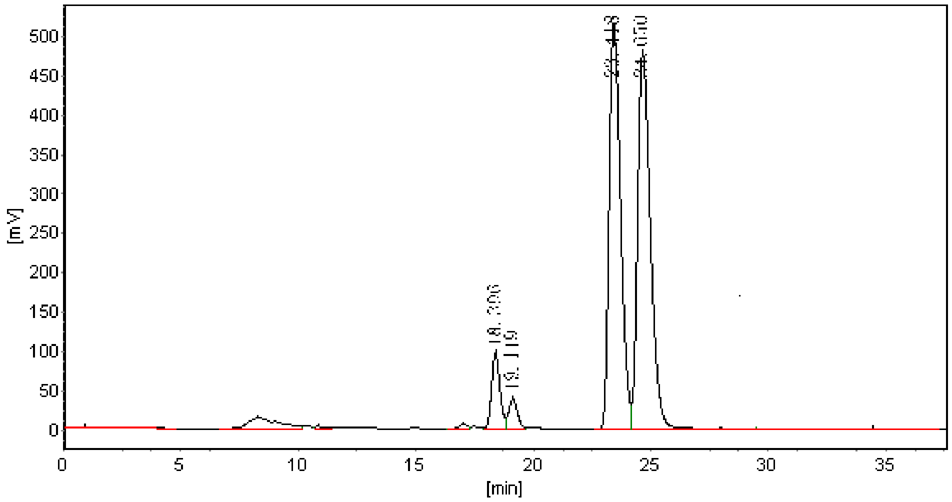

Peak NO.	Time	Height	Area	Area %
1	18.396	96389	2387657	6.10
2	19.119	38401	985455	2.52
3	23.418	513120	17460244	44.67
4	24.650	478399	18258818	46.71

Figure S17. *Cont.*

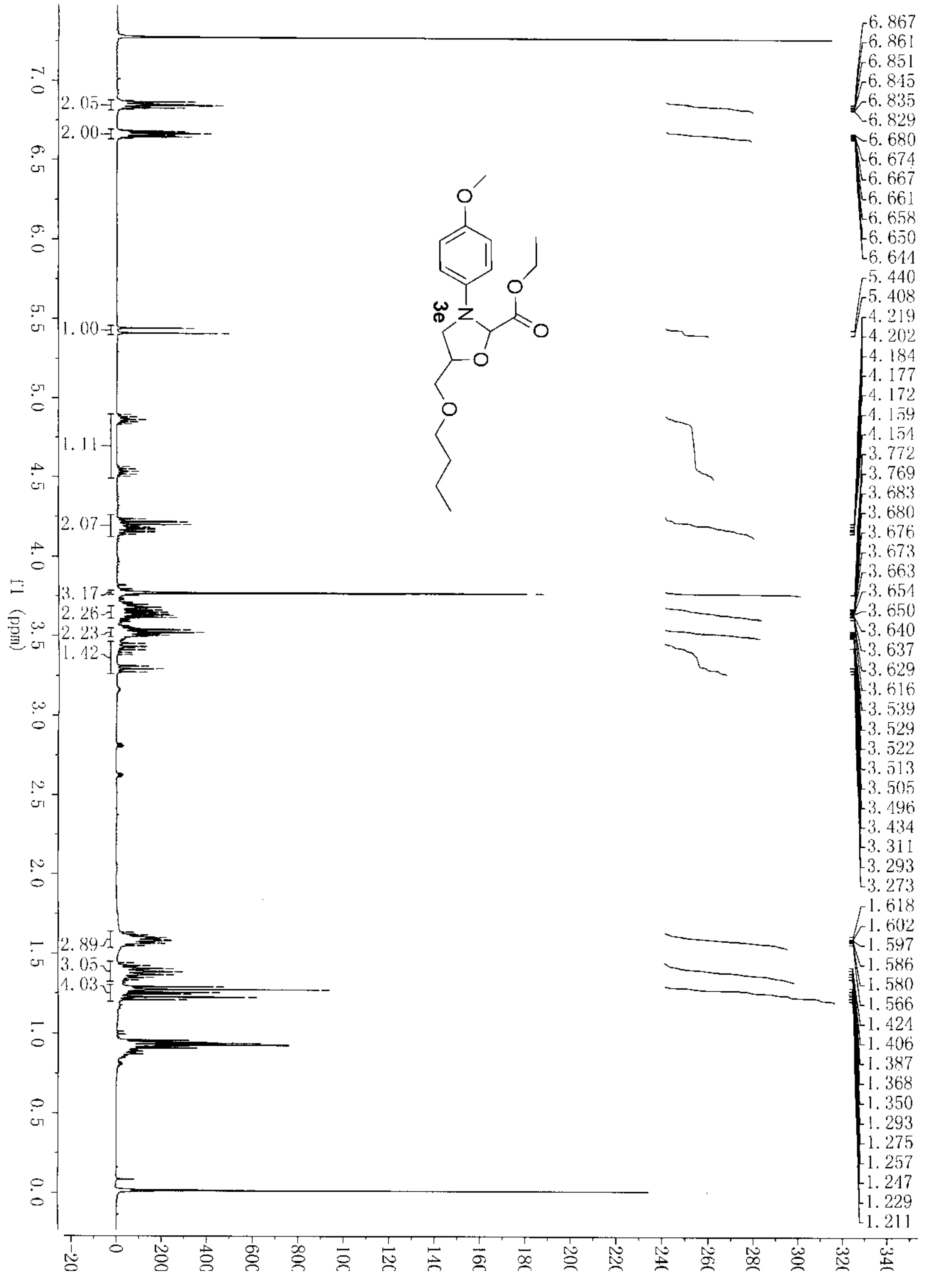

Figure S17. ^{1}H-NMR spectrum of product **3e**.

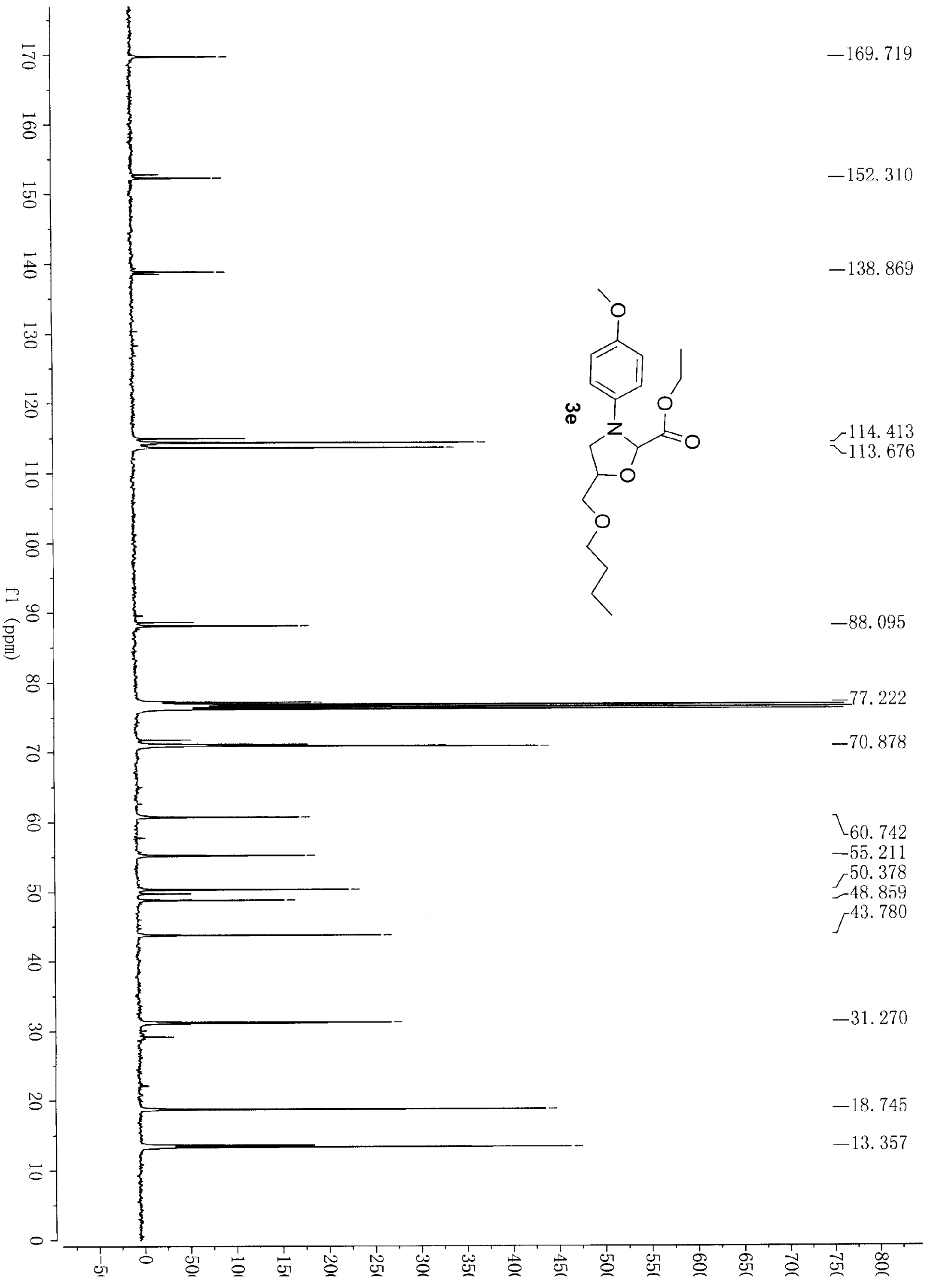

Figure S18. ^{13}C-NMR spectrum of product **3e**.

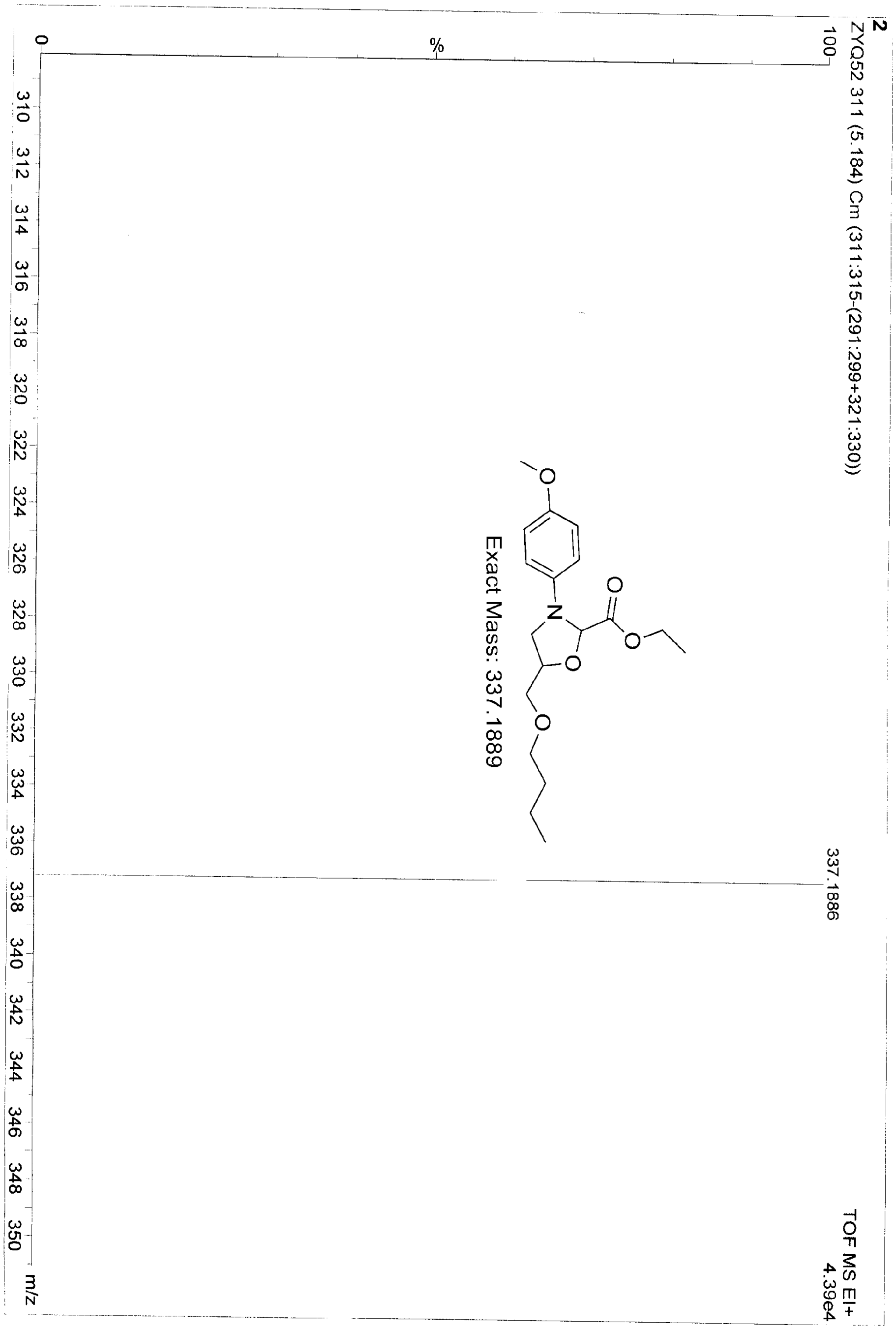

Figure S19. HRMS spectrum of product **3e**.

Table S5. HPLC analysis of product **3e**.

**Column**	**CHIRALCEL OD-H(ODH0CE-LA084)**
Column size	0.46 cm I.D.*25 cm L
Injection	20 μL
Mobile phase	*n*-Hexane/2-propanol = 95/5 (v/v)
Flow rate	0.5 mL/min
Wave length	UV 254 nm
Temperature	25 °C
Solvents	Hexane, 2-propanol: HPLC grade

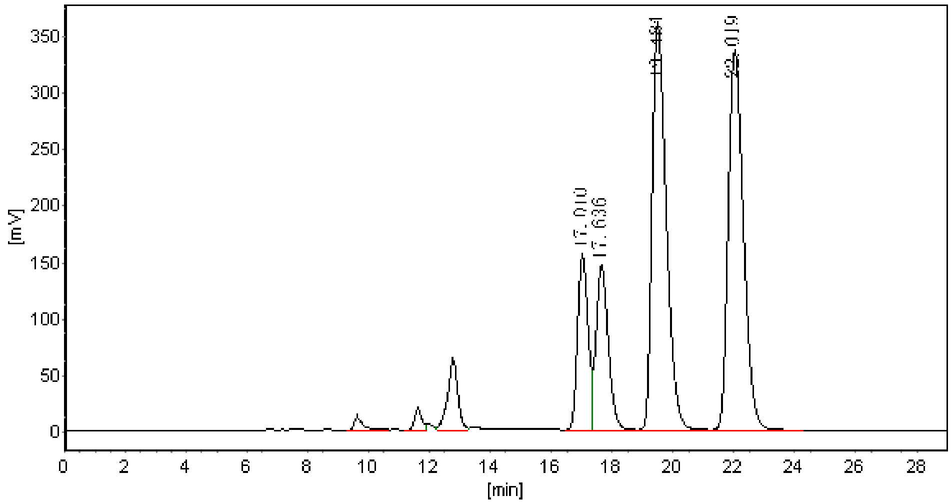

Peak NO.	Time	Height	Area	Area %
1	17.010	154262	3744466	12.00
2	17.636	144841	4065326	13.03
3	19.484	359199	11692550	37.48
4	22.019	335271	11693701	37.49

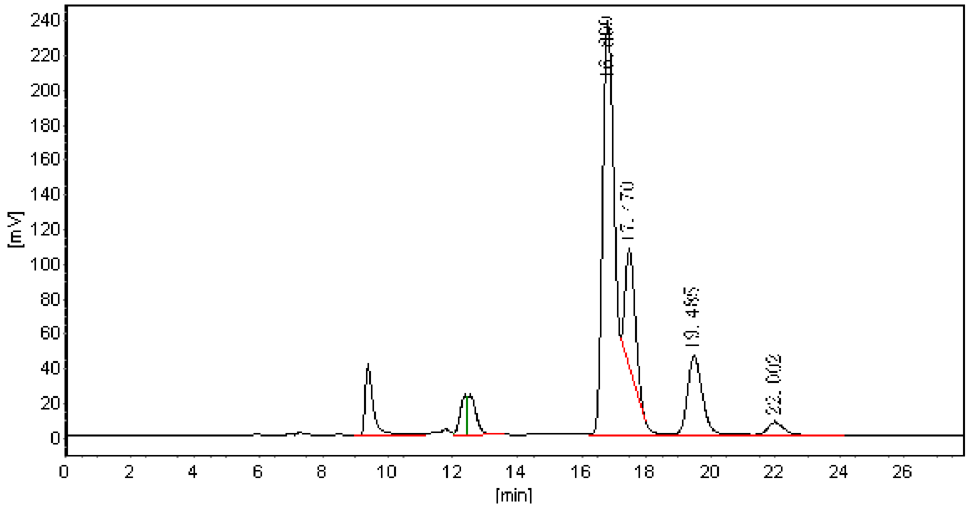

Peak NO.	Time	Height	Area	Area %
1	16.809	235497	7498214	70.79
2	17.470	66225	1421288	13.42
3	19.485	45011	1429110	13.49
4	22.002	6986	242711	2.30

Figure S20. *Cont.*

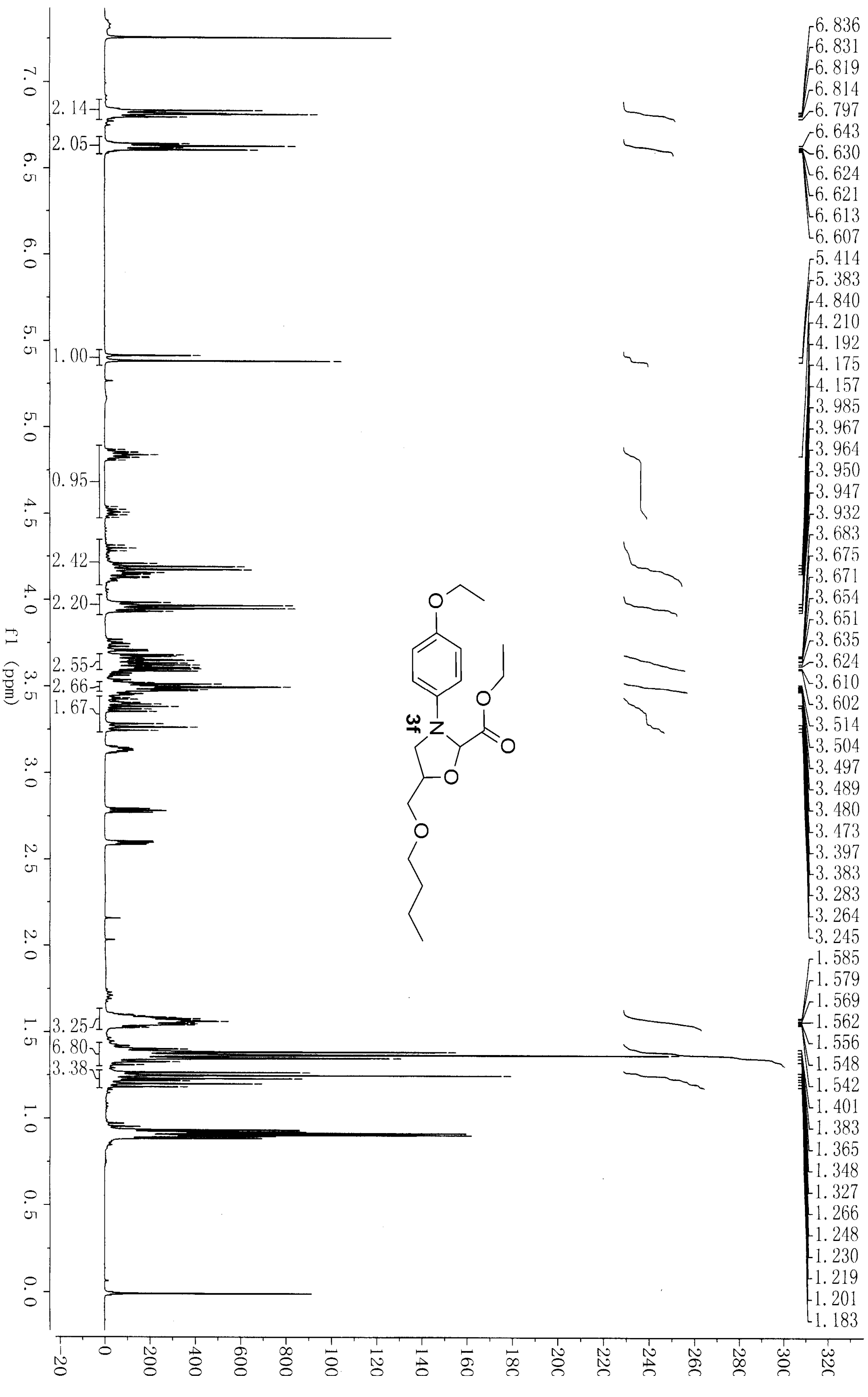

Figure S20. ^{1}H-NMR spectrum of product **3f**.

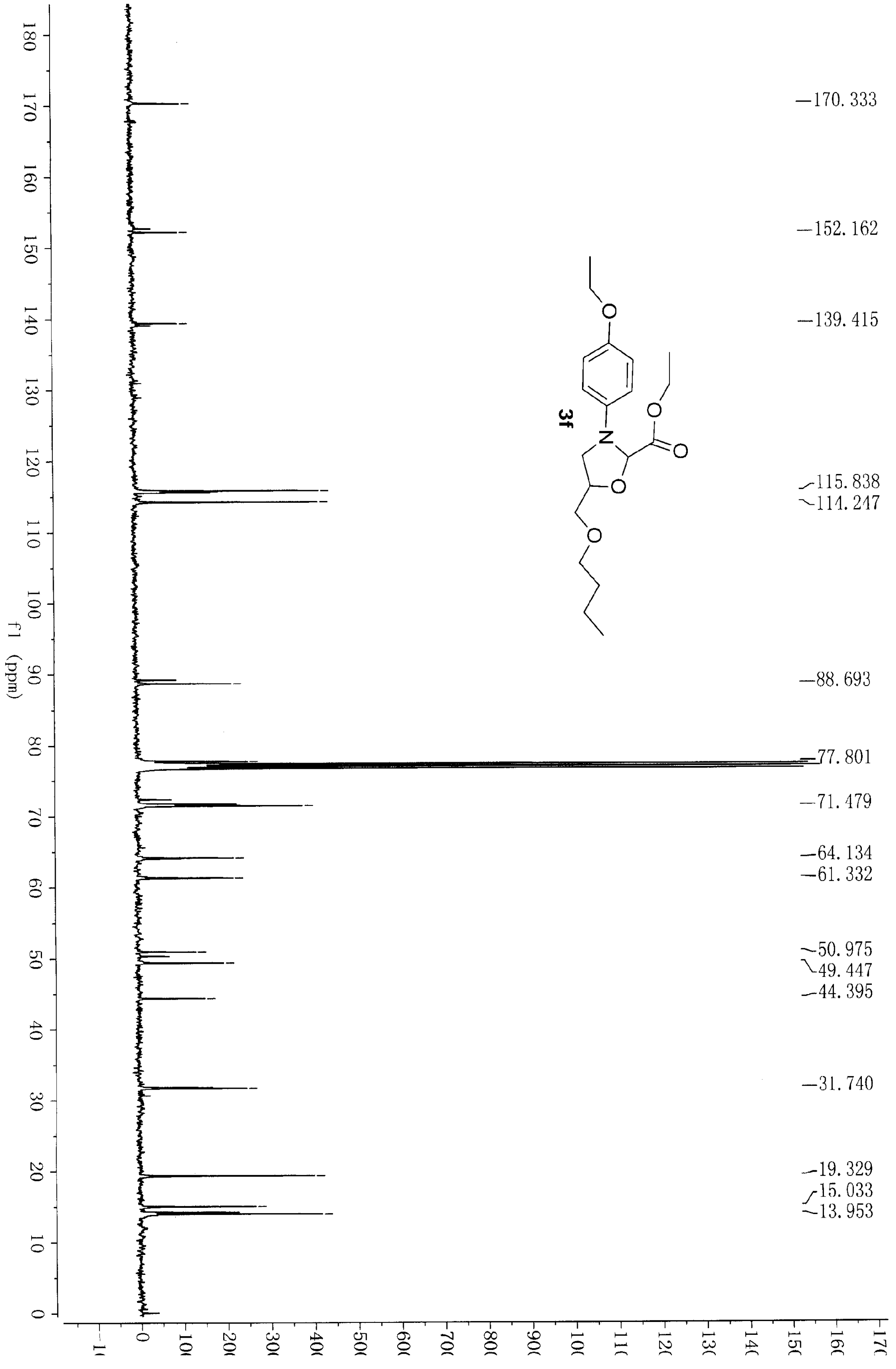

Figure S21. ^{13}C-NMR spectrum of product **3f**.

$C_{19}H_{29}NO_5$

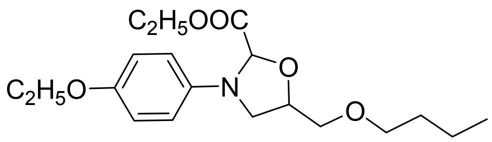

3f

3f. $C_{19}H_{29}NO_5$ **HRMS** (ESI$^+$) exact mass calculated for $[M + Na]^+$ requires *m*/*z* 374.1944, found *m*/*z* 374.1935.

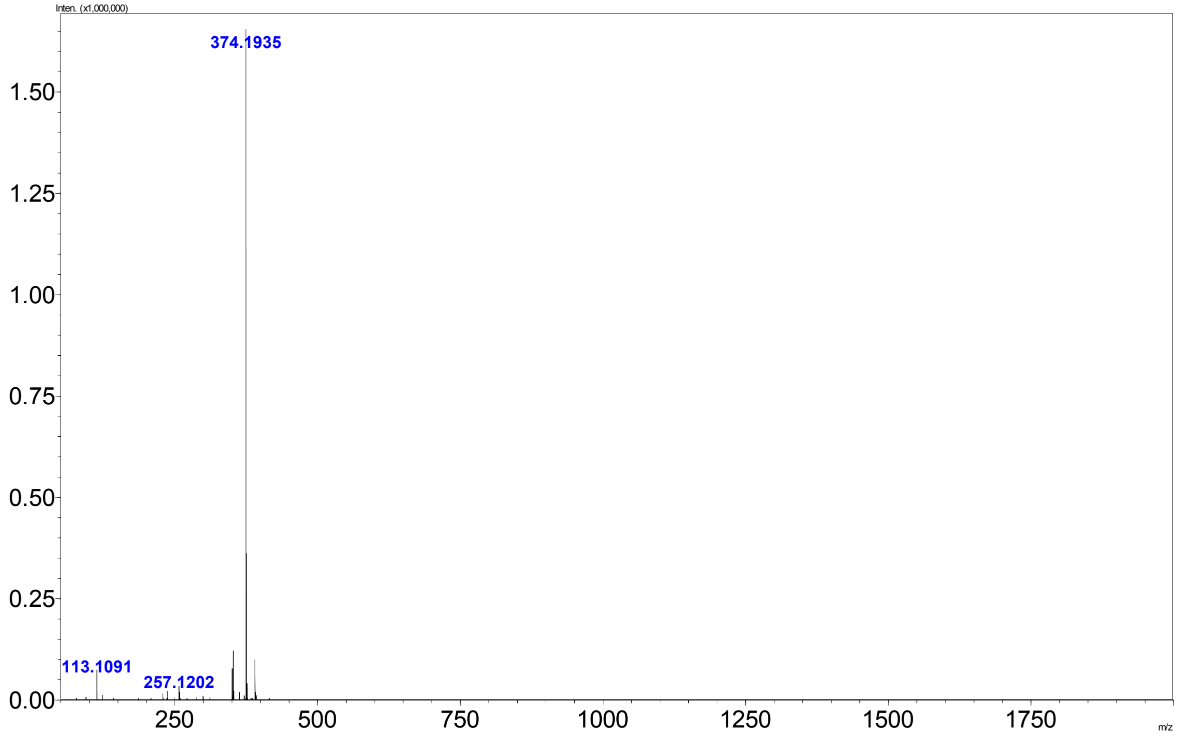

Figure S22. HRMS spectrum of product **3f**.

Table S6. HPLC analysis of product **3f**.

Column	CHIRALCEL OD-H(ODH0CE-LA084)
Column size	0.46 cm I.D.*25 cm L
Injection	20 µL
Mobile phase	*n*-Hexane/2-propanol = 95/5 (v/v)
Flow rate	1.0 mL/min
Wave length	UV 254 nm
Temperature	25 °C
Solvents	Hexane, 2-propanol: HPLC grade

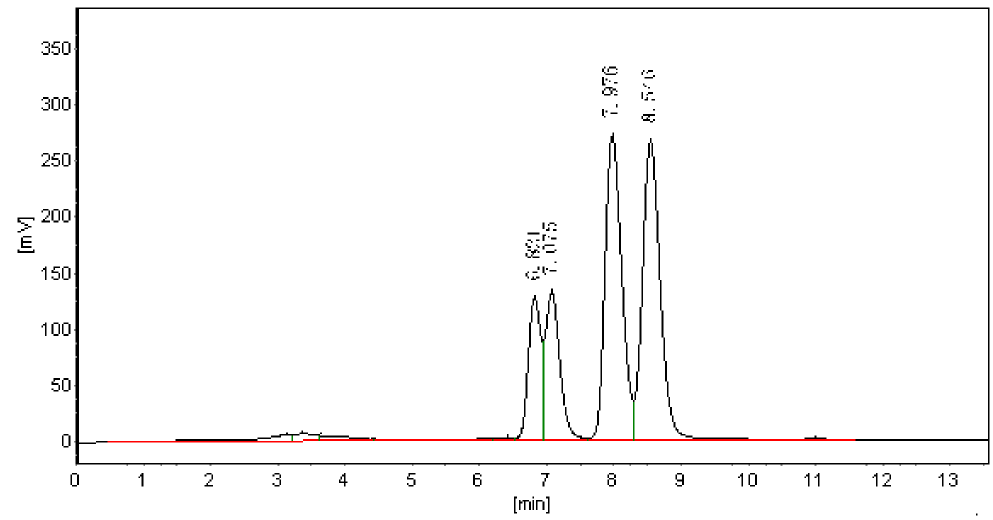

Peak NO.	Time	Height	Area	Area %
1	6.821	126366	1579316	11.93
2	7.075	131640	2007824	15.17
3	7.976	271027	4695282	35.47
4	8.546	266883	4955090	37.43

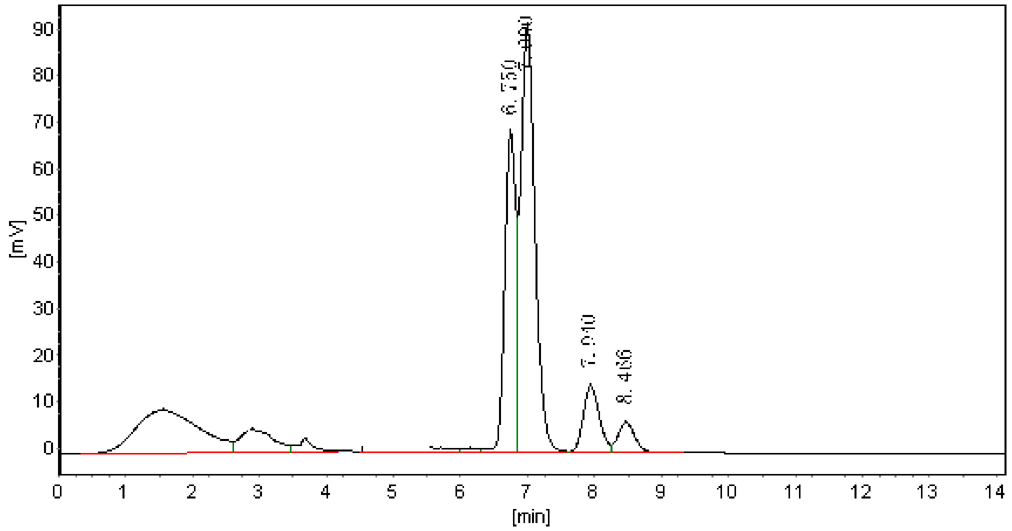

Peak NO.	Time	Height	Area	Area %
1	6.750	68622	806312	32.11
2	7.000	91562	1349758	53.73
3	7.940	14209	238947	9.52
4	8.466	6420	116979	4.64

Figure S23. *Cont.*

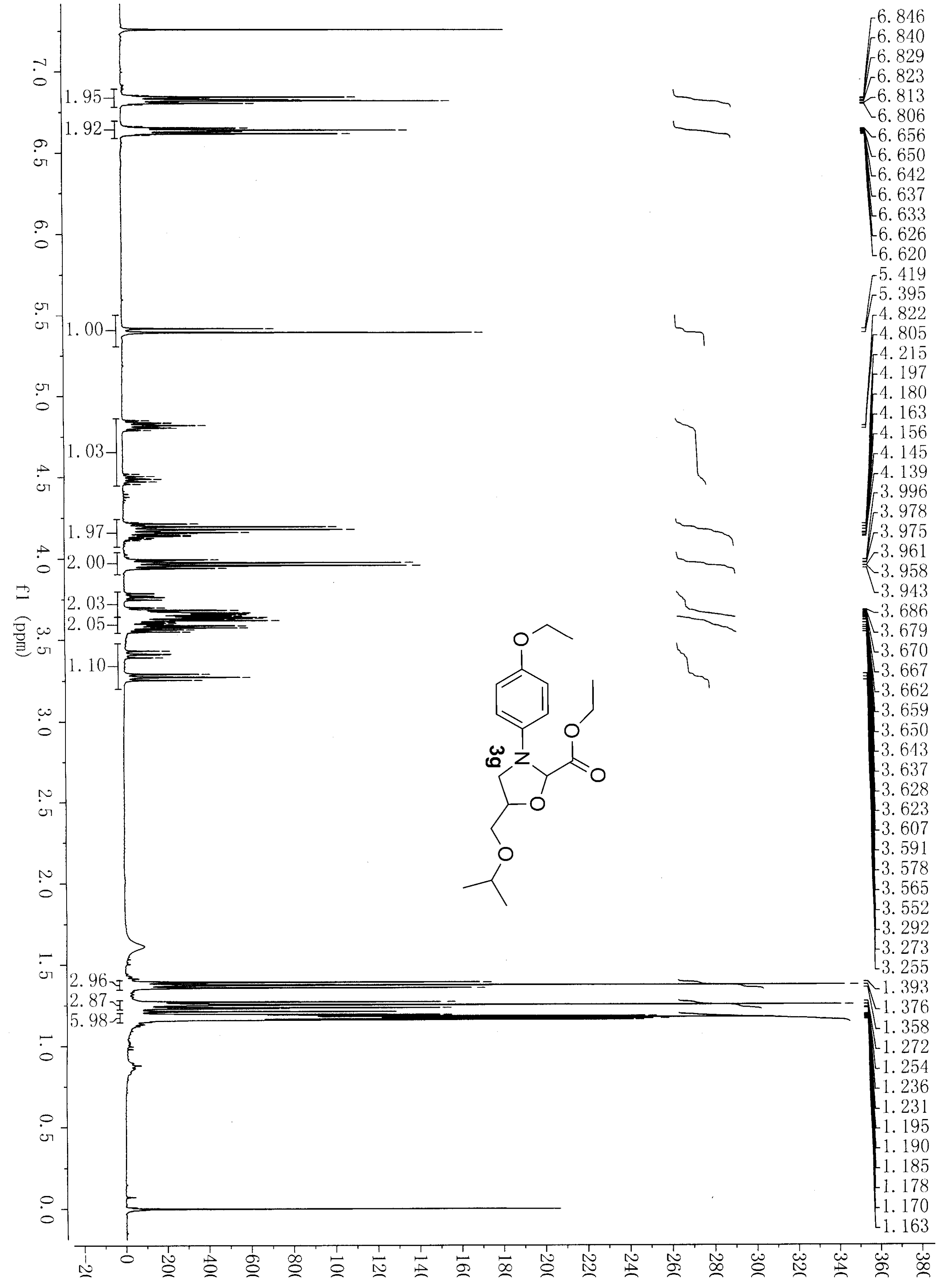

Figure S23. ^{1}H-NMR spectrum of product **3g**.

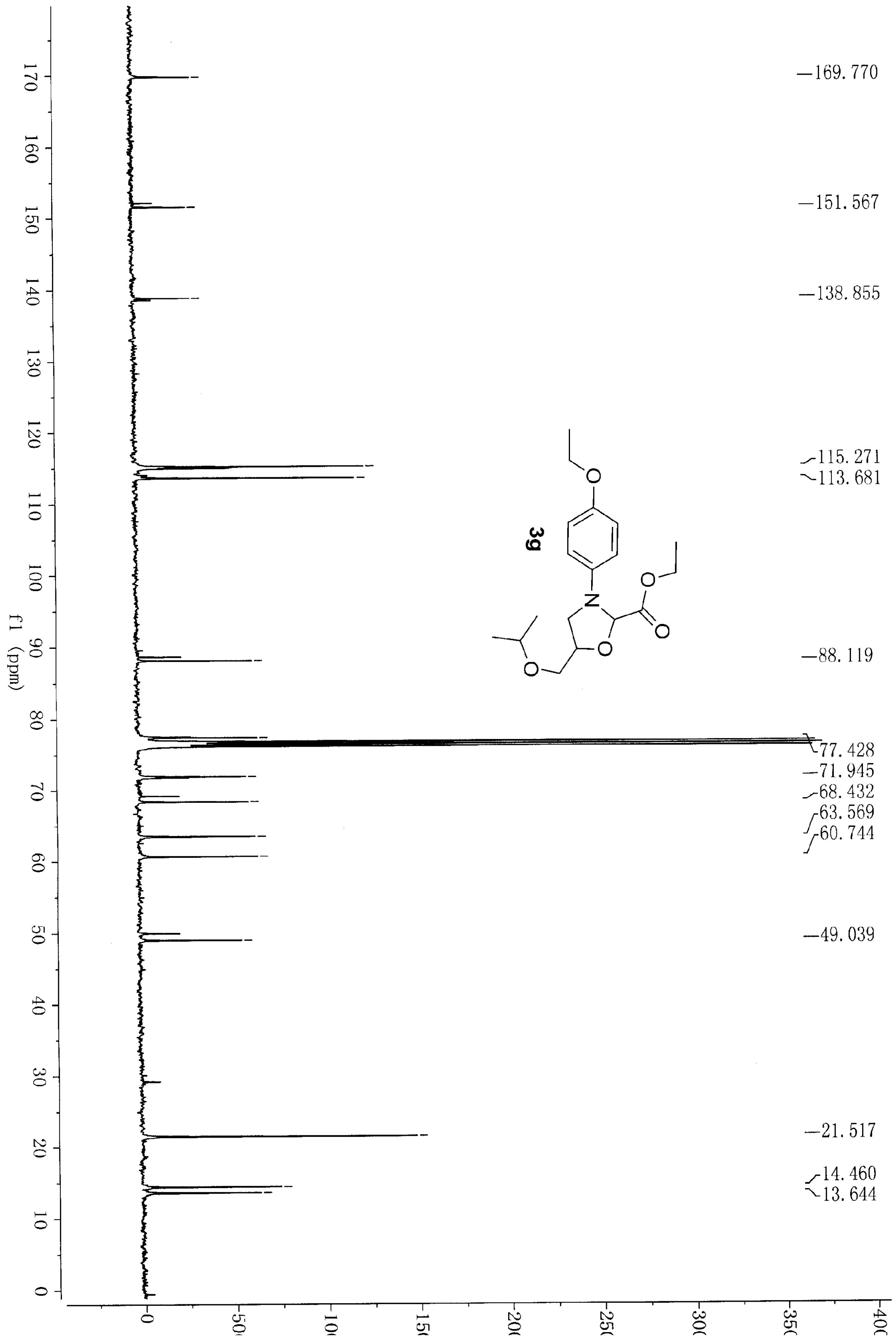

Figure S24. ^{13}C-NMR spectrum of product **3g**.

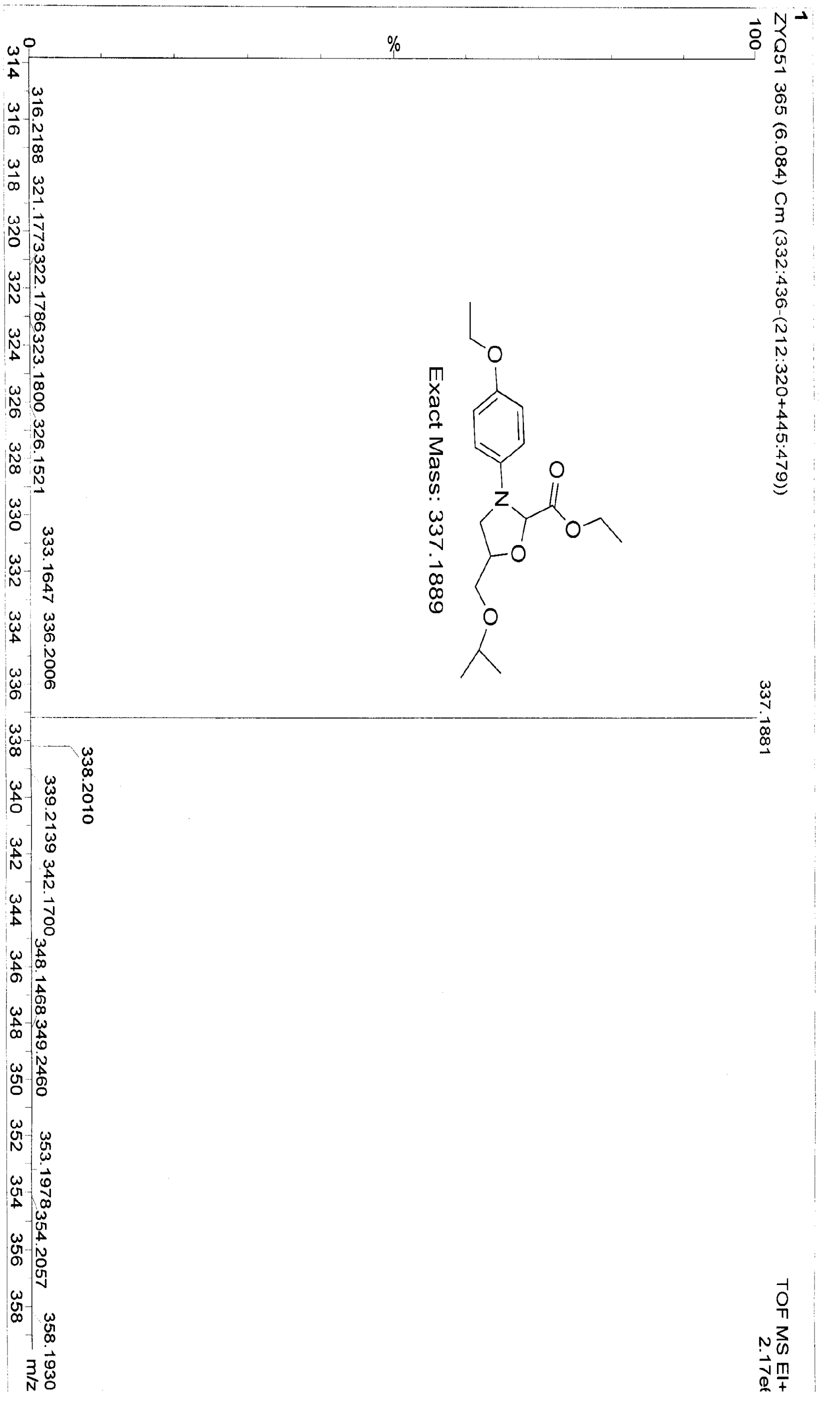

Figure S25. HRMS spectrum of product **3g**.

Table S7. HPLC analysis of product **3g**.

Column	CHIRALCEL OD-H(ODH0CE-LA084)
Column size	0.46 cm I.D.*25 cm L
Injection	20 μL
Mobile phase	*n*-Hexane/2-propanol = 95/5 (v/v)
Flow rate	1.0 mL/min
Wave length	UV 254 nm
Temperature	25 °C
Solvents	Hexane, 2-propanol: HPLC grade

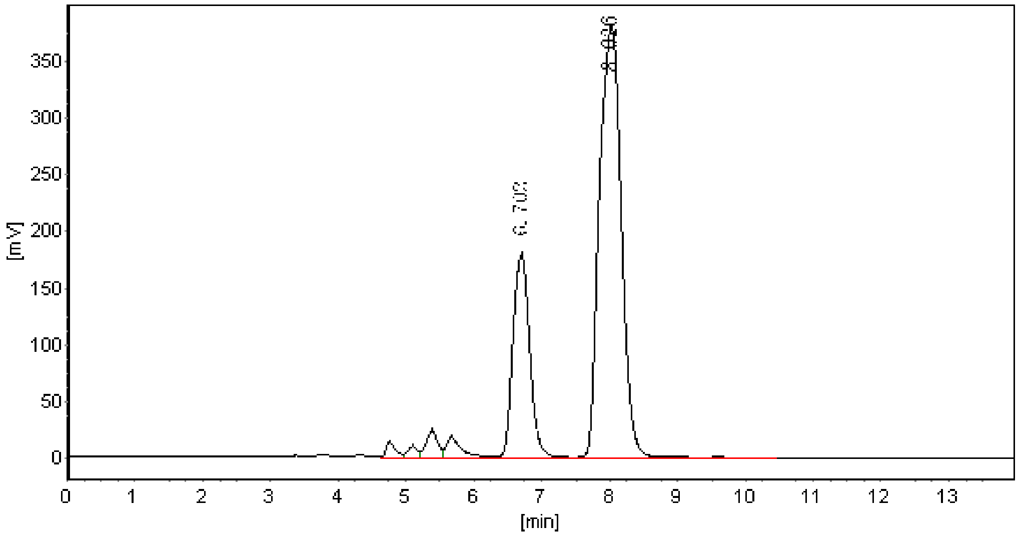

Peak NO.	Time	Height	Area	Area %
1	6.702	178710	3079137	26.59
2	8.026	380203	8504169	73.41

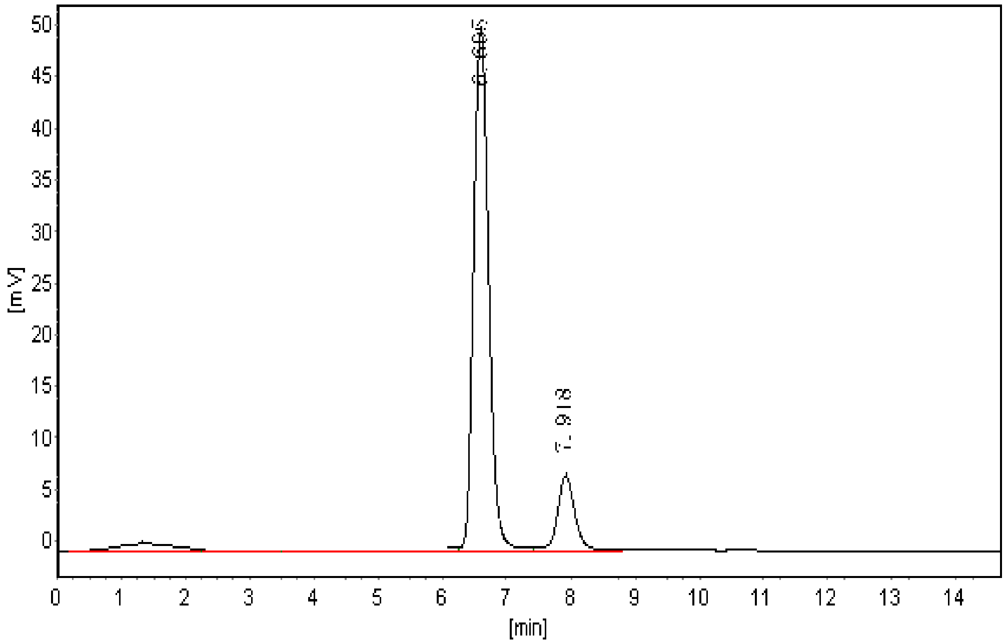

Peak NO.	Time	Height	Area	Area %
1	6.585	93190	1513932	85.7
2	7.897	13316	252162	14.3

Figure S26. *Cont.*

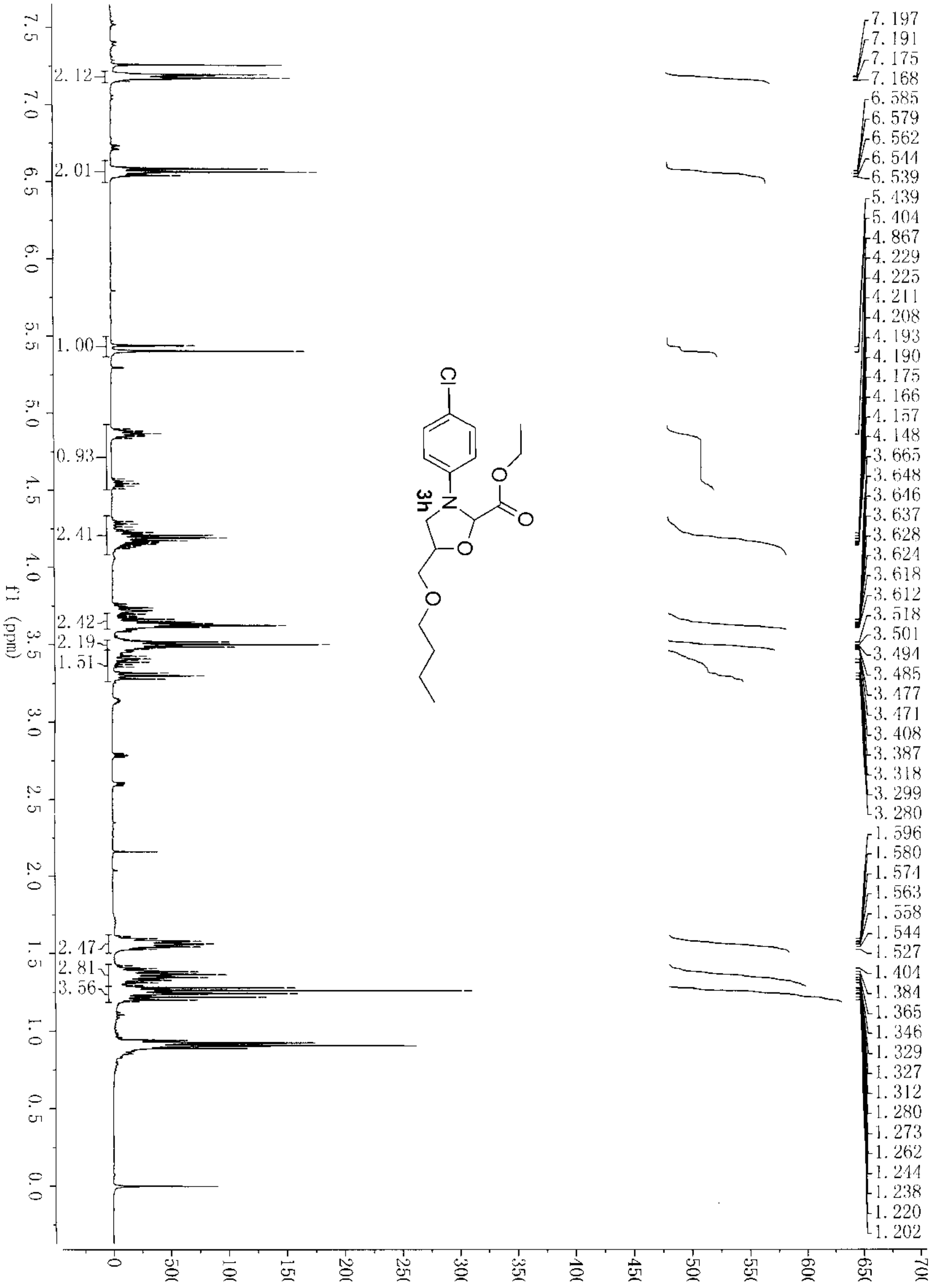

Figure S26. ^{1}H-NMR spectrum of product **3h**.

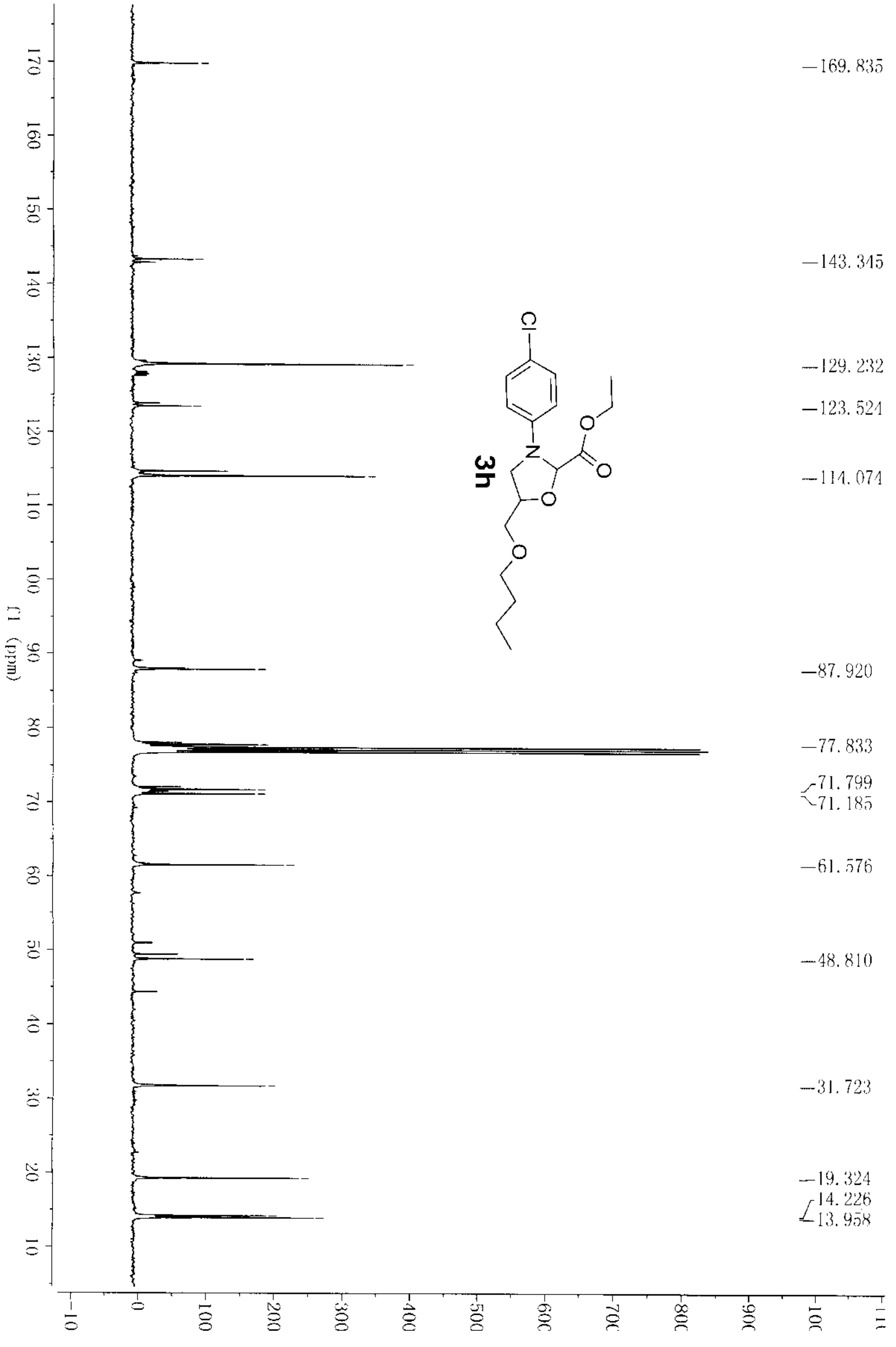

Figure S27. ^{13}C-NMR spectrum of product **3h**.

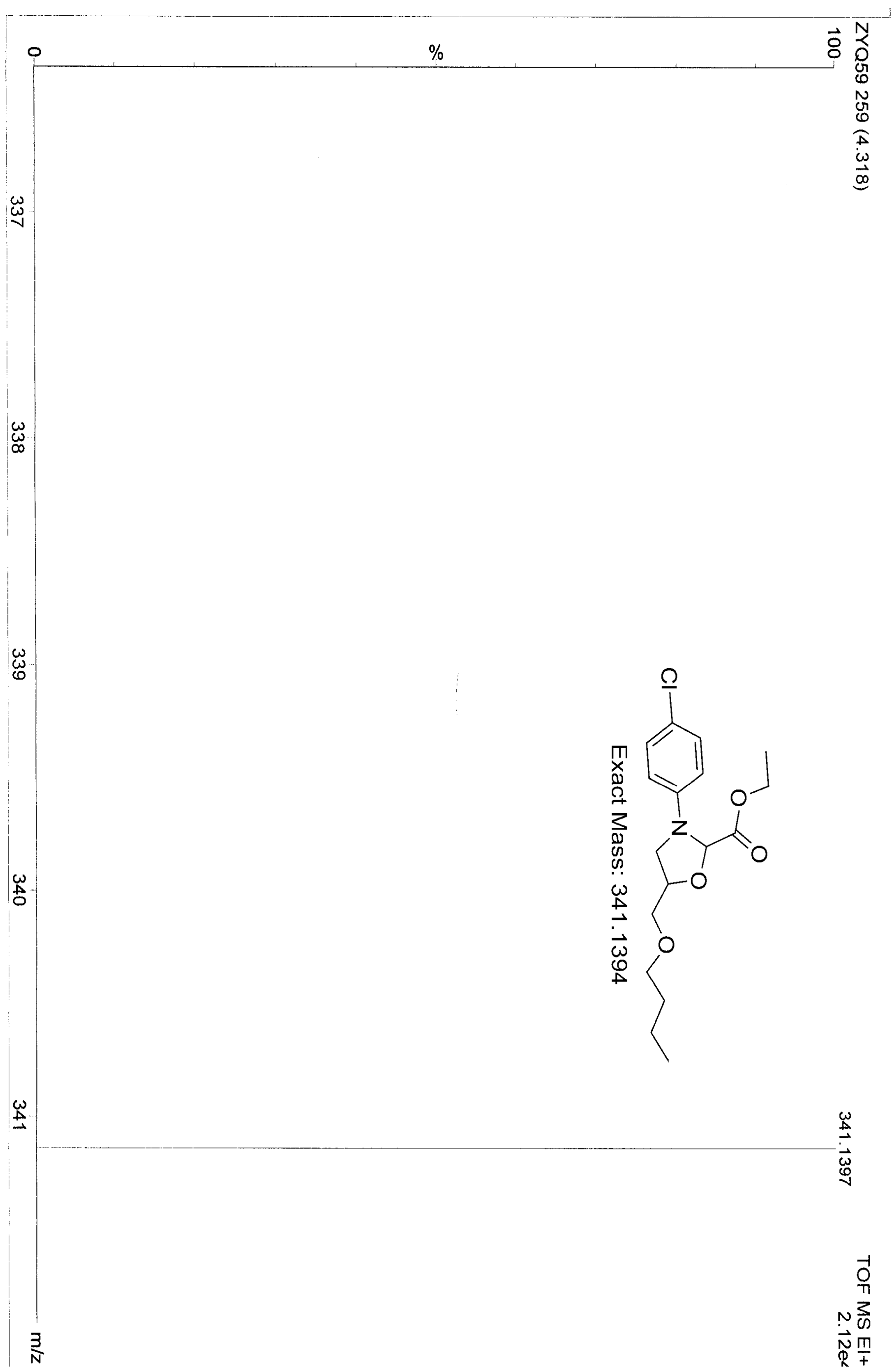

Figure S28. HRMS spectrum of product **3h**.

Table S8. HPLC analysis of product **3h.**

**Column**	**CHIRALCEL OD-H(ODH0CE-LA084)**
Column size	0.46 cm I.D.*25 cm L
Injection	20 μL
Mobile phase	*n*-Hexane/2-propanol = 95/5 (v/v)
Flow rate	1.0 mL/min
Wave length	UV 254 nm
Temperature	25 °C
Solvents	Hexane, 2-propanol: HPLC grade

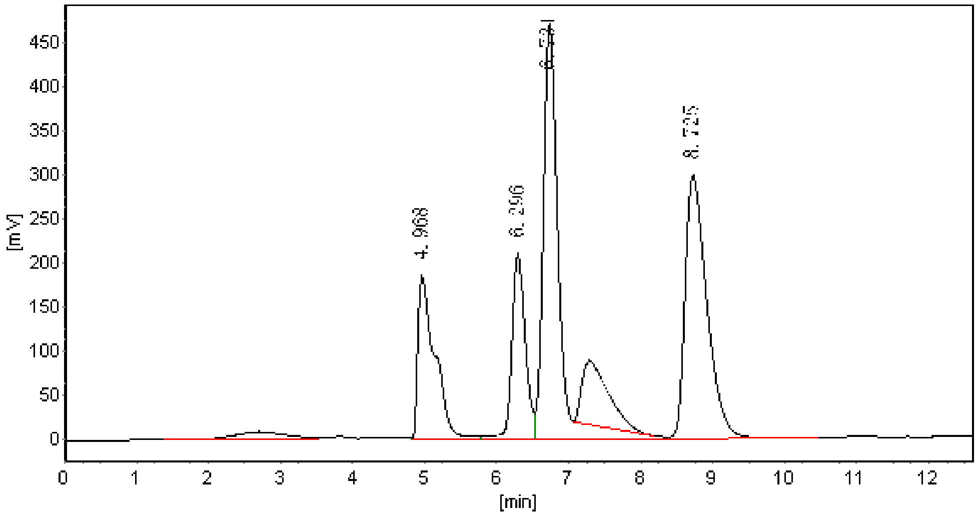

Peak NO.	Time	Height	Area	Area %
1	4.968	183302	3043118	16.52
2	6.296	208548	2630844	13.66
3	6.731	468928	7144385	37.12
4	8.725	297325	6294946	32.70

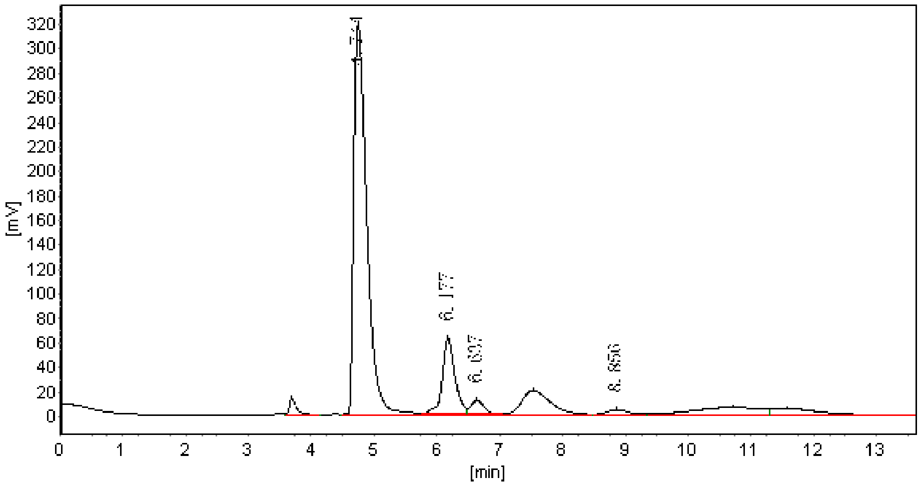

Peak NO.	Time	Height	Area	Area %
1	4.744	318552	4673525	80.79
2	6.177	61621	844018	14.59
3	6.637	11124	154946	2.68
4	8.856	4477	112447	1.94

Figure S29. *Cont.*

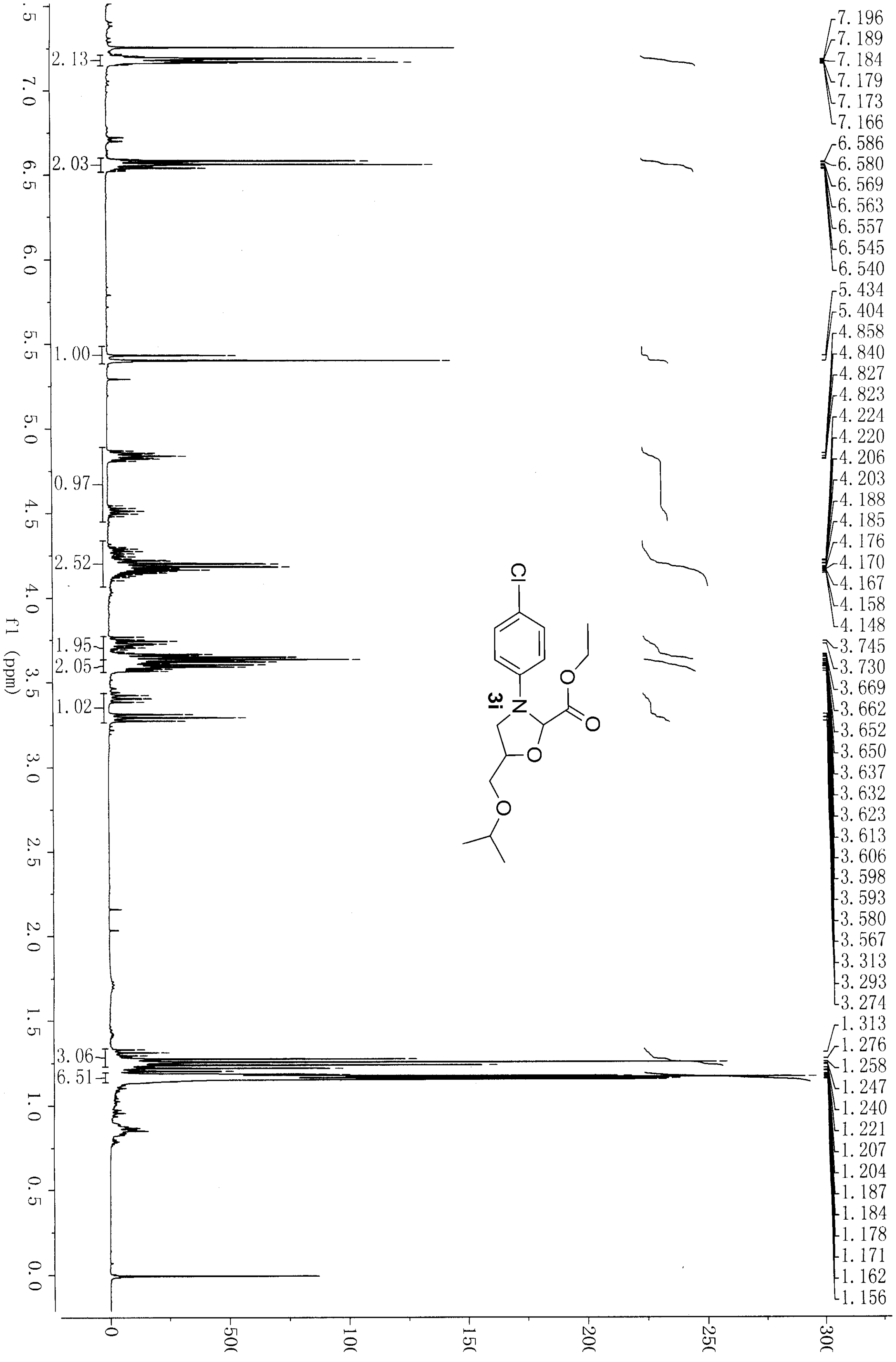

Figure S29. ^{1}H-NMR spectrum of product **3i**.

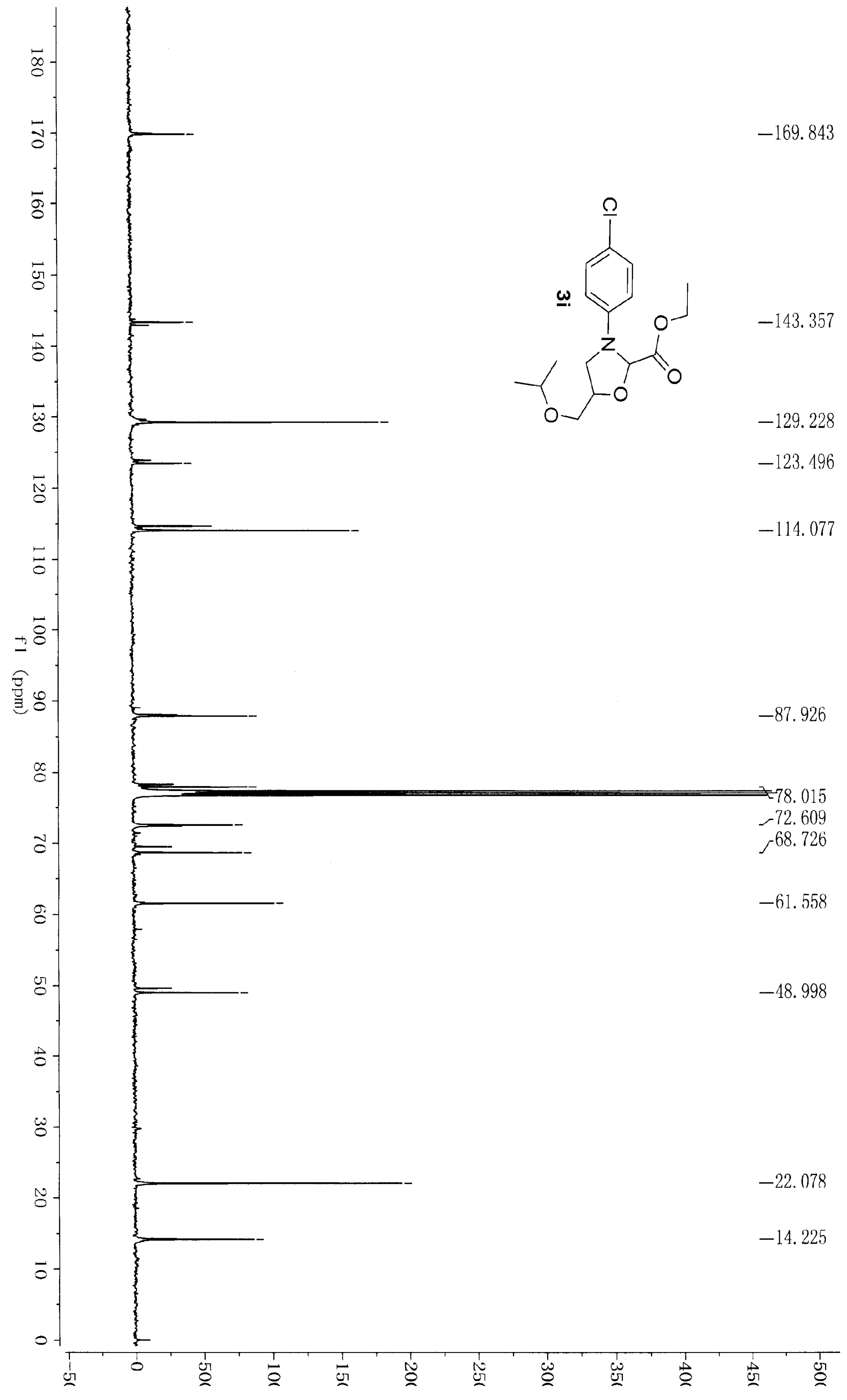

Figure S30. ^{13}C-NMR spectrum of product **3i**.

$C_{16}H_{22}ClNO_4$

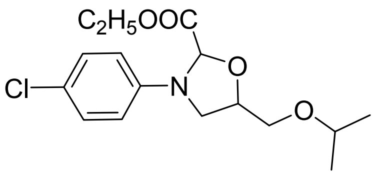

3i

3i: $C_{16}H_{22}ClNO_4$ **HRMS** (ESI$^+$) exact mass calculated for $[M + Na]^+$ requires *m*/*z* 350.1130, found *m*/*z* 350.1131.

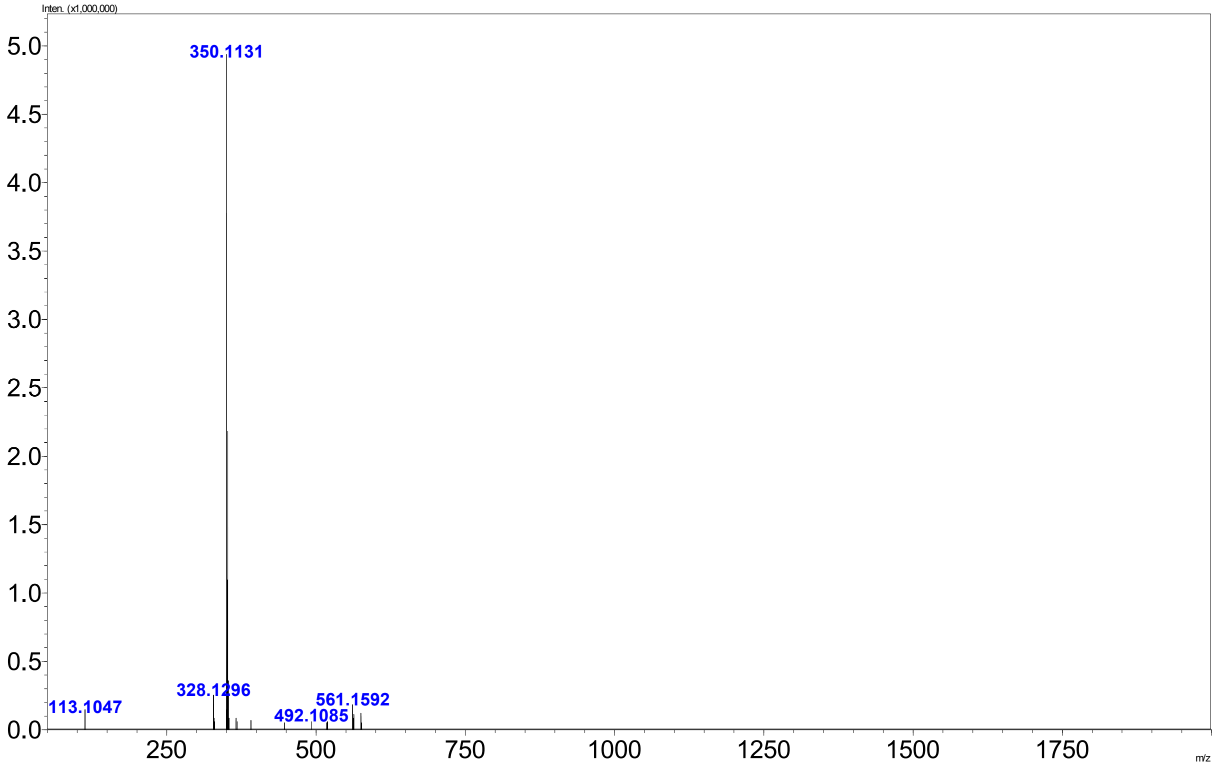

Figure S31. HRMS spectrum of product **3i**.

Table S9. HPLC analysis of product **3i**.

Column	CHIRALCEL OD-H(ODH0CE-LA084)
Column size	0.46 cm I.D.*25 cm L
Injection	20 µL
Mobile phase	*n*-Hexane/2-propanol = 95/5 (v/v)
Flow rate	1.0 mL/min
Wave length	UV 254 nm
Temperature	25 °C
Solvents	Hexane, 2-propanol: HPLC grade

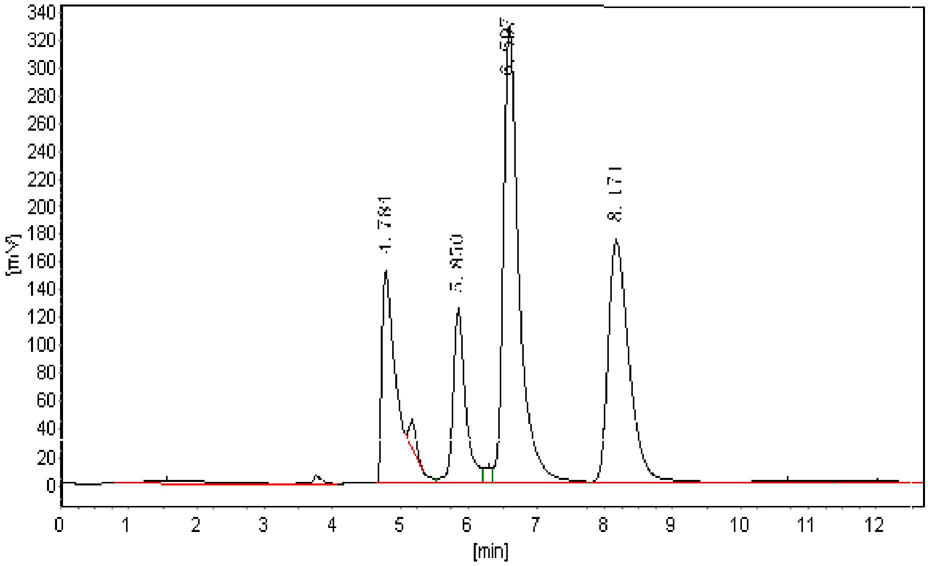

Peak NO.	Time	Height	Area	Area %
1	4.784	150143	2384941	18.40
2	5.850	121948	1570749	12.12
3	6.597	327757	5244969	40.47
4	8.171	174393	3759813	29.01

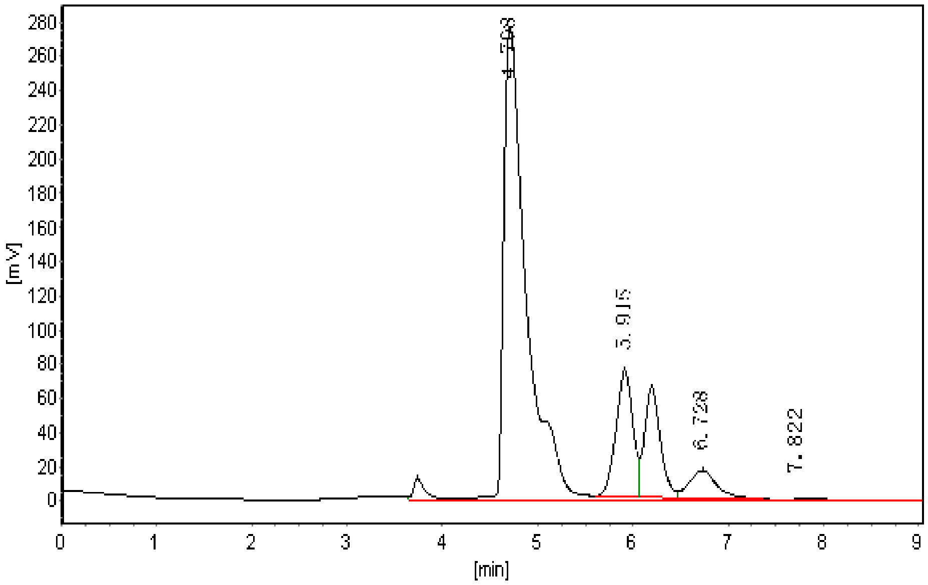

Peak NO.	Time	Height	Area	Area %
1	4.708	276083	4713534	80.89
2	5.915	74044	909622	15.61
3	6.728	16023	326582	5.60
4	7.822	598	16699	0.29

Figure S32. *Cont.*

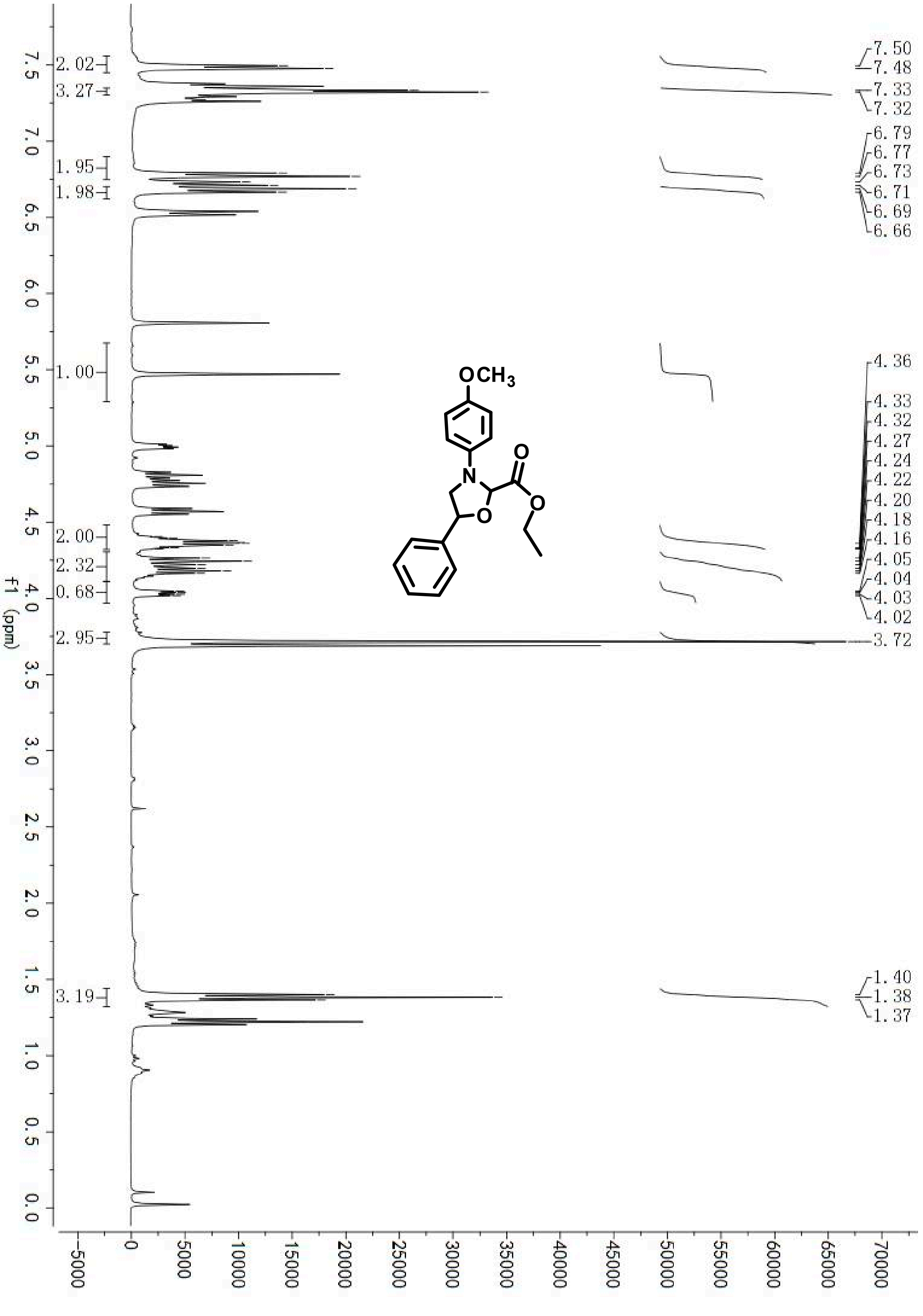

Figure S32. ^{1}H-NMR spectrum of product **3j**.

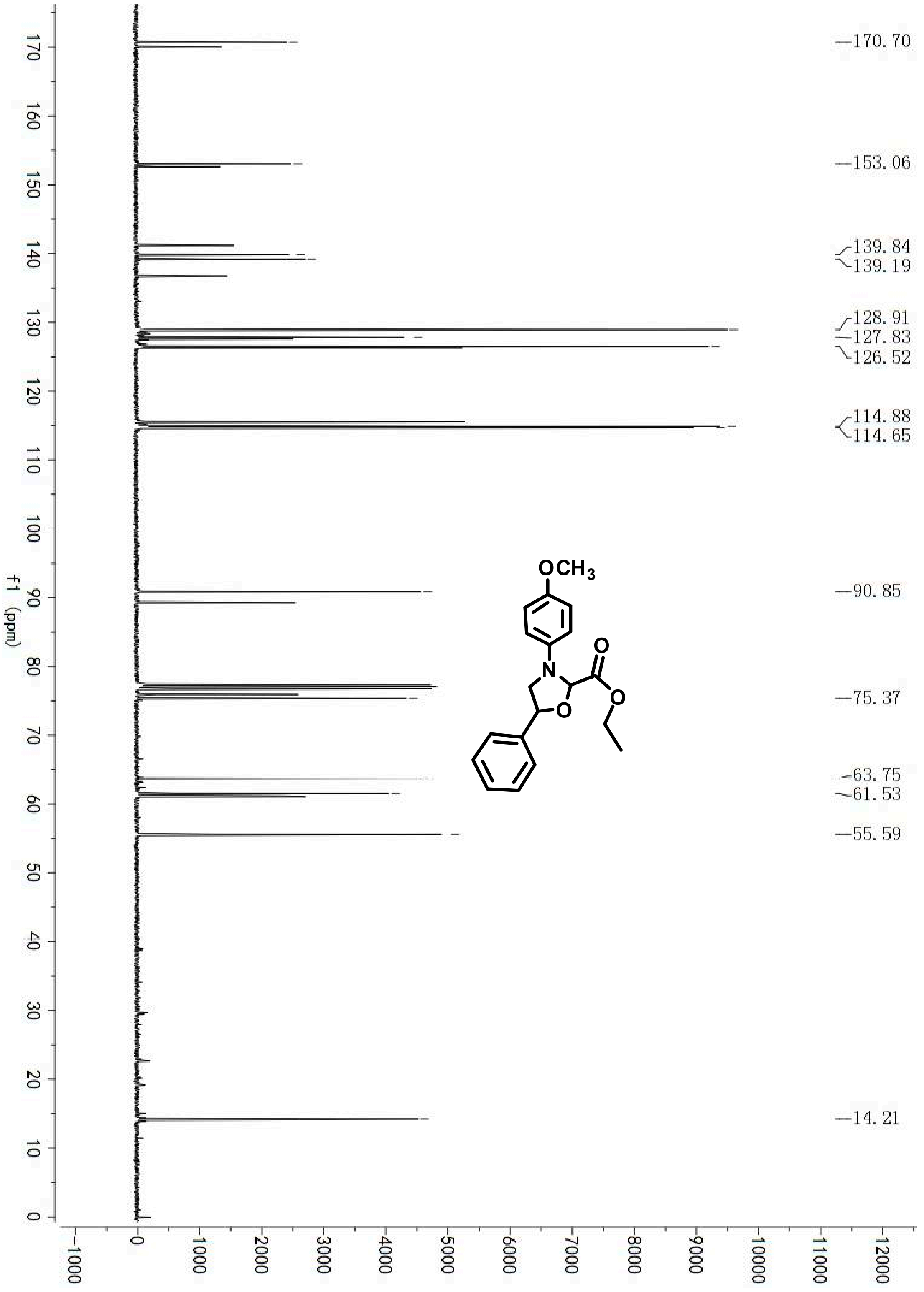

Figure S33. ^{13}C-NMR spectrum of product **3j**.

Elemental Composition Report **Page 1**

Tolerance = 2.0 mDa / DBE: min = -1.5, max = 50.0
Element prediction: Off

Monoisotopic Mass, Odd and Even Electron Ions
190 formula(e) evaluated with 1 results within limits (up to 50 closest results for each mass)
Elements Used:
C: 0-40 H: 0-50 N: 0-3 O: 0-12
ZYQ72 553 (9.218) Cm (553:554)
TOF MS EI+

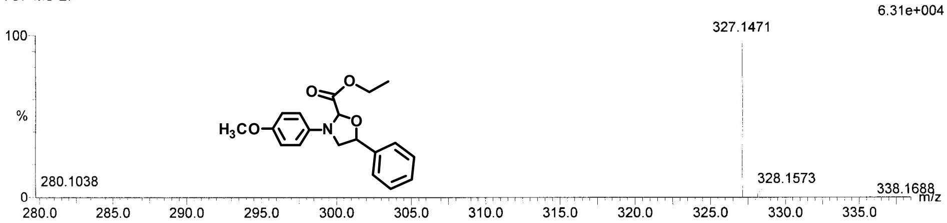

Minimum:	80.00				-1.5		
Maximum:	100.00		2.0	10.0	50.0		
Mass	RA	Calc. Mass	mDa	PPM	DBE	i-FIT	Formula
327.1471	100.00	327.1471	0.0	0.0	10.0	29436.0	C19 H21 N O4

Figure S34. HRMS spectrum of product **3j**.

Table S10. HPLC analysis of product **3j**.

Column	CHIRALCEL OD-H(ODH0CE-LA084)
Column size	0.46 cm I.D.*25 cm L
Injection	20 μL
Mobile phase	*n*-Hexane/2-propanol = 99/1 (v/v)
Flow rate	0.30 mL/min
Wave length	UV 254 nm
Temperature	25 °C
Solvents	Hexane, 2-propanol: HPLC grade

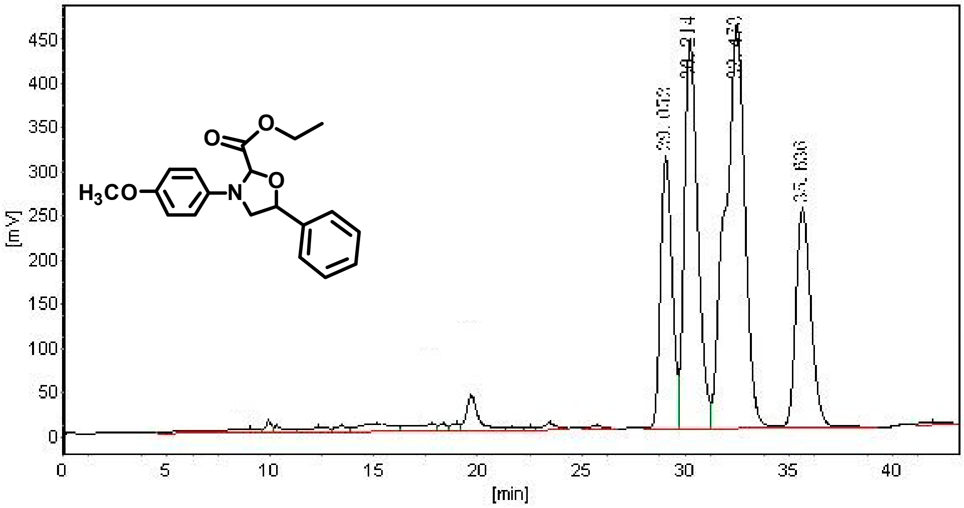

Figure S35. HRMS spectrum of product **3j**.

Table S11. HPLC analysis of product **3j**.

Peak NO.	Time	Height	Area	Area%
1	29.052	307,163	11,977,399	16.07
2	30.214	440,021	19,850,261	26.63
3	32.473	456,516	30,064,715	40.33
4	35.636	246,908	12,657,319	16.97

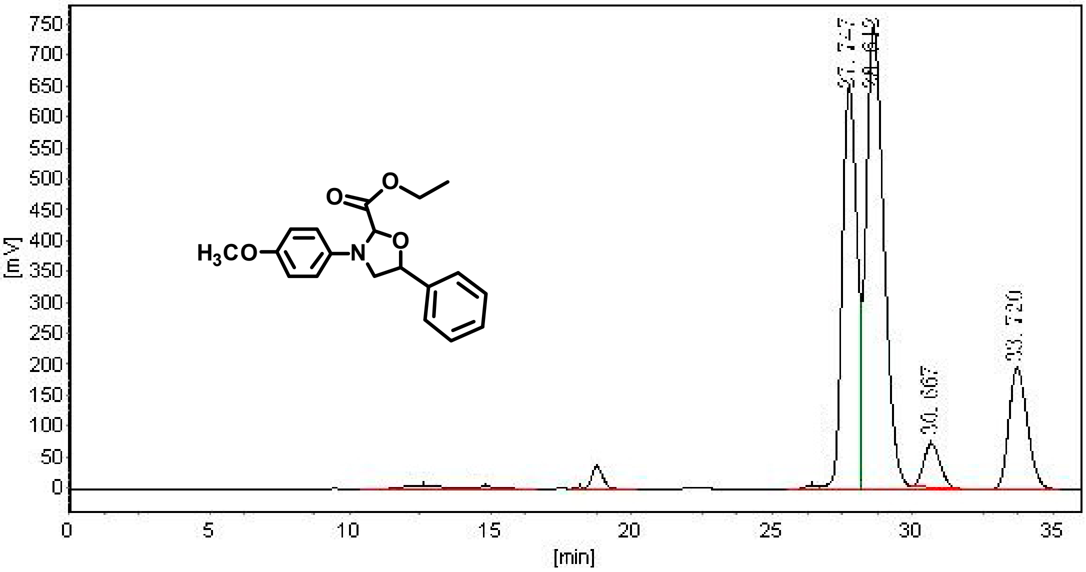

Figure S36. HRMS spectrum of product **3j**.

Table S12. HPLC analysis of product **3j**.

Peak NO.	Time(min)	Height	Area	Area%
1	27.747	652,411	23,859,811	34.40
2	28.612	747,186	33,725,572	48.62
3	30.667	70,090	2,814,584	4.06
4	33.720	194,969	8,960,056	12.92

5. HPLC Analysis for Epoxystyrene

Table S13. HPLC analysis for epoxystyrene (racemic).

Column	CHIRALCEL OD-H(ODH0CE-LA084)
Column size	0.46 cm I.D.*25 cm L
Injection	20 μL
Mobile phase	*n*-Hexane/2-propanol = 95/5 (v/v)
Flow rate	0.5 mL/min
Wave length	UV 254 nm
Temperature	25 °C
Solvents	Hexane, 2-propanol: HPLC grade

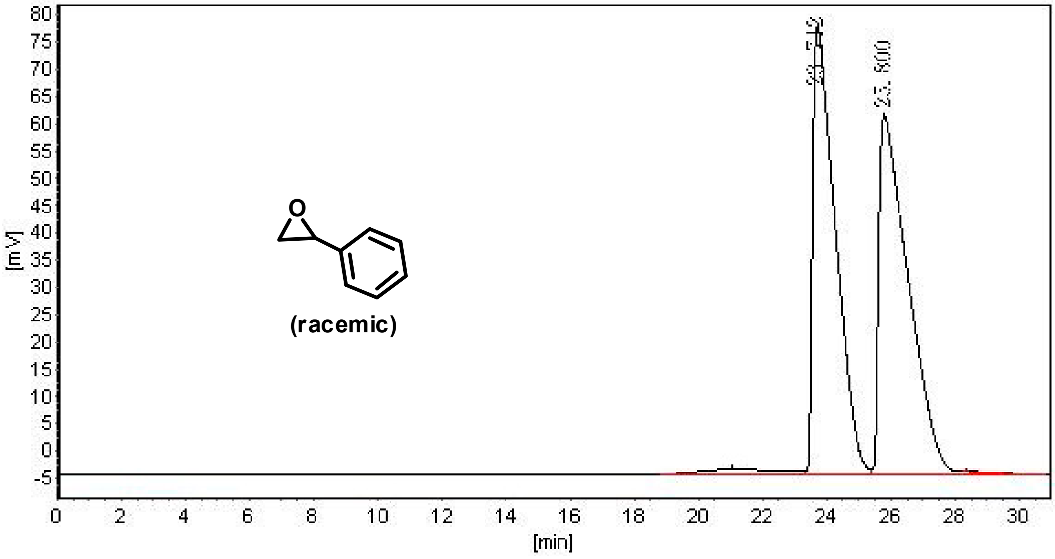

Figure S37. HPLC analysis for epoxystyrene (racemic).

Table S14. HPLC analysis for epoxystyrene (racemic).

Peak NO.	Time	Height	Area	Area%
1	23.712	81,939	4,057,522	49.12
2	25.800	65,677	4,203,137	50.88

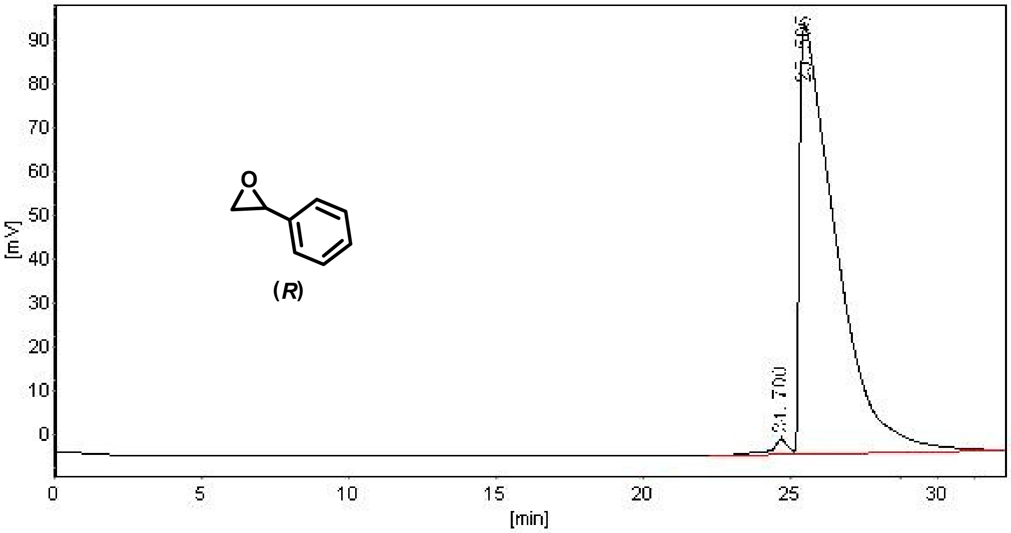

Figure S38. HPLC analysis for (*R*)-epoxystyrene.

Table S15. HPLC analysis for (*R*)-epoxystyrene.

Peak NO.	Time	Height	Area	Area%
1	24.700	3302	138,449	3.25
2	25.505	97,642	8,387,937	96.75

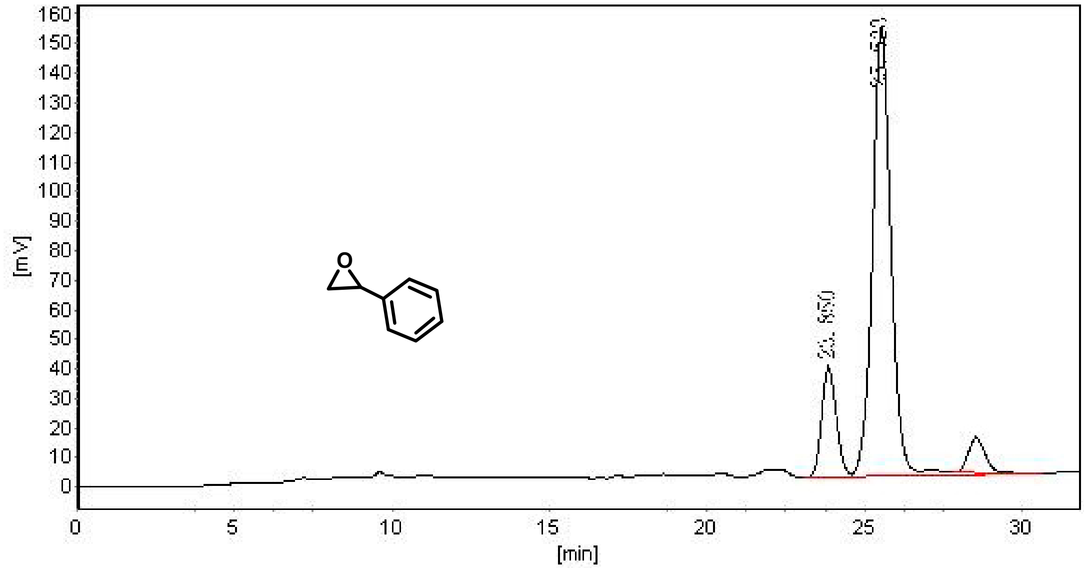

Figure S39. HPLC analysis for epoxystyrene (reclaimation from the system).

Table S16. HPLC analysis for epoxystyrene (reclaimation from the system).

Peak NO.	Time	Height	Area	Area%
1	23.850	36,923	1,203,119	16.05
2	25.523	15,166	6,291,923	83.95

6. HMBC Spectrum of Product 3a

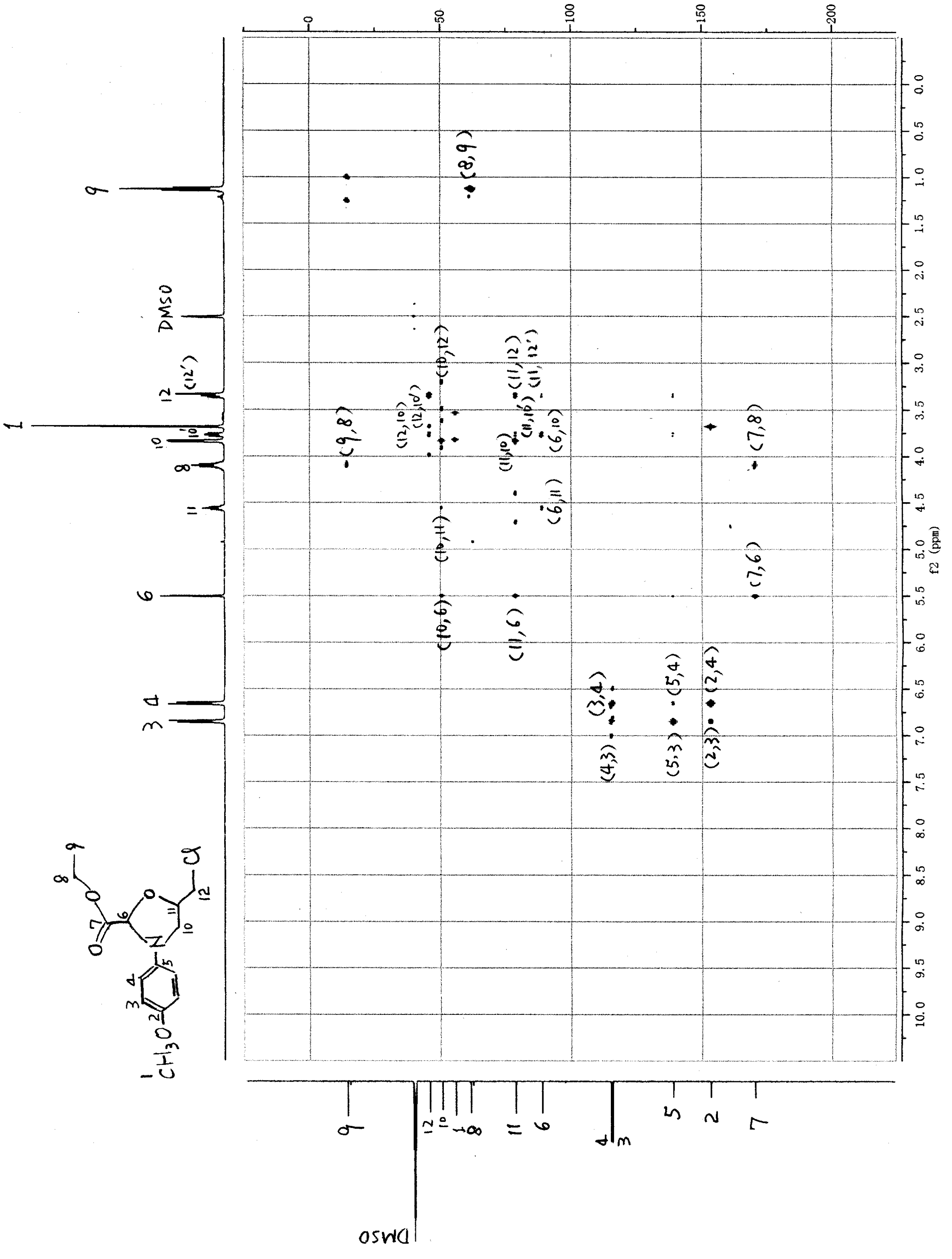

Figure S40. HMBC Spectrum of Product 3a.

7. NOESY Spectrum of Product 3a

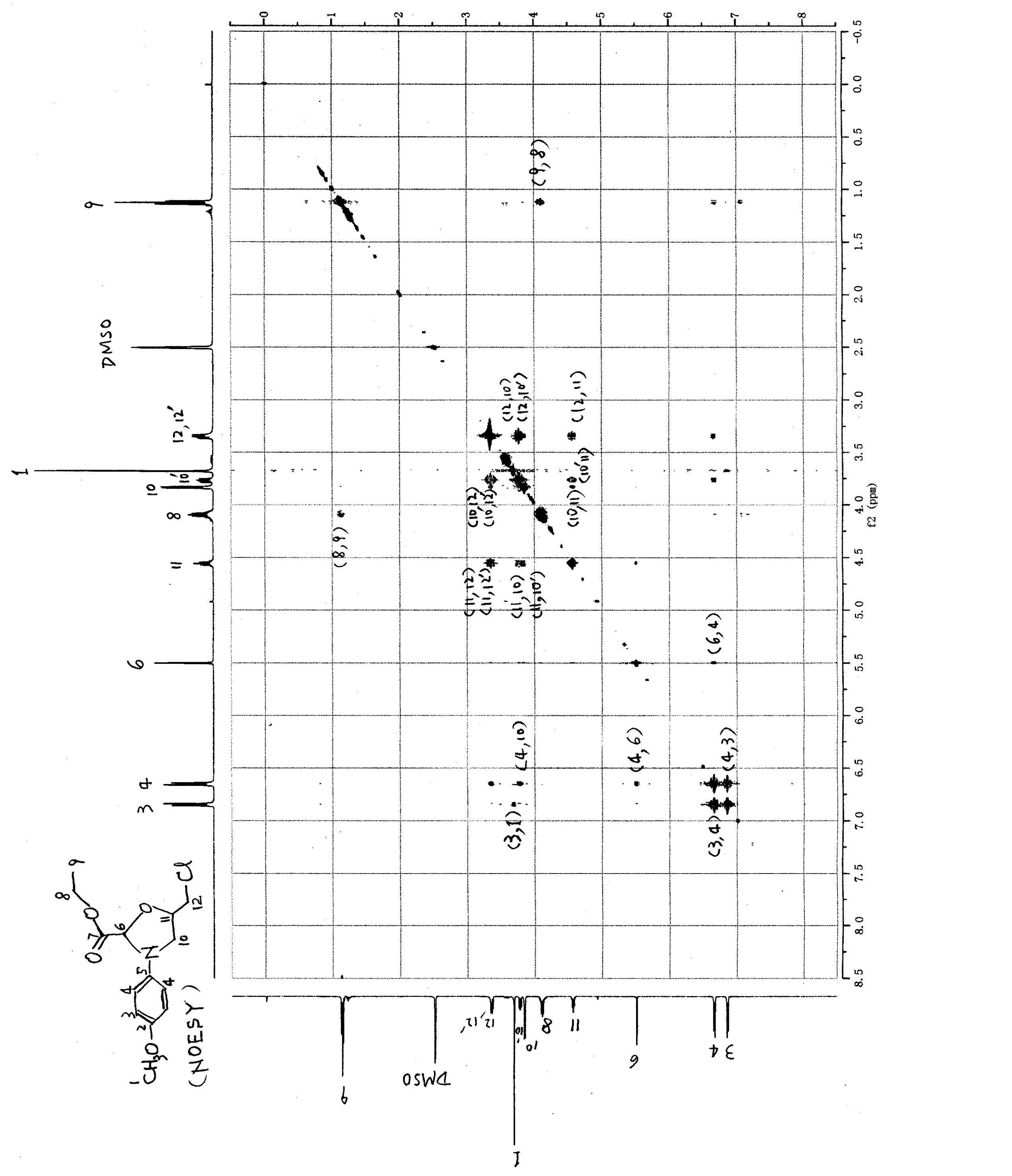

Figure S41. NOESY Spectrum of Product **3a**.
